# The role of involved field irradiation versus elective nodal irradiation in definitive radiotherapy or chemoradiotherapy for esophageal cancer- a systematic review and meta-analysis

**DOI:** 10.3389/fonc.2022.1034656

**Published:** 2022-11-02

**Authors:** Hesong Wang, Chunyang Song, Xiaohan Zhao, Wenzhao Deng, Wenbin Shen

**Affiliations:** Department of Radiation Oncology, Fourth Hospital of Hebei Medical University, Shijiazhuang, Hebei Province, China

**Keywords:** involved field irradiation, elective nodal irradiation, IFI, ENI, radiotherapy, esophageal neoplasms, esophageal carcinoma, meta-analysis

## Abstract

**Objective:**

This study aimed to analyze whether involved field irradiation (IFI) is associated with improving survival outcomes and reducing adverse events compared with elective nodal irradiation (ENI) in patients of esophageal cancer who underwent definitive radiotherapy or chemoradiotherapy.

**Summary background data:**

Radiotherapy plays an important role for not surgery patients. However, the role of radiation target size is still uncertain.

**Methods:**

We searched Web of Science, Embase, PubMed, and Cochrane Central for English and non-English publications comparing esophageal cancer patients who received radiotherapy with IFI with those with ENI. Primary outcomes included overall survival (OS) and adverse events related to radiotherapy. The risk of bias was assessed using the Cochrane Risk of Bias tool for randomized studies and the Newcastle-Ottawa Scale and Agency for Healthcare Research and Quality Standard for non-randomized studies. We evaluated the certainty of evidence by Grading of Recommendations, Assessment, Development, and Evaluation.

**Results:**

Totally, 23 studies with 4120 patients were included. IFI group demonstrated significant improvement in the OS rates at 5 years, but not at 1, 2, and 3 years, compared with the ENI group (pooled Risk Ratio [RR], 0.78; 95% confidence interval [CI], 0.68–0.90; P = 0.0004; high certainty). In addition, IFI demonstrated a significant decrease in the incidence of grade ≥2 acute esophagitis (AE) (pooled RR, 0.79; 95% CI, 0.69–0.90; P = 0.0005; high certainty) and grade ≥3 AE (pooled RR, 0.51; 95% CI, 0.38–0.69; P < 0.00001; high certainty) compared with ENI, but not in the incidence of grades ≥3 acute pneumonia, late esophagitis, and late pneumonia.

**Conclusions:**

Compared to ENI, IFI demonstrated significant improvement in OS at 5 years. The addition of intensity-modulated radiotherapy (IMRT) to IFI increased the 5-year OS; however, similar results were not observed with the addition of three-dimensional conformal radiotherapy to IFI and ENI. Furthermore, IFI demonstrated a significant decrease in grade ≥2 and grade ≥3 AE, while IMRT demonstrated no difference in the incidence of grade ≥3 AE. IFI and ENI do not differ in the incidence of grades ≥3 acute pneumonia, late esophagitis, and late pneumonia.

## Introduction

Esophageal cancer results in more than half a million cancer-related deaths worldwide each year (1). It ranked sixth in the main cause of cancer-related death, ranked seventh in the incidence of tumor (2). Squamous cell carcinoma and adenocarcinoma are two main subtypes of esophageal cancer, which occupies the majority of all (3, 4). Many esophageal cancers were unresectable, and most eventually returned after radical treatment (5–7). Most patients are diagnosed with late staged disease, not suitable for surgery (8). Radiotherapy is important in the treatment of esophageal cancer. The RTOG 85-01 trial demonstrated that definitive chemoradiotherapy (CRT) is recommended for not surgery patients (9).

Optimal depiction of radiation therapy targets is essential to improve treatment effectiveness and reduce radiotoxicity (10). To reduce tumor metastasis, the usual practice is to provide irradiation to an area that has not been metastasized, called elective lymph node irradiation (ENI). ENI improved local area control but did not improve overall survival (OS). In addition, there is a corresponding increase in treatment-related adverse events. With the progress of treatment technology, the target size of radiotherapy can be reduced to a certain extent. Involved-field irradiation (IFI), which irradiates only the affected area, is a method to reduce the volume of irradiation. For IFI, one common radiation target is that the gross tumor volume (GTV) is the primary focus of esophageal cancer plus metastatic lymph nodes; the clinical target volume (CTV) is the normal esophagus outlined 3 cm above and below the GTV, and the metastatic lymph nodes are not outwardly placed in the CTV; the planned target volume (PTV) is the CTV outwardly expanded 1 cm in all directions. The method of determining the clinical CTV for the primary tumor is much the same in various countries (11–13). The modalities available for determining the CTV, especially the lymph node volume, vary. The treatment modality of ENI advocates irradiation of normal areas of non-metastatic lymph nodes that are also included in the CTV. Thus, the method of setting the CTV is still uncertain. However, In the last few decades, many studies have explored the impact of target volume on clinical prognosis. Some studies suggested that IFI can improve the prognosis of patients (14–17), whereas other studies favored ENI over IFI (18–21).

Moreover, no large prospective RCTs compared the treatment outcomes of IFI with ENI in esophageal cancer patients. Therefore, this systematic review and meta-analysis aimed to explore whether IFI is more beneficial than ENI in terms of survival and incidence of adverse events in a large group of population.

## Methods

### Search strategy

The medical databases, namely, Web of Science, Embase, PubMed, and Cochrane Central, were searched for publications that do not distinguish between languages(last update: April 30, 2022). The search strategy is summarized in **Supplementary Table 1**. This study was proceeded based on the Preferred Reporting Items for Systematic Reviews and Meta-Analyses (PRISMA).

### Literature selection

Only those studies that investigated the role of IFI and ENI in definitive radiotherapy or chemoradiotherapy for esophageal cancer were eligible for inclusion. We included RCTs and retrospective studies. Exclusion criteria included (1): studies that investigated either IFI or ENI alone (2), palliative rather than curative radiotherapy (3), unpublished data (4), case reports, conference abstracts, meta-analysis, ongoing clinical trials, academic papers, editorials, letters, review papers, comments, and basic science articles (5); no correlation results; and (6) full text not available. We did not discriminate against articles by language.

### Study selection and data extraction

Two authors (authors Chunyang Song and Xiaohan Zhao) evaluated each article separately and extract relevant information. If there was any difference in the process, the other person (author Wenzhao Deng) would resolve it. We extracted relevant information from the included studies: baseline study characteristics (author, year, country, and study type), sample size, follow-up time, study period, age, tumor location, pathological type, clinical stage, radiation dose, radiation technology, chemotherapy regimens, and relevant outcomes data.

### Outcomes

The primary outcomes in this review were 1-, 2-, 3-, and 5-year OS rates and adverse events related to radiotherapy. Secondary outcomes included 1-, 2-, 3-, and 5-year progression-free survival (PFS) rates and 1-, 2-, and 3-year local control rates (LCRs).

### Quality assessed

We evaluated randomized studies by the Cochrane Risk of Bias tool and non-randomized studies by the Newcastle-Ottawa Scale (NOS) and Agency for Healthcare Research and Quality (AHRQ) Standard. The scores were from 0 to 9. A score above 6 was considered high quality. Authors Chunyang Song and Xiaohan Zhao scored the included studies respectively. If there was any dispute, Wenzhao Deng would be asked to settle it. We evaluated the quality of the results by the Cochrane Grading of Recommendations Assessment, Development, and Evaluation (GRADE) methodology.

### Statistical analysis

This study was conducted by the software of Cochrane Review Manager, version 5.4 (London, UK). Survival curves were read by Engauge Digitizer, version 12.1 (available from: http://markummitchellgithubio/engauge-digitizer/). Heterogeneity was evaluated by I^2^ statistic. If I^2^ ≤50%, which indicated no significant heterogeneity among the studies, a fixed-effects model was used; otherwise, a random-effects model was employed. Publication bias was assessed using a funnel plot for results that included more than 10 studies. The significance level of the results was set to P <0.05. Subgroup analyses were performed based on the study type (RCTs and non-RCTs), radiotherapy used (three-dimensional conformal radiotherapy [3D-CRT], intensity-modulated radiotherapy [IMRT], and 3D-IMRT–mixed for patients who received both 3D-CRT and IMRT), pathology (esophageal squamous cell carcinoma [ESCC] and ESCC-mixed in patients with both ESCC and non-ESCC), and type of chemotherapy (CCRT—patients who received concurrent chemoradiotherapy; CCRT+CT—patients who received concurrent chemoradiotherapy with consolidated chemotherapy, and CRT-mixed—patients who received radiotherapy with or without chemotherapy).

## Results

### Study characteristics

**Figure 1:**
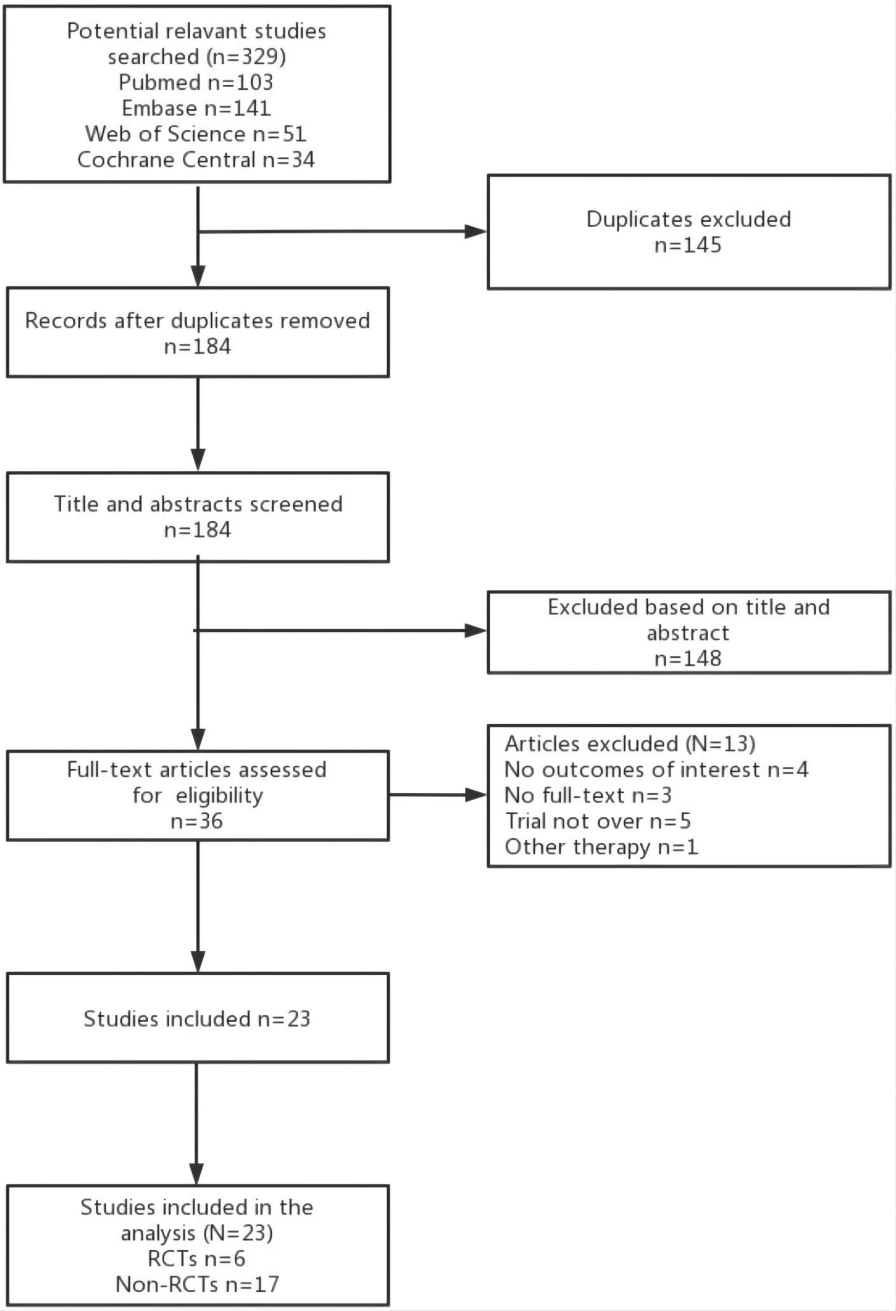
PRISMA diagram.

329 potential studies were retrieved, ultimately, we included 23 studies. After removing duplicate studies, 184 records underwent screening. In total, 36 articles were assessed for eligibility, and, finally, 23 (22–44) studies with 4120 patients were ultimately included in this study, including 6 RCTs (23, 25, 29, 41, 43, 44) and 17 non-RCTs (22, 24, 26–28, 30–40, 42) (**Figure 1**).

All studies were performed in Asian countries (including 20 studies from China, 2 from Japan, and 1 from Korea). Of the 4120 patients, 2279 received IFI and 1841 received ENI. The study publication time ranged from 2011 to 2020. The study period ranged between 2000 to 2017. The median age of the patients ranged from 56.8–75.0 years, and the follow-up duration ranged between 1 and 188 months. Cancer type included ESCC (97.6%; n = 4022) and non-ESCC (2.4%; n = 98). Notably, 18 studies enrolled ESCC patients only, while the other 5 studies included patients with both ESCC and non-ESCC. Tumor locations included the cervical and upper, middle, and lower thoracic regions. Only one study included one specific tumor location, and eight studies included various tumor locations. The stage included I to IV. Only one study included one specific stage, and five studies included tumors with various TNM stages. The most commonly used radiotherapeutic modalities were 3D-CRT and IMRT. The radiation dose delivered ranged from 38 to 72 Gy, and the per fraction ranged from 1.6 to 2.5 Gy. Patients in one study received radiotherapy alone, whereas those in the other 4, 6, and 12 studies received CCRT, CCRT+CT, CRT-mixed, respectively. The detailed treatment are summarized in **Supplementary Table 2**, and the summary of outcomes are detailed in **Supplementary Tables 3** and **4**. The characteristics are presented in **Table 1**.

**Table 1:** Study Characteristics.

Study	Study type	Country	Radiotherapy target size	N analyzed	Histology	Time Range	Age (yr,SD/range)	Follow-up Time Interval (mo,range)	Location	Overallstage (1/2/3/4)
Zhu, 2020	Non-RCT	China	IFI	272	ESCC:240 non-ESCC:32	2006.1-2015.12	64.4 ± 11.3	85.9 (77.4-94.5)	c:16 ut:115 mt:98 lt:43	1:48 2:61 3:92 4:71
			ENI	272	ESCC:247 non-ESCC:25		64.0 ± 8.5	85.9 (77.4-94.5)	c:32 ut:119 mt:89 lt:32	1:61 2:55 3:89 4:67
Xie, 2020	RCT	China	IFI	88	ESCC	2007.12-2015.6	62 (41–70)	–	c:6 ut:26 mt:49 lt:7	–
			ENI	88			61 (40-68)	–	c:6 ut:26 mt:48 lt:8	–
Nakatani, 2020	Non-RCT	Japan	IFI	78	ESCC	2000.1-2012.12	66.5(44-82)	62 (19-188)	u/mt:61 lt:17	1:78
			ENI	117			68(49-84)	111.5 (3-185)	u/mt:79 lt:38	1:117
Lyu, 2020	RCT	China	IFI	98	ESCC	2012.4-2016.10	≥60:54 <60:44	–	ut:42 mt:49 lt:7	2:27 3:71
			ENI	94			≥60:44 <60:50	–	ut:37 mt:49 lt:8	2:22 3:72
Q.F Li, 2019	Non-RCT	China	IFI	314	ESCC	2006.1-2012.12	≥62:181(57.6%) <62:133(42.4%)	117.6 (110.2-124.9)	u/mt:279 lt:35	1+2:174 3+4a:140
			ENI	157			≥62:84(53.5%) <62:73(46.5%)	92.9 (88.3-97.6)	u/mt:138 lt:19	1+2:83 3+4a:74
Wang, 2018	Non-RCT	China	IFI	276	ESCC	2008.1.4-2017.11.30	≥70 (70-80:225 ≥80:134)	–	c:16, ut:66 mt:175 lt:102	1:95 2:193 3:71
			ENI	83				–		
Sun, 2018	Non-RCT	China	IFI	49	ESCC	2005.1-2015.6	67(43-76)	48	ut:27 mt:14 lt:8	T2-3N0M0:31 T4N0M0:18
			ENI	77			64(39-75)		ut:39 mt:28 lt:10	T2-3N0M0:51 T4N0M0:26
Yisikandaer, 2018	RCT	China	IFI	104	ESCC	2012.4-2016.10	≥65:65 <65:39	28 (3-60)	ut:36 mt:59 lt:9	2:27 3:77
			ENI	101			≥65:70 <65:31		ut:47 mt:48 lt:6	2:22 3:79
Zhao, 2017	Non-RCT	China	IFI	40	ESCC	2007.11-2015.5	66(20-87)	19.4	c	1-2:15 3:7
			ENI	46						
Su, 2017	Non-RCT	China	IFI	47	ESCC:90 non-ESCC:6	2006.1-2011.12	73(65-82)	38 (16.8-105)	c+ut:5 mt:27 lt:15	1:18 2:29
			ENI	49					c+ut:15 mt:27 lt:7	1:23 2:26
Zh Jing, 2017	Non-RCT	China	IFI	38	ESCC:38 (100%) non-ESCC:0	2000.10-2005.12	<65:19 (50%) ≥65:19 (50%)	91.2 (2.4-131.7)	c:4 ut:9 mt:16 lt:9	2:20 3:13 4:5
			ENI	51	ESCC:49 (96%) non-ESCC:2 (4%)		<65:27 (52.9%) ≥65:24 (47.1)	123.1 (3.17-142.2)	c:1 ut:17 mt:26 lt:7	2:24 3:12 4:15
Park, 2016	Non-RCT	Korea	IFI	50	ESCC	2001.5-2013.5	69 (48-81)	20 (2-78)	ut:6 mt:26 lt:18	2:22 3:28
			ENI	49			65 (36-81)	22 (2-150)	ut:15 mt:23 lt:11	2:19 3:30
D.J Li, 2016	Non-RCT	China	IFI	43	ESCC	2008.1-2013.12	65.6(42-90)	32 (18-53)	ut:14 mt:23 lt:6	2:3 3:32 4:8
			ENI	36			56.8(43-75)		ut:10 mt:18 lt:8	2:2 3:25 4:9
Bai, 2016	Non-RCT	China	IFI	15	ESCC	2006.1.1-2012.8.1	63(39-77)	24	c:15 t:48	1:9 2:36 3:18
			ENI	48						
Dong, 2015	Non-RCT	China	IFI	119	ESCC:108 non-ESCC:11	2006.1-2012.12	>65:63 ≤65:56	21 (1.7-93.3)	c+ut:27 mt:64 lt:28	1:22 2:38 3:59
			ENI	126	ESCC:121 non-ESCC:5		>65:58 ≤65:68		c+ut:44 mt:63 lt:19	1:31 2:30 3:65
Yamashita , 2015	Non-RCT	Japan	IFI	119	ESCC:109(92%) non-ESCC:10(8%)	2000.6-2014.3	68(44-86)	18 (1-169)	c:6 ut:20 mt:61 lt:32	1:21 2:19 3:45 4:34
			ENI	120	ESCC:113(95%) non-ESCC:7(6%)		67(46-83)		c:4 ut:19 mt:56 lt:41	1:21 2:28 3:32 4:39
W Jing, 2015	Non-RCT	China	IFI	83	ESCC	2009.1-2013.3	75(70-88)	16.4 (3-66)	c:5 t:78	–
			ENI	54					c:6 t:48	–
Cao, 2015	Non-RCT	China	IFI	110	ESCC	2003.1.1-2009.12.31	>62:99 ≤62:59	39 (3-123)	c:12 ut:49 mt:67 lt:30	–
			ENI	48						–
Liu, 2014	Non-RCT	China	IFI	99	ESCC	2008.1.1-2010.12.31	62 (-73)	30 (14-59)	c:9 ut:90	1-2:49 3-4:50
			ENI	70			62 (-73)		c:9 ut:61	1-2:33 3-4:37
Zang, 2013	RCT	China	IFI	35	ESCC	2003.3.28-2007.4.2	61(41-72)	22.1 (1.5-48.6)	mt:35	–
			ENI	38			58(39-70)		mt:38	–
Shen, 2013	Non-RCT	China	IFI	102	ESCC	2000.10-2007.12	–	33.3 (1.5-100)	–	1:10 2:113
			ENI	21			–		ut:18 mt:2 lt:1	
M Li, 2012	RCT	China	IFI	49	ESCC	2006.5-2009.9	66(48-75)	19 (7.1-42)	ut:19 mt:23 lt:7	1+2:15 3:34
			ENI	45			62(45-75)		ut:19 mt:20 lt:6	1+2:16 3:29
Ma, 2011	RCT	China	IFI	51	ESCC	2004.9-2006.4	62(39-74)	37 (2-47)	c:33 ut:69	–
			ENI	51						–

IFI, Involved Field Irradiation; ENI, Elective Nodal Irradiation; ESCC, Esophageal squamous cell carcinoma; c, cervical; t, thoracic; ut, upper thoracic; mt, middle thoracic; lt, lower thoracic.

### OS Rates

**Figure 2:**
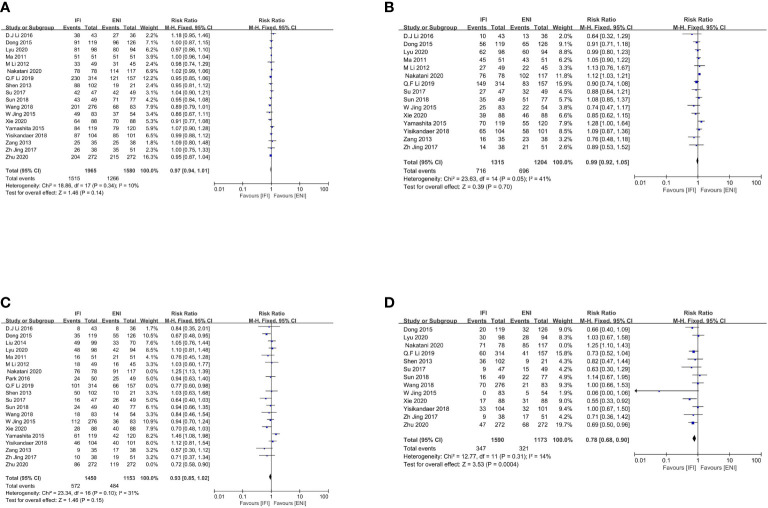
Forest plot of 1- **(A)**, 2- **(B)**, 3- **(C)** and 5-years **(D)** overall survival rate. IFI, involved field irradiation; ENI, elective nodal irradiation; M-H, Mantel-Haenszel; CI, confidence interval.

A total of 18 studies (22–29, 31, 32, 34, 36–38, 41–44) analyzed the 1-year OS rates (**Figure 2A**), including 6 RCTs (23, 25, 29, 41, 43, 44) and 12 non-RCTs (22, 24, 26–28, 31, 32, 34, 36–38, 42). No significant differences were observed between the IFI and ENI groups (pooled Risk Ratio [RR], 0.97; 95% confidence interval [CI], 0.94–1.01; P = 0.14, high certainty) with no significant heterogeneity (P = 0.34; I^2 =^ 10%). As the studies performed by Q. F. Li et al. (26) and Zhu et al. (22) contributed significantly and weighted similarly, we conducted a sensitivity analysis. Removing these did not change the 1-year OS rates. With regard to subgroup, the study type (**Supplementary Figure 1A** for RCTs group, and **Supplementary Figure 1B** for non-RCTs group), the type of radiotherapy (**Supplementary Figure 2A** for the 3D-CRT group, **Supplementary Figure 2B** for IMRT group, and **Supplementary Figure 2C** for 3D-IMRT–mixed group), the type of pathology (**Supplementary Figure 3A** for the ESCC group, and **Supplementary Figure 3B** for ESCC-mixed group), and the type of chemotherapy received (**Supplementary Figure 4A** for the CCRT group, **Supplementary Figure 4B** for CCRT+CT group, and **Supplementary Figure 4C** for CRT-mixed group), there were no substantial differences between them.

Overall, 15 studies (23–26, 28, 29, 31, 32, 34, 36–38, 41, 43, 44) analyzed the 2-year OS rates (**Figure 2B**), including 6 RCTs (23, 25, 29, 41, 43, 44) and 9 non-RCTs (24, 26, 28, 31, 32, 34, 36–38). No significant differences were observed between two groups (pooled RR, 0.99; 95% CI, 0.92–1.05; P = 0.70; high certainty) with no significant heterogeneity (P = 0.05; I^2 =^ 41%). As the study by Q. F. Li et al. (26) contributed significantly, we conducted a sensitivity analysis. Removing this did not change the 2-year OS rates. With regard to subgroup, the study type (**Supplementary Figure 5A** for RCTs group, and **Supplementary Figure 5B** for non-RCTs group), the type of radiotherapy (**Supplementary Figure 6A** for the 3D-CRT group, **Supplementary Figure 6B** for IMRT group, and **Supplementary Figure 6C** for 3D-IMRT–mixed group), the type of pathology (**Supplementary Figure 7A** for the ESCC group, and **Supplementary Figure 7B** for ESCC-mixed group), and the type of chemotherapy received (**Supplementary Figure 8A** for the CCRT group, **Supplementary Figure 8B** for CCRT+CT group, and **Supplementary Figure 8C** for CRT-mixed group), there were no substantial differences between them.

Notably, 20 studies (22–29, 31–34, 36–38, 40–44) analyzed the 3-year OS rates, including 6 RCTs (23, 25, 29, 41, 43, 44) and 14 non-RCTs (22, 24, 26–28, 31–34, 36–38, 40, 42). No significant differences were observed, although obvious heterogeneities were found among these studies (P < 0.00001; I^2^, 71%). As studies by Nakatani et al. (24), Q. F. Li et al. (26), and Zhu et al. (22) contributed significantly and weighted similarly, we excluded these three studies and found that the 3-year OS rates remained unchanged (pooled RR, 0.93; 95% CI, 0.85–1.02; P = 0.15; moderate certainty), with no significant heterogeneity (P = 0.15; I^2^, 31%; **Figure 2C**). With regard to subgroup, the study type (**Supplementary Figure 9A** for RCTs group, and **Supplementary Figure 9B** for non-RCTs group), the type of radiotherapy (**Supplementary Figure 10A** for the 3D-CRT group, **Supplementary Figure 10B** for IMRT group, and **Supplementary Figure 10C** for 3D-IMRT–mixed group), the type of pathology (**Supplementary Figure 11A** for the ESCC group, and **Supplementary Figure 11B** for ESCC-mixed group), and the type of chemotherapy received (**Supplementary Figure 12A** for the CCRT group, **Supplementary Figure 12B** for CCRT+CT group, and **Supplementary Figure 12C** for CRT-mixed group), there were no substantial differences between them.

In total, 13 studies (22–29, 31, 32, 36, 38, 41) analyzed 5-year OS rates, including 3 RCTs (23, 25, 29) and 10 non-RCTs (22, 24, 26–28, 31, 32, 36, 38, 41). No significant differences were observed, although obvious heterogeneities were found among these studies (P < 0.00001; I^2^, 74%). Subsequently, we excluded the study by Nakatani et al. (24) and found that the IFI group had a significant advantage over the ENI group in terms of 5-year OS rates (pooled RR, 0.78; 95% CI, 0.68–0.90; P = 0.0004; high certainty; **Figure 2D**), with no significant heterogeneity (P= 0.31; I^2^, 14%). With regard to subgroup, the type of radiotherapy, the IFI group had a significant advantage over the ENI group in the IMRT subgroup (**Supplementary Figure 13A**), whereas no differences were observed in the 3D-IMRT–mixed subgroup regarding the 5-year OS rate (**Supplementary Figure 13B**). With regard to subgroup, the type of pathology, the ESCC-mixed subgroup (**Supplementary Figure 14B**) showed a significant 5-year OS benefit but not the ESCC subgroup; however, obvious heterogeneities were found among these studies (P = 0.0003; I^2^, 73%). Therefore, we excluded the study by Nakatani et al. (24) and found that the IFI group had an advantage over the ENI group in 5-year OS rates, with no significant heterogeneity (**Supplementary Figure 14A**). Analysis of other subgroups was not conducted due to the lack of interested outcomes.

### Treatment-related Toxicities

**Figure 3:**
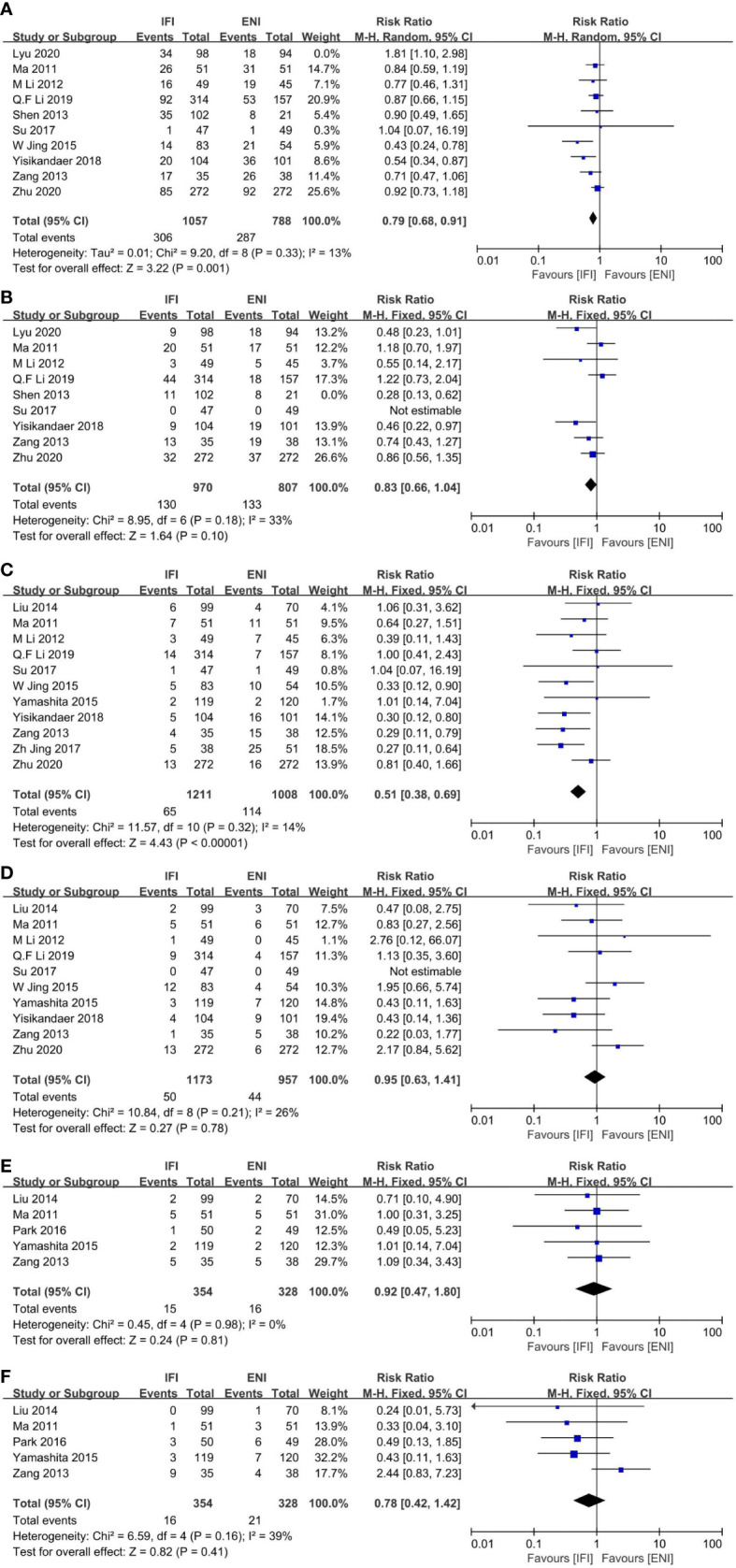
Forest plots of ≥ grade 2 acute esophagitis **(A)**, ≥ grade 2 acute pneumonitis **(B)**, ≥ grade 3 acute esophagitis **(C)**, grade 3 acute pneumonitis **(D)**, ≥ grade 3 late esophagitis **(E)** and ≥ grade 3 late pneumonitis **(F)**. IFI, involved field irradiation; ENI, elective nodal irradiation; M-H, Mantel-Haenszel; CI, confidence interval.

A total of 10 studies (22, 25, 26, 29, 31, 38, 41–44) analyzed the incidence of grade ≥2 acute esophagitis (AE), including 5 RCTs (25, 29, 41, 43, 44) and 5 non-RCTs (22, 26, 31, 38, 42). No significant differences were observed, although obvious heterogeneities were found among these studies (P = 0.03; I^2^, 53%). Therefore, we excluded the study by Lyu et al. (25) and found that IFI demonstrated a significant decrease in the incidence of grade ≥2 AE compared with ENI (pooled RR, 0.79; 95% CI, 0.68–0.91; P = 0.001; high certainty; **Figure 3A**), with no obvious heterogeneity (P = 0.33; I^2^, 13%). With regard to subgroup of the study type, no significant differences were observed in the RCTs; however, obvious heterogeneities were found among these studies. Therefore, we excluded the study by Lyu et al. (25) and found that the IFI group had an advantage over the ENI group in the RCTs, with no significant heterogeneity (**Supplementary Figure 15A**), similar to that of the non-RCT group (**Supplementary Figure 15B**). With regard to subgroup, the type of radiotherapy (**Supplementary Figure 16A** for the 3D-CRT group, and **Supplementary Figure 16B** for IMRT group), there were no substantial differences between them.

Nine studies (22, 25, 26, 29, 31, 41–44) analyzed grade ≥2 acute pneumonia (AP), including five RCTs (25, 29, 41, 43, 44) and four non-RCTs (22, 26, 31, 42). No significant differences were observed. Although obvious heterogeneities were found among these studies (P = 0.03; I^2^, 56%). Hence, we removed Shen et al. (42) and found that the results were unchanged (pooled RR, 0.83; 95% CI, 0.66–1.04; P = 0.10; high certainty; **Figure 3B**). With regard to subgroup of the study type, the IFI group showed a significant decrease in the incidence in RCTs (**Supplementary Figure 17A**), but not in non-RCTs (**Supplementary Figure 17B**). With regard to subgroup, the type of radiotherapy (**Supplementary Figure 18A** for the 3D-CRT group, and **Supplementary Figure 18B** for IMRT group), there were no substantial differences between them.

Overall, 11 studies (22, 26, 29, 31, 32, 37, 38, 40, 41, 43, 44) analyzed grade ≥3 acute esophagitis (AE) (**Figure 3C**), including 4 RCTs (29, 41, 43, 44) and 7 non-RCTs (22, 26, 31, 32, 37, 38, 40). IFI showed a significant reduction in the incidence compared with ENI (pooled RR, 0.51; 95% CI 0.38–0.69; P < 0.00001; high certainty) with no heterogeneity (P = 0.32; I^2^, 14%). With regard to subgroup, the type of radiotherapy, the IFI group demonstrated a significant reduction in the incidence in the 3D-CRT subgroup (**Supplementary Figure 20A**) and 3D-IMRT–mixed subgroup (**Supplementary Figure 20C**), whereas there was no difference in the IMRT subgroup (**Supplementary Figure 20B**). With regard to subgroup, the study type (**Supplementary Figure 19A** for RCTs group, and **Supplementary Figure 19B** for non-RCTs group), and the type of pathology (**Supplementary Figure 21A** for the ESCC group, and **Supplementary Figure 21B** for ESCC-mixed group), there were no substantial differences between them.

Ten studies (22, 26, 29, 31, 37, 38, 40, 41, 43, 44) analyzed grade ≥3 acute pneumonia (AP) (**Figure 3D**), including four RCTs (29, 41, 43, 44) and six non-RCTs (22, 26, 31, 37, 38, 40). No significant differences were observed between two groups (pooled RR, 0.95; 95% CI, 0.63–1.41; P = 0.78; high certainty) with no heterogeneity (P = 0.21; I^2^, 26%). With regard to subgroup, the study type (**Supplementary Figure 22A** for RCTs group, and **Supplementary Figure 22B** for non-RCTs group), and the type of radiotherapy (**Supplementary Figure 23A** for the 3D-CRT group, and **Supplementary Figure 23B** for IMRT group), the type of pathology (**Supplementary Figure 24A** for the ESCC group, and **Supplementary Figure 24B** for ESCC-mixed group), there were no substantial differences between them.

Five studies (33, 37, 40, 41, 44) analyzed grade ≥3 late esophagitis (LE) (**Figure 3E**), including two RCTs (41, 44) and three non-RCTs (33, 37, 40). No differences were observed (pooled RR, 0.92; 95% CI, 0.47–1.80; P= 0.81; high certainty). Furthermore, five studies (33, 37, 40, 41, 44) analyzed grade ≥3 late pneumonia (LP) (**Figure 3F**), including two RCTs (41, 44) and three non-RCTs (33, 37, 40), and no differences were observed (pooled RR, 0.78; 95% CI, 0.42–1.42; P = 0.41; high certainty).

### PFS and local control rates

**Figure 4:**
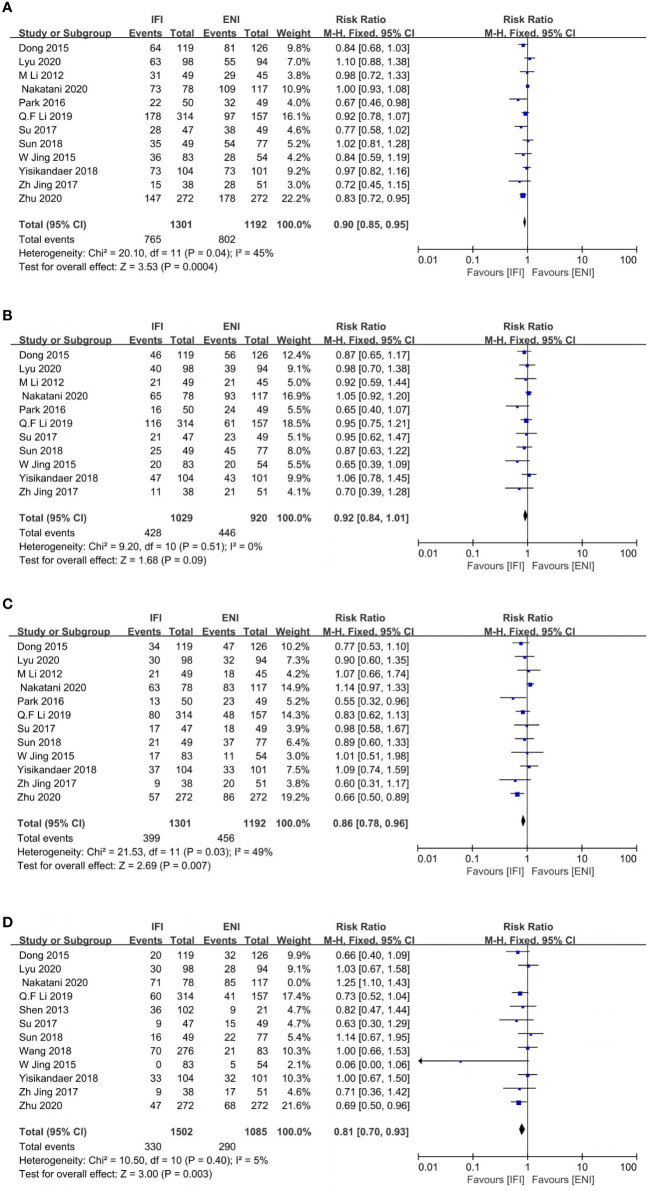
Forest plot of 1- **(A)**, 2- **(B)**, 3- **(C)** and 5-years **(D)** progression free survival rate. IFI, involved field irradiation; ENI, elective nodal irradiation; M-H, Mantel-Haenszel; CI, confidence interval.

Totally, 12 (22, 24–26, 28, 29, 31–33, 36, 38, 43), 11 (24–26, 28, 29, 31–33, 36, 38, 43), 12 (22, 24–26, 28, 29, 31–33, 36, 38, 43), and 12 (22, 24–29, 31, 32, 36, 38, 42) studies were to analyze 1-, 2-, 3-, and 5-years PFS rates. IFI group had an advantage over ENI at 1-, and 3-year PFS rates (1-year PFS: pooled RR, 0.90; 95% CI, 0.85–0.95; P = 0.0004; high certainty; **Figure 4A**; 3-year PFS: pooled RR, 0.86; 95% CI, 0.78–0.96; P = 0.007; moderate certainty; **Figure 4C**). No differences were observed at 2- and 5-year PFS rates (2-year PFS: pooled RR, 0.92; 95% CI, 0.84–1.01; P = 0.09; high certainty; **Figure 4B**). However, obvious heterogeneities were found among these studies at 5-year PFS rate (P < 0.0001; I^2^, 71%). Subsequently, we excluded the study by Nakatani et al. (24) and re-evaluated the 5-year PFS rate. We showed that the IFI group had an advantage over the ENI group (pooled RR, 0.81; 95% CI, 0.70–0.93; P = 0.003; high certainty; **Figure 4D**) with no heterogeneity (P= 0.40; I^2^, 5%).

**Figure 5:**
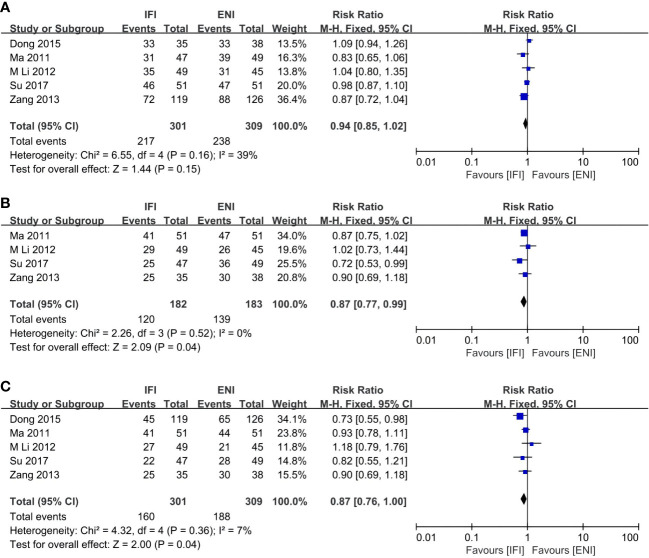
Forest plots of 1- **(A)**, 2- **(B)** and 3-years **(C)** local control rate. IFI, involved field irradiation; ENI, elective nodal irradiation; M-H, Mantel-Haenszel; CI, confidence interval.

Five (31, 36, 41, 43, 44), four (31, 41, 43, 44), and five (31, 36, 41, 43, 44) studies analyzed the 1-, 2-, and 3-year LCRs. The IFI group had a significant advantage over the ENI group at 2- and 3-year LCRs (2-year LCR: pooled RR, 0.87; 95% CI, 0.77–0.99; P = 0.04; moderate certainty; **Figure 5B**; 3-year LCR: pooled RR, 0.87; 95% CI, 0.76–1.00; P = 0.04; high certainty; **Figure 5C**), while no differences at 1-year LCR (pooled RR, 0.94; 95% CI, 0.85–1.02; P = 0.15; high certainty; **Figure 5A**).

### Quality assessed

Details of the evaluation for RCTS and non-RCTs are provided in **Supplementary Tables 5** and **6**. The Cochrane Risk of Bias tool showed that equality was high in the included studies. In the included RCTs, only one RCT had a bias in blinding participants and health care providers, and no other bias was observed. The AHRQ Standards were all good. The NOS scores of non-RCTs were between 7 and 9. The average score was 8.3. All 17 studies had selection scores of 4. Comparability scores were rated as 2 and 1 in 58.8% (10/17) and 41.2% (7/17) of the studies, respectively. The outcome scores were rated as 3 and 2 in 70.6% (12/17) and 29.4% (5/17) of the studies, respectively.

As a result, the overall quality is excellent. **Supplementary Table 7** summarizes all outcomes with GRADE quality evaluation. Funnel plots for outcomes were evaluated in **Supplementary Figures 25**–**33**.

## Discussion

As far as we know, this is the first systematic review and meta-analysis that included the highest number of patients and RCTs. In this study including 4120 patients, IFI significantly improved OS at 5 years (RR, 0.78) but not at 1, 2, and 3 years with high-to-moderate certainty of evidence. For subgroup analyses performed based on the study type, pathology, radiotherapy, and chemotherapy, the findings observed in each subgroup were consistent with the general finding that OS unchanged at 1, 2, and 3 years. However, regarding the 5-year OS rate, the IMRT subgroup demonstrated improvement, whereas no difference was observed in the 3D-IMRT–mixed subgroup. Regarding treatment-related adverse events, IFI was associated with a significant improvement in grade ≥2 AE (RR, 0.79), similar to the findings observed in the subgroup analyses. Furthermore, there was no differences in the incidence of grade ≥2 AP, and only RCTs reported that the IFI group demonstrated an improvement in the grade ≥2 AP (RR, 0.69). The IFI group demonstrated significant improvements in grade ≥3 AE events compared with the ENI group (RR, 0.51), while no difference was observed in the IMRT subgroup compared with the 3D-CRT and 3D-IMRT–mixed subgroups. Additionally, no differences were observed in the incidence of grade ≥3 AP (RR, 0.95), grade ≥3 LE (RR, 0.91) and grade ≥3 LP (RR, 0.89) between two groups. Regarding PFS, IFI had an improvement in 1-, 3-, and 5-year PFS rates (RR, 0.90, 0.86, and 0.81, respectively) but not in 2-year PFS rates (RR, 0.92; 95% CI, 0.84–1.01). IFI had an improvement in the 2- and 3-year LCR (RR, 0.87 and 0.87, respectively) but not in the 1-year LCR (RR, 0.94; 95% CI, 0.85–1.02).

Although this is the largest meta-analysis to explored the role of IFI and ENI in definitive radiotherapy or chemoradiotherapy for esophageal cancer, previous studies have made a few attempts. A meta-analysis conducted by H.P. Zhu er al (45). demonstrated no difference in the OS rates and the incidence of AE and LP between those who underwent IFI and ENI; however, this meta-analysis only included seven articles in which there were two abstracts submitted to conferences without full texts and only one RCT, and the study only compared the 1-, 2-, and 3-year OS rates and the incidence of grade ≥3 AE/AP and grade ≥3 LE, which showed the same conclusions as our study. Our systematic review also demonstrated that IFI had an improvement in the 5-year OS rate and grade in ≥2 AE compared with ENI; however, no difference in the incidence of grade ≥2 AP was observed. Although we showed that no differences were observed in the incidence of grade ≥3 LP, this finding differed from that reported by H.P. Zhu er al (45).; the difference can be explained that we included one more RCT, which provided a higher level of evidence. For subgroup analyses performed by the study type, there was no difference in the 1-, 2-, 3-year OS rates and grade ≥3 AE/AP events between RCTs and non-RCTs, similarly to H.P. Zhu et al. (45). While most studies demonstrated no differences in the survival benefit between two groups, our study challenges these results; although we found no difference in the short-term survival, IFI demonstrated improved long-term survival compared with ENI. Two ongoing randomized controlled clinical phase III trials, the CSWOG 003 trial by T. Li et al. (46) and the NROG 001 trial by B. Li et al. (47), showed in the interim analysis that there was no difference in the 1- and 2-year OS rates, similar to ours. In 2018, S. Tsuruoka et al. (48) reported that there was no difference in the 3-year OS, similarly our study; although the patients included in their study all had esophageal cancer in the thoracic region. As shown in the subgroup analyses, the type of radiotherapy, chemotherapy, and pathology were not associated with the 1-, 2-, and 3-year OS. Furthermore, the clinical outcomes of RCTs were consistent with those of non-RCTs. Regarding the analysis of 5-year OS rates in the subgroups, the IFI group demonstrated significant improvement compared with the ENI group in the IMRT subgroup, while there were no differences in the 3D-IMRT–mixed subgroup. Owing to the missing data regarding the 5-year OS rates in the 3D-CRT subgroup, we performed an indirect comparison between IMRT and 3D-CRT. Differences in survival were observed, which may be attributed to technical differences, indicating that, in the era of 3D-CRT, there is no difference in 5-year OS rates following IFI and ENI. Although the popularity of IMRT is increasing, IFI offers a better improvement in 5-year OS rates than ENI, which may be explained by the fact that IMRT can increase the therapeutic effect and reduce the size of the radiotherapy target compared with 3D-CRT. Moreover, treatment-related adverse events associated with 3D-CRT may show no differences in the 5-year OS between two groups. The more favorable results of IMRT compared with the old 3D technique confirmed the superiority of IMRT also for esophageal cancer. The outcomes by the type of pathology were consistent with the overall conclusion.

Regarding treatment-related adverse events, although E. Jean-Mary et al. (19) reported that it was feasible for ENI when the surrounding tissues were not in high doses, they only reported this finding from the viewpoint of the target volume and the exposure dose to the organ at risk without considering the actual clinical outcomes. Our study reported that IFI was associated with a significant improvement in grade ≥2 AE, similarly to T. Li et al. (46), and the findings of the subgroup analyses were also consistent with this conclusion. There was no difference in the incidence of grade ≥2 AP between two groups. Only RCTs reported that IFI showed a significant improvement in grade ≥2 AP, which varies from the overall conclusion and those derived from the other subgroup analyses, which may be explained by the fact that a retrospective analysis is more accurate than an RCT for evaluating the adverse events. Therefore, more prospective studies are needed to validate this finding. In our study, IFI showed significant reductions in the incidence of grade ≥3 AE compared with ENI, which is similar to that reported in most studies (45, 49, 50). Compared with the 3D-CRT and 3D-IMRT–mixed subgroups, there were no differences in the incidence of grade ≥3 AE in the IMRT subgroup, which could be explained by the fact that IMRT may reduce the difference in the incidence rates of grade ≥3 AE between two groups. Another challenging finding from our systematic review was that there were no differences in the incidence of grade ≥3 AP between them and the other subgroups, similarly to Zhu et al. (45) and different from Cheng et al. (49) and Jing et al. (50). The databases these two authors searched included the China National Knowledge Infrastructure (CNKI) and Chinese Biomedical Literature Database, without including the Web of Science database, which differed from the databases included in the present study. Moreover, studies by Jing et al. (50) and Cheng et al. (49) were performed before 2015 except for one study by Cheng et al. Most of their treatment regimens included radiotherapy alone, in which the patient received a larger dose of radiation, which resulted in difference in the incidence rates of grade in ≥3 AP between those undergoing IFI and ENI. A few studies reported on late treatment side effects. Our systematic review showed that there were no differences in the incidence of grade ≥3 LE, which is consistent with Zhu et al. (45). Zhu et al. (45) reported that IFI demenstrated a decrease in the incidence of grade ≥3 LP compared with ENI, this finding differed from that reported in our study where we reported no difference. Zhu et al. (45) included four studies to analyze the incidence of grade ≥3 LP; in contrast, we included five studies, four of which were the same as Zhu et al. (45). We included one other study by Zang et al. (41), which reported an RR value of 2.44 (95% CI, 0.83–7.23) with very high upper limit value. As both Zhu et al. and the present study included only a few studies, conclusions regarding the incidence of grade ≥3 LP could not be provided.

As this review included retrospective studies, it was difficult to accurately determine the time of tumor recurrence; therefore, we evaluated PFS and LCR as secondary outcomes, although the secondary outcomes were not analyzed in the subgroup. We reported that IFI had an improvement in PFS at 1, 3, and 5 years, but not at 2 years, and the pooled RR for the 2-year PFS was 0.92 (95% CI, 0.84–1.01), where the upper line of the CI of the RR value is only slightly above 1.00. Furthermore, we showed that IFI had an improvement in the LCR at 2 and 3 years, but not at 1 year (RR, 0.94; 95% CI 0.85–1.02), where the upper line of the CI of the RR value is also slightly above 1.00. The reason why we could not draw conclusions regarding the time of tumor recurrence may be attributed to the bias and errors observed in retrospective studies, which is also a limitation of the present study. Additionally, the time of tumor recurrence and control differed in previous studies. Zhou et al. (51) reported that no differences were reported in the 1- and 2-year LCRs (75% and 57%; 72% and 45%, respectively; χ^2^, 0.79; P = 0.376) in those undergoing IFI and ENI, similar to those reported by Li et al. (52) (1- and 2-year LCRs—66% and 48% and 68% and 49%, respectively; χ^2^, 0.56; P = 0.78). However, Zhu et al. (53) reported that ENI showed a significant improvement in LCRs at 1, 3, and 5 years compared with IFI (70.5% and 53.3%; 51.7% and 63.0%; 39.1% and 27.2%, respectively; χ^2^, 6.22; P = 0.013). Therefore, the advantages and disadvantages of IFI and ENI in tumor control and recurrence cannot be concluded.

There were also some limitations to this meta-analysis. First, most publications in this study were retrospective studies, with only six RCTs. Although subgroup analyses were performed based on the study type, the results of 1-, 2-, and 3-year OS rates and the incidence of grade 2 AE and grade ≥3 AE/AP did not differ from the overall conclusion; however, differences in the incidence of grade ≥2 AP were observed. Furthermore, very few RCTs reported 5-year OS rates and grade ≥3 LE/LP events; hence, we could not fully analyze the impact on these results because the evidence for these results was limited. Though the OS is necessary for efficacy evaluation, PFS and LCRs are also needed to refine the effectiveness of treatment (54, 55). Nevertheless, IFI demonstrated a meaningful improvement in OS at 5 years but not at 1, 2, and 3 years; this is an important discovery that should be further investigated. Treatment-related adverse events related to radiotherapy must be evaluated in future prospective studies to consolidate the evidence. Second, only a few studies investigated a single subgroup included in this study and reported relevant outcomes. Therefore, in the subgroup analysis, in addition to including relevant subgroups, mixed groups were also included for indirect comparison in the present study. Third, in conducting this study, there were five head-to-head ongoing RCTs investigating IFI and ENI, none of which have reported the final results; only the interim results were published. Therefore, we excluded these studies. Forth, due to the inability to obtain data separately and the lack of comprehensive reporting in the included literature, we also included a small proportion of patients with stage IV, which would also have a small impact on this study. Fifth, although the patients included in this article were by far the largest under the relevant topic, the total number of patients was still not very large. Moreover, the articles included in this study were all from Asia, on the one hand, the incidence of esophageal cancer was much higher in Asia than in other regions, and on the other hand, when we were screening and including the articles, we only included head-to-head articles of IFI and ENI to ensure the high quality of the study. Many articles from other regions were excluded by us in this process. In addition to this, we only included articles that provided full texts in order to ensure the comprehensiveness of the study. Therefore, conference abstracts without full texts presented at academic conferences such as ASCO and ESMO, for example, were not included in this study.

## Conclusions

To sum up, this meta-analysis demonstrated that IFI had improvements in the 5-year OS rate, but not at 1, 2, and 3 years, compared with ENI. We showed that the addition of IMRT to IFI improves the 5-year OS, whereas the same cannot be observed by the addition of 3D-CRT to IFI and ENI. IFI reduced the incidence of grade ≥2 and ≥3 AE compared with ENI, while IMRT showed no difference in the incidence of grade ≥3 AE. Furthermore, IFI and ENI showed no difference in the incidence of grades ≥3 AP, LE, and LP. There is a limited amount of data on which to draw conclusions regarding PFS and LCRs. Our systematic review presents interesting information in terms of the potential survival improvements imparted by IFI in esophageal cancer patients receiving radical radiotherapy or CRT. Long-term follow-up prospective study should be performed to further validate IFI implementation.

## Data availability statement

The original contributions presented in the study are included in the article/**Supplementary Material**. Further inquiries can be directed to the corresponding author.

## Author contributions

All authors read and approved the final manuscript prior to submission. WS is responsible for the conception and design of the study. HW is responsible for analyzing and interpreting data, drafting the article, and revising it. CS and XZ are responsible for the acquisition of data. WD is responsible for data checks. All authors contributed to the article and approved the submitted version.

## Acknowledgments

We would like to express our sincere thanks to Department of Radiation Oncology, Fourth Hospital of Hebei Medical University. We thank Jingyuan Wen and Luanying Wu for their assistance during this work.

## Conflict of interest

The authors declare that the research was conducted in the absence of any commercial or financial relationships that could be construed as a potential conflict of interest.

## Publisher’s note

All claims expressed in this article are solely those of the authors and do not necessarily represent those of their affiliated organizations, or those of the publisher, the editors and the reviewers. Any product that may be evaluated in this article, or claim that may be made by its manufacturer, is not guaranteed or endorsed by the publisher.

## Abbreviations

IFI, involved field irradiation; ENI, elective nodal irradiation; GTV, gross tumor volume; CTV, clinical target volume; PTV, planned target volume; PRISMA, Preferred Reporting Items for Systematic Reviews and Meta-Analyses; NOS, Newcastle-Ottawa Scale; AHRQ, Agency for Healthcare Research and Quality; GRADE, Grading of Recommendations Assessment, Development, and Evaluation; CT, chemotherapy; CRT, chemoradiotherapy; CCRT, concurrent chemoradiotherapy; 3D-CRT, three-dimensional conformal radiotherapy; IMRT, intensity-modulated radiotherapy; ESCC, esophageal squamous cell carcinoma; RCT, randomized controlled trial; RR, risk ratio; CI, confidence interval; OS, overall survival; PFS, progression-free survival; LCR, local control rate; AE, acute esophagitis; AP, acute pneumonia; LE, late esophagitis; LP, late pneumonia.
