# Supplementary material for: The role of involved field irradiation versus elective nodal irradiation in definitive radiotherapy or chemoradiotherapy for esophageal cancer- a systematic review and meta-analysis
Source: Front Oncol. 2022 Nov 2;12:1034656. doi: 10.3389/fonc.2022.1034656 (PMC9666894; doi:10.3389/fonc.2022.1034656)
Supplement: Supplementary file 1 [file DataSheet_1.zip › supplementary materials/Supplementary Table/Supplementary Table 1. Search Strategy.docx]

| 1 | "Cancer of Esophagus"[Title/Abstract] |
| --- | --- |
| 2 | "Cancer of the Esophagus"[Title/Abstract] |
| 3 | "Esophageal Cancer"[Title/Abstract] |
| 4 | "Esophagus Cancer"[Title/Abstract] |
| 5 | "Esophagus Neoplasm"[Title/Abstract] |
| 6 | "Carcinoma of Esophagus"[Title/Abstract] |
| 7 | "Esophageal squamous cell carcinoma"[Title/Abstract] |
| 8 | "Esophageal squamous carcinoma"[Title/Abstract] |
| 9 | ESCC[Title/Abstract] |
| 10 | OR/1-9 |
| 11 | Radiotherapy[Title/Abstract] |
| 12 | Chemoradiation[Title/Abstract] |
| 13 | Irradiation[Title/Abstract] |
| 14 | "Radiation therapy"[Title/Abstract] |
| 15 | OR/11-14 |
| 16 | "elective lymph node irradiation"[Title/Abstract] |
| 17 | "selective lymph node irradiation"[Title/Abstract] |
| 18 | "elective nodal irradiation"[Title/Abstract] |
| 19 | ENI[Title/Abstract] |
| 20 | ENRA[Title/Abstract] |
| 21 | "involved field irradiation"[Title/Abstract] |
| 22 | IFI[Title/Abstract] |
| 23 | IFRA[Title/Abstract] |
| 24 | OR/16-23 |
| 25 | 10 AND 15 AND 24 |
| 26 | Remove duplicates from 25 |

**Supplementary Table 1**. Search Strategy
