# Supplementary material for: The role of involved field irradiation versus elective nodal irradiation in definitive radiotherapy or chemoradiotherapy for esophageal cancer- a systematic review and meta-analysis
Source: Front Oncol. 2022 Nov 2;12:1034656. doi: 10.3389/fonc.2022.1034656 (PMC9666894; doi:10.3389/fonc.2022.1034656)
Supplement: Supplementary file 1 [file DataSheet_1.zip › supplementary materials/Supplementary Table/Supplementary Table. 2 Summary of Treatment.docx]

**Supplementary Table. 2** Summary of Treatment

| **Study** | **Radiotherapy target size** | **N analyzed** | **Irradiation range** | **RT dose** | **RT technology** | **Type of treatment** | **Chemotherapy drugs** |
| --- | --- | --- | --- | --- | --- | --- | --- |
| Zhu,  2020 | IFI | 272 | - | PTV/PTVnd: 56-66 Gy, 1.8-2.0 Gy/ fraction | IMRT | RT±CT | - |
|  | ENI | 272 | - | PTV/PTVnd: 56-66 Gy, 1.8-2.0 Gy/ fraction  PTV1: 46-52 Gy, 1.8-2.0 Gy/ fraction | IMRT | RT±CT | - |
| Xie,  2020 | IFI | 88 | The gross tumour volume (GTV) was defined as the primary tumour, and any enlarged regional lymph nodes were indicated by transoesophageal ultrasound, oesophagram, CT scan and PET/CT (when indicated). The CTV consisted of CTV1 and CTV2. CTV1 was  defined as the primary tumour plus the superior and inferior 4-cm margins, radial 1-cm margin and enlarged lymph nodes. After 40 Gy of radiotherapy, CTV2 (boost CTV) was defined as GTV plus the superior and inferior 2-cm margins and radial 1-cm margins. Initially, 40 Gy was given to CTV1, and a boost dose of 20 Gy was then delivered to CTV2. | 60Gy | - | CCRT | Tntravenous paclitaxel (135 mg/m2, day 1) and cisplatin (20 mg/m2, days 1–3) every 4 weeks for two cycles. |
|  | ENI | 88 | The initial CTV1 included the whole oesophagus plus regional lymph nodes. According to the location of the tumour, the regional lymph nodes were prophylactically irradiated. | 60Gy | - | CCRT | The same as IFI. |
| Nakatani,  2020 | IFI | 78 | The clinical target volume (CTV) included the primary tumor plus a 2-cm craniocaudal margin. The planning target volume (PTV) was defined as CTV plus a 1 to 1.5-cm margin in the craniocaudal direction and 1-cm margin in the lateral direction. | 60 Gy | 2D-RT  3D-CRT | RT + CT | 5-FU and cisplatin (700 mg/m2, days 1–4, 29–32, and 70 mg/m2, day 1, 29). |
|  | ENI | 117 | The CTV was designed to cover mediastinal and upper perigastric lymph nodes for all cases, and additionally include bilateral supraclavicular fossae for the upper thoracic primary tumor and celiac axis lymph nodes for the lower thoracic primary tumor. | 60 Gy | 2D-RT  3D-CRT | RT + CT | The same as IFI. |
| Lyu,  2020 | IFI | 98 | The GTV included the primary cancer (total GTV [GTVt]) and metastatic lymph nodes (nodal GTV [GTVn]). The definition of GTV was a primary tumor detected by barium esophagography, endoscopy, or CT. GTV also included positive [18F]-fluorodeoxyglucose positron emission tomography (except for physiological build-up) and all lymph nodes with short axis diameters greater or equal to 1.0 cm.Both Nodal CTV (CTVn) and CTVt were used to establish CTV. CTVt definition encompassed the area of GTVt in addition to 0.8-1.0 cm on the left and right as well as 3.0 cm above and below the primary tumor. IFI CTVn only includes the area of lymph nodes station 4. 1.0 and 5 mm longitudinal and radial margins, respectively, were applied to the CTV to generate PTV. | GTV: 60-66 Gy, 1.8-2Gy/fractions  CTV: 50-54 Gy, 1.8-2Gy/ fraction | IGRT | CCRT ± CT | 2-4 cycles of docetaxel (first day dose = 75.0 mg/m2) combined with cisplatin (doses on days 1-3 = 25.0 mg/m2) every 21-28 days. After CCRT, additional 1-2 rounds of ancillary chemotherapy were given to patients with adequate bone marrow function and a good performance status. |
|  | ENI | 94 | The GTV, CTVt and PTV were the same as the IFI group. ENI CTVn encompassed both clinically involved or uninvolved lymph node regions or stations, respectively, in accordance with the site of the primary tumor (lower, middle, and upper thoracic ESCC: station numbers for lymph node being 4/5/7/8/9/16/17, 2/4/5/7/8/9, and 1/2/4/5/7, respectively). | GTV: 60-66 Gy, 1.8-2Gy/fractions  CTV: 50-54 Gy, 1.8-2Gy/ fraction | IGRT | CCRT ± CT | The same as IFI. |
| Q.F Li,  2019 | IFI | 314 | The GTV was visualized on computed tomography (CT) and X-ray and/or endoscopic extension. All LNs with a diameter of at least 1 cm in the short axis in CT or that were positive by 18 fluorodeoxyglucose positron emission tomography were defined as GTV-LNs. The CTV was generated by using 0.5 to 0.8 cm radial margin and 2 to 3 cm longitudinal margins to the GTV-primary, and CTV-LNs by using 0.5 cm margin for the GTV-LNs. The PTV was generated by applying a 5 to 10mm margin to the CTV, and PTV-LNs by using 0.5 to 0.8 cm margin for CTV-LNs. | PTV/PTV-LN: 56-66 Gy, 1.8-2.0/ fraction | IMRT | RT±CT | combination with 5-fluorouracil and taxane, or with platinum-based compounds. |
|  | ENI | 157 | Patients received irradiation in the same PTV/PTV-LNs as in the IFI field. CTV1: for the supraclavicular area, treatment of higher echelon cervical nodes was considered. For the proximal third of the esophagus, the paraesophageal LNs and the supraclavicular area were treated. For middle lesions, the paraesophageal LNs were treated. For the distal and the gastroesophageal junction, the lesser curvature, celiac axis, and paraesophageal LNs were treated. PTV1: add margins of 0.5 to 1.0 cm to CTV1. | PTV/PTV-LN: 56-66 Gy, 1.8-2.0/ fraction  PTV1: 50-54 Gy, 1.8-2.0 Gy/ fraction | IMRT | RT±CT | The same as IFI. |
| Wang,  2018 | IFI | 276 | - | 50-70 Gy/ 25-35 fraction | 3D-CRT  IMRT | RT | - |
|  | ENI | 83 | - | 50-70 Gy/ 25-35 fraction  45-50 Gy/ 23-25 fraction | 3D-CRT  IMRT | RT | - |
| Sun,  2018 | IFI | 49 | The GTV included a field containing the primary tumour, plus a 3.0 cm margin superior and inferior and a 0.8–1.0 cm radial margin the esophageal CTV. The PTV was defined as the esophageal CTV plus a 0.5–1.0 cm margin. | 50-64 Gy, 1.8-2.0 Gy/ fraction | 3D-CRT  IMRT | CCRT | 5-fluorouracil– cisplatin, docetaxel– cisplatin, paclitaxel–carboplatin, and single-agent paclitaxel, cisplatin, or S-1. |
|  | ENI | 77 | The GTV was the same as the IFI group. The nodal CTV included the adjacent regional lymphatics depending on the location of the primary tumour. The PTV was defined as the esophageal CTV plus nodal CTV plus a margin of 0.5–1.0 cm. | 40-50 Gy, 10–20 Gy booster dose to the primary tumour only, 1.8-2.0 Gy/ fraction | 3D-CRT  IMRT | CCRT | The same as IFI. |
| Yisikandaer,  2018 | IFI | 104 | GTV is delineated as the tissue with esophageal wall thickness t>0.5cm displayed on the CT image, and the results of barium esophagography and esophagoscopy are referred to. CTVt is 0.5cm from the front, back, left and right sides of the GTV. The upper and lower directions were extended 3.0 cm, and the PTVt was appropriately modified according to the anatomical boundary to be CTVt, which was expanded by 1 cm up and down, and 0.5 cm in the periphery. GTVln was delineated as a clinically confirmed metastatic lymph node.CTV-ln: Only the lymph node drainage area where the positive lymph node is located is included. The lymph node drainage area was evenly expanded by 0.5 to 1.0 cm as PCTV-ln. | GTV/GTV-ln: 60-66 Gy, 1.8-2.0/ fraction  PTV-t/PCTV-ln: 50-54 Gy, 1.8-2.0/ fraction | IMRT | CCRT ± CT | Receive at least 1 cycle of concurrent chemotherapy with docetaxel and cisplatin. . Docetaxel 75mg/m2, intravenous drip, d1; cisplatin 25mg/m2, intravenous drip, d1-3; 21d is a cycle. After radiotherapy, 1-2 cycles of adjuvant chemotherapy were given according to the patient's physical condition. |
|  | ENI | 101 | CTV-ln: In addition to the lymphatic drainage area where the positive lymph nodes are located, upper thoracic esophageal cancer should also include bilateral supraclavicular, 2, 4, 5, and 7 lymphatic drainage areas; midthoracic esophageal cancer includes 2, 4, 5, and 7 , 8, 9 lymph node drainage area: In the lower thoracic segment, it includes areas 2, 4, 5, 7, 8, and 9, next to the cardia, the left stomach and the lymphatic drainage area next to the celiac trunk. The lymph node drainage area is evenly expanded by 0.5 to 1.0 cm as PCTV-In. | GTV/GTV-ln: 60-66 Gy, 1.8-2.0/ fraction  PTV-t/PCTV-ln: 50-54 Gy, 1.8-2.0/ fraction | IMRT | CCRT ± CT | The same as IFI. |
| Zhao,  2017 | IFI | 40 | Gross tumor volume (GTV) was defined as the primary tumor (GTVt) and involved lymph nodes (GTVnd) based on all available information deriving from barium swallow, laryngoscopy, contrast enhanced neck/chest computed tomography scan (CT), endoscopy/EUS and 18FDG-positron emission tomography (PET)-CT.The clinical tumor volume (CTV) comprised the GTVt plus additional 3cm cranial-caudal and 0.7-1cm radial margin expansions, respectively, as well as involved or elective nodal regions, which included supraclavicular fossa and upper mediastinal areas. the planning target volume (PTV) was created with a 0.5-1.0 cm margin from GTV and CTV respectively, which were named as PGTV (PGTVt and PGTVnd) and PCTV (PCTVt and PCTVnd). | GTV: 50-70 Gy/ 25-35 fraction  CTV: 50-54 Gy | 3D-CRT  IMRT  VMAT | RT±CT | combination of cisplatin and 5-fluorouracil, oral capecitabine or S1 alone. |
|  | ENI | 46 | The same as IFI | GTV: 50-70 Gy/ 25-35 fraction  CTV: 50-54 Gy | 3D-CRT  IMRT  VMAT | RT±CT | The same as IFI. |
| Su,  2017 | IFI | 47 | The gross tumour volume (GTV) contained the primary oesophageal tumour. The clinical target volume (CTV) was obtained by adding a 0.5–1.0 cm margin in axial direction and a 1.5–2.0 cm margin in cranial–caudal direction to the primary tumour. The planning target volume (PTV) was generated by expanding the CTV with 0.5–0.8 cm margin. The gross tumour volume of lymph node (GTVnd) included the pathologic lymph nodes. The clinical target volume of lymph node (CTVnd) was obtained by adding a 0.5 cm margin around the pathologic lymph nodes. The planning target volume of lymph node (PTVnd) was generated by expanding the CTVnd with 0.5–0.8 cm margin. | PTV/PTV-nd: 56-66 Gy/ fraction | 3D-CRT  IMRT | RT±CT | 5-fluorouracil and cisplatin. |
|  | ENI | 49 | The clinical target volume of elective nodal irradiation was defined as CTV1. When the primary tumour was located at cervical or upper thoracic oesophagus, CTV1 contained bilateral supraclavicular, adjacent oesophagus, and regions 2, 4, 5 and 7. When the primary lesion was located at middle thoracic oesophagus, CTV1 contained lymphatic drainage area of adjacent oesophagus, regions 2, 4, 5 and 7. For the lower thoracic oesophageal carcinoma, CTV1 contained adjacent oesophagus, region 4, 5, and 7, left gastric and paracardial lymph drainage area. The planning target volume of elective nodal irradiation was defined as PTV1, which was obtained by expanding the CTV1 with 0.5–1.0 cm margin. | PTV/PTV-nd: 56-66 Gy, 1.8-2.0 Gy fraction  PTV1: 46-52.2 Gy, 1.8-2.0 Gy/ fraction | 3D-CRT  IMRT | RT±CT | The same as IFI. |
| Zh Jing,  2017 | IFI | 38 | The gross tumor volume (GTV) was defined as the primary tumor and any enlarged regional lymph nodes indicated by the transesophageal ultrasound, esophagram, CT scan and PET/CT (when available). The clinical target volume (CTV) consisted of CTV1 and CTV2. CTV1 was defined as GTV plus superior–inferior 4-cm margin and radial 1-cm margin. After 40 Gy of radiotherapy, CTV2 (boost CTV) was defined as GTV plus the superior–inferior 2-cm and radial 1-cm margin. In patients receiving CFI, CTV1 was defined as GTV plus the superior–inferior 4-cm margin and radial 1-cm margin. After 40 Gy of radiotherapy, CTV2 (boost CTV) was defined as GTV plus the superior–inferior 2-cm and radial 1-cm margin. | 40 Gy (CTV1) + 20 Gy (CTV2) | 3D-CRT  IMRT | CCRT | Paclitaxel (135 mg/m2, day 1) and cisplatin (20 mg/m2, days 1–3) or oxaliplatin (130 mg/m2, day 2) every 4 weeks for two cycles. |
|  | ENI | 51 | (CTV1) included whole esophagus plus regional lymph nodes. According to the location of the tumor, the regional lymph nodes were prophylactic irradiated. For the cervical esophageal tumor, the level II/III lymph nodes in the neck, supraclavicular, paratracheal, posterior mediastinal, aortopulmonary, subcarinal, paraesophageal, pulmonary ligament, diaphragmatic and paracardial lymph nodes were included. For the upper and middle thoracic tumor, supraclavicular, paratracheal, posterior mediastinal, aortopulmonary, subcarinal, paraesophageal, pulmonary ligament,  diaphragmatic, paracardial and left gastric lymph nodes were included. For the lower thoracic tumor, paratracheal, posterior mediastinal, aortopulmonary, subcarinal, paraesophageal, pulmonary ligament, diaphragmatic, paracardial, left gastric and celiac lymph nodes were included. | 40 Gy (CTV1) + 20 Gy (CTV2) | 3D-CRT  IMRT | CCRT | The same as IFI. |
| Park,  2016 | IFI | 50 | The GTV esophagus was defined as any visible lesion from endoscopic ultrasonography, diagnostic CT images and FDG-PET/CT. The GTV node was defined as positive when a discernible increase in FDG uptake (max SUV >2.5) was observed on FDG-PET without inflammatory signs, such as calcification. In short, GTV included primary lesion and metastatic lymph nodes. The margin for the clinical target volume (CTV) from GTV esophagus was 3 cm cranially and caudally with a 1-cm radial margin and GTV node plus 0.5-cm margin. The CTV was generated by using 1-cm radial margin and 3-cm longitudinal margins to the GTV esophagus and by using 0.5-cm margin for the GTV node. Any lymph node areas were not electively irradiated in this treatment planning. | 54 Gy, 1.8-2.0/ fraction | 3D-CRT | CCRT | Two cycles of chemotherapy were delivered during radiotherapy, starting on days 1–22, and three cycles were administered after radiotherapy. Fluorouracil (FU) 800 mg/m2 daily was administered as a continuous intravenous (IV) infusion (days 1–5). Cisplatin 15 mg/m2 (days 1–5) was delivered during a 1-h IV infusion, preceded and followed by a 2-h IV infusion of normal saline 1 L. |
|  | ENI | 49 | The CTV elective nodal areas were the supraclavicular lymph node basins (if the disease was located at or above carina) or celiac lymph node basins (if the disease was located in the distal esophagus). The planning target volumes (PTV) were created by adding margins of 0.5 cm to the respective CTVs. | 45-54 Gy, 1.8-2.0/ fraction | 3D-CRT | CCRT | The same as IFI. |
| D.J Li,  2016 | IFI | 43 | Esophageal wall thickness of more than 0.5cm and positive LNs were included in the gross tumor volume (GTV). CTV only encompassed 3 cm proximal and distal margins and 0.5-0.8 cm radial margin on the basis of GTV. Uninvolved regional LNs were not encompassed in the CTV. At the same time, the volume of CTV was appropriately adjusted on the basis of the human anatomic structure so that the maximum dosage in the spinal cord did not exceed 45 Gy. PTV encompassed 1 cm proximal and distal margins, 0.5 cm radial margin on the basis of CTV. | 50-56 Gy/ 29-33 fractions | 3D-CRT  IMRT | RT±CT | Chemotherapy began on day 1, concurrent with the beginning of radiation. The chemotherapeutic regimen consisted of two cycles of cisplatin (20 mg/m2/day on day 1 to day 4) and 5-fluorouracil (5-FU) (500 mg/m2/day as a continuous infusion from day 1 to day 4) every 28 days. |
|  | ENI | 36 | the first clinical target volumes (CTV1) encompassed the primary tumor, the malignant LNs, and 3 cm proximal and distal margins; a 0.5-0.8 cm radial margin was added to the GTV. The first planning  target volumes (PTV1) encompassed 1 cm proximal and distal margins, 0.5 cm radial margin on the basis of CTV1, with a total dose of 54-60 Gy/29-33F. The second CTV (CTV2) encompassed only 3-cm proximal and distal margins; a 0.5 cm radial margin was added to the GTV, and uninvolved regional LNs were encompassed in the CTV2. The second PTV (PTV2) encompassed 1-cm proximal and distal margins, 0.5 cm radial margin on the basis of CTV2, with a total dose of 50-54 Gy/29-33F. | PTV1: 54-60 Gy/ 29-33 fractions  PTV2: 50-54 Gy/ 29-33 fractions | IMRT | RT±CT | The same as IFI. |
| Bai,  2016 | IFI | 15 | GTV refers to the gross tumor of the esophagus and enlarged lymph nodes; CTV refers to the longitudinal expansion of the primary tumor of the esophagus by 3 cm, axial expansion of 0.8-1.0 cm, and the involvement of enlarged lymph nodes. PTV refers to the longitudinal expansion of CTV by 0.5 cm, axial expansion expand 1.0cm outward. | PTV: 50.4-60.0 Gy/ 28-30 fractions (1.8-2.0 Gy/ fraction)  GTV: 60-66 Gy/ 28-30 fractions (2.00-2.27 Gy/ fraction) | IMRT | RT±CT | Cisplatin (20-30 mg/d, d1-d5), based on the combined chemotherapy regimen, the combined chemotherapy drugs are fluorouracil or paclitaxel, fluorouracil 1g/d, d1-d5; paclitaxel is 135-175 mg/m2, d1. 28d is 1 cycle, 1-6 cycles of chemotherapy. |
|  | ENI | 48 | CTV is the primary tumor of esophagus with longitudinal expansion of 3 cm and axial expansion of 0.8-1.0 cm, and includes double-lock, paraesophageal and lymphatic drainage areas of 2, 4, 5, and 7 (all cervical segments). bag and double lock, paraesophageal, some patients bag and zone 2 or 4). | PTV: 50.4-60.0 Gy/ 28-30 fractions (1.8-2.0 Gy/ fraction)  GTV: 60-66 Gy/ 28-30 fractions (2.00-2.27 Gy/ fraction) | IMRT | RT±CT | The same as IFI. |
| Dong,  2015 | IFI | 119 | GTV is the thickening of the esophagus wall ≥ 0.5 cm on CT or the diameter of the esophagus lumen without air is ≥ 1.0 cm, and is determined with reference to the results of esophageal barium meal angiography and endoscopy; CTV is the outward expansion of the GTV axis by 0.5 to 1.0 cm. 0.8 cm, 1.5-2.0 cm up and down, and appropriate modification and adjustment according to the anatomical barrier; PTV is the outward expansion of 0.5-1.0 cm for the CTV. GTVnd delineates the lymph nodes that are clinically confirmed to be metastatic. CTVnd refers to GTVnd expanding 0.5cnq in all directions, and PTVnd refers to CTVnd expanding 0.5 to 0.8 cm evenly. | PTV/PTV-nd: 56-66 Gy, 1.8-2.0 Gy/ fraction | 3D-CRT | RT±CT | Received platinum-based chemotherapy. |
|  | ENI | 126 | CTV1: Primary lesions located in the neck or upper thoracic segment include bilateral supraclavicular, paraesophageal, zone 2, 4, 5, and 7, to a range of 3.0 to 4.0 cm below the carina; mid-thoracic carcinoma For lower thoracic cancer, it includes paraesophageal, 4, 5, 7, 8, 9, and cardiac The lymphatic drainage area of the para, left stomach and paraceliac trunk, evenly expanded by 0.5 to 1.0 cIn, is PTV1. | PTV/PTV-nd: 56-66 Gy, 1.8-2.0 Gy/ fraction  PTV1: 46-54 Gy/ 1.8-2.0 Gy/ fraction | 3D-CRT | RT±CT | The same as IFI. |
| Yamashita,  2015 | IFI | 119 | Tumor volume was visualized on computed tomography (CT) and/or PET and endoscopic extension and used to define gross tumor volume (GTV) for each patient. All LNs with a diameter at least one cm in short axis in CT or positive by 18FDG-PET (excluding physiological accumulation) were included in the GTV. The clinical target volume (CTV) was generated by using no radial margin and 2 cm longitudinal margins to the GTV-primary, and by using no margin for the GTV-LNs. The planning target volume (PTV) was then generated by applying a 5 mm radial margin and a 10 mm longitudinal margin to the CTV. | 50.4 Gy, 1.8 Gy/ fraction | 3D-CRT | CCRT±CT | Chemotherapy consisted of two cycles of 5-fluorouracil (5-FU) (800 mg/m2/day, days 1–4 & days 29–32, continuous) combined with cisplatin or nedaplatin (NDP) (80 mg/m2, day 1 & day 29, bolus); standard techniques were used for hydration and alkalization. For a case 75 years or older, reductions were made to an 80 % dose. Chemotherapy was started on the first day of  irradiation. After concurrent CRT, in the adjuvant setting, an additional one or two cycles of the same dose of chemotherapy were given for patients who still had sufficient bone-marrow function and performance status and who did not refuse additional chemotherapy. |
|  | ENI | 120 | Tumor Volume (GTV) was defined for each subject as tumor volume was visualized on CT and endoscopic extension. All LNs with a diameter at least one cm in short axis in CT or positive by FDG-PET (excluding physiological accumulation) were included in the GTV. GTV included primary cancer and metastatic lymph nodes. Clinical target volume (CTV) was defined as the whole thoracic esophagus (= from the supraclavicular fossae to the esophagogastric junction) including GTV plus 5 mm margin. CTV comprised up to M1a LNs as well as regional LNs including positive LNs. PTV was created by adding margins of 5–10 mm to the respective CTVs. | 50-50.4 Gy, 1.8-2.0 Gy/ fraction | 3D-CRT | CCRT±CT | The same as IFI. |
| W Jing,  2015 | IFI | 83 | The gross tumor volume (GTV) was defined as any visible esophageal lesions (GTVt) shown on CT, barium esophagography, localizable CT or diagnostic CT images and PET/CT scans, plus any involved LNs (GTVnd). The clinical target volume (CTV) consisted of CTVt and CTVnd. The CTVt was defined as the GTVt plus a 2.0–4.0 cm margin superior and inferior to the primary tumor and a lateral margin of 0.8–1.0 cm. CTVnd was defined as the GTVnd plus a 0.5–1.0 cm radial margin. The planning target volume (PTV) was defined as the CTV plus a 0.5–1.0 cm radial margin. | 50-68.4 Gy, 1.8-2.0 Gy/ fraction | 3D-CRT  IMRT | RT±CT | 2 cycles of platinum-based chemotherapy with combined 5-fluorouracil and a taxane (docetaxel or paclitaxel). |
|  | ENI | 54 | The GTV was defined in the same way as that for IFI. CTV was defined as GTV plus a 2.0–5.0 cm craniocaudal margin with a 0.8–1.0 cm lateral margin and the areas at risk for elective nodal regions (such as lower cervical, periesophageal, mediastinal and perigastric LNs). Elective treatment of nodal regions depended upon the location of the primary tumor. For example, the supraclavicular nodes and the celiac nodes were included for the upper thoracic esophagus and the lower esophagus, respectively. The definition of PTV was the same as that for IFI. | 50-68.4 Gy, 1.8-2.0 Gy/ fraction  40 Gy, 1.8-2.0 Gy/ fraction | 3D-CRT  IMRT | RT±CT | The same as IFI. |
| Cao,  2015 | IFI | 110 | The esophageal tumor was delineated as the tumor target volume (GTV) according to chest localization CT, esophageal barium meal angiography and electronic gastroscopy. o～3.0cm is the clinical target volume (CTV); CTV is evenly spread out 0.5～1.0cm is the planning target volume (PTV). | PTV: 54-68 Gy/ 27-34 fractions | 3D-CRT  IMRT | RT±CT | Cisplatin-based, calcium folinate, 200 mg/time, intravenous infusion, d1-d5; Tegafur, 1 g/time, intravenous infusion, d1-d5; cisplatin, 20 mg/time, intravenous bolus, d1 ~ d5 and paclitaxel, 240 mg/time, intravenous infusion, d1; cisplatin, 20 mg/time, intravenous bolus injection, d1-d5; 21d is one cycle, with a total of 2-6 cycles. |
|  | ENI | 48 | The delineation of the patient's GTV, CTV and PTV is the same as that of the patients with IFI. The lymph node drainage areas in the upper middle and upper thoracic segments include bilateral locking, area 2, area 4, area 5, area 6, area 7 and area 8, The lower boundary is 4 to 5 cm below the tracheal carina; the midthoracic lymph node drainage area includes areas 2, 4, 5, 6, 7 and 8, and the lower boundary is the cardia lymph node area; the lower thoracic drainage area includes areas 4 and 5 , zone 6, zone 7, zone 8, paracardium, lesser curvature of the stomach and left gastric lymph node region. The delineated lymph node drainage area is defined as CTV-N, and on the basis of CTV-N, 0.3～0.5cm, spread out from top to bottom 1. 0～1.5cm is PTV-N. | PTV: 56-70 Gy  PTV-N: 45-50.4 Gy/ 25-28 fractions | 3D-CRT  IMRT | RT±CT | The same as IFI. |
| Liu,  2014 | IFI | 99 | The gross tumor volume (GTV) was defined as any primary tumors shown on the CT, or barium esophagogram, and mediastinal lymph nodes with  the short axis of >10 mm and the cervical lymph nodes  with the short axis of > =10 mm. For the planning target volume (PTV), a 1 cm margin was added around GTV, but 3 cm margins in the esophageal long axis superiorly and inferiorly to encompass potential submucosal invasions. | 60-68.4 Gy | - | CRT | PF regimen(cisplatin and 5-fluorouracil), TP (taxane and cisplatin), and TPF (taxane, cisplatin and 5-fluorouracil). |
|  | ENI | 70 | The definition for GTV was the same as that of IFI.  The planning target volume (PTV) enclosed GTV and clinical target volume (CTV) which covered supraclavicular area with the upper margin at the caudal edge of cricoid cartilage, inferior margin at the sternal notch. | 60-68.4 Gy,  50.4-54 Gy | - | CRT | The same as IFI. |
| Zang,  2013 | IFI | 35 | Gross tumor volume (GTV) included tissues with esophageal wall thickness > 0.5 cm on CT images and positive lymph nodes, as well as esophageal barium films and esophagoscopy results. CTV only includes GTV and the surrounding 0.5cm range, including 3cm normal esophagus in the direction of head and feet. PTV is CTV and its surrounding 0.5cm range. | 54 Gy/ 30 fractions (1.8 Gy/ fraction) | 3D-CRT | CCRT+CT | Paclitaxel 100mg/m2, intravenous bolus injection, d1, d8; Cisplatin 75 mg/m2, intravenous bolus injection, d1; 21d is a cycle. 2 cycles of concurrent chemotherapy. After the end of radiotherapy, 2 cycles of chemotherapy were given. |
|  | ENI | 38 | The clinical target volume of CTV1 included subclinical lesions and mediastinal lymphatic drainage areas around the GTV, and included a 3 cm normal esophagus in the direction of the head and feet. The planned target volume PTV1 includes CTV1 and the surrounding 0.5cm range, and the head and foot direction is placed outside the 1cm range. CTV2 only includes the GTV and its surrounding 0.5cm range. PTV2 is CTV2 and the surrounding 0.5cm range. | 54 Gy/ 30 fractions (1.8 Gy/ fraction) | 3D-CRT | CCRT+CT | The same as IFI. |
| Shen,  2013 | IFI | 102 | GTV includes gross esophageal tumor and metastatic lymph nodes, and the corresponding external expansion is CTV and PTV. | 50-70 Gy/ 25-35 fractions | 3D-CRT | RT±CT | 5-Fluorouracil (800 mg/m2, d1-5) and cisplatin (80 mg/m2, d1 or 20 mg/m2, d1-5) |
|  | ENI | 21 | The lymph node drainage area in the upper midthoracic segment includes bilateral supraclavicular and areas 2, 4, 5, 6, 7 and 8, and the lower boundary is 4 to 5 cm below the tracheal carina; the lymph node drainage area in the middle thoracic segment includes areas 2, 4, 5, 6, Areas 7 and 8, the lower bound is the lymph node area of the cardia; the drainage area of the lower thoracic segment includes the paracardium, the lesser curvature of the stomach, the left gastric lymph node area, and the areas 4, 5, 6, 7 and 8. The delineated target area of the lymph node drainage area was defined as CTV-N. On the basis of CTV-N, 0.3-0.5 cm in the front, back, left and right directions, and 1.0-1.5 cm in the upper and lower directions were externalized as PTV-N. | PTV-N: 45.0-50.4 Gy/ 25-25 fractions  PTV: 56-70 Gy, 1.8-2.0 Gy/ fraction | 3D-CRT | RT±CT | The same as IFI. |
| M Li,  2012 | IFI | 49 | GTV is the primary tumor of the esophagus. The length of the tumor is mainly based on esophageal barium meal angiography, and with reference to the results of endoscopy, the front, back, left and right sides are determined according to the largest layer of the lesion and considered as the enlarged lymph node GTV-nd, CT display. The CTV included the primary tumor expanding 3 cm above and below, the surrounding area expanding 0.8-1.0 cm, and the area of enlarged lymph nodes; the CTV in the expanded field group included the primary tumor expanding 3 cm above and below, the surrounding area expanding by 0.8-1.0 cm, and the esophagus of this segment. the regional lymph node drainage area. PTV is the CTV that expands 1 cm from top to bottom and 0.5 cm from the periphery. In order to reduce the irradiation of the surrounding lung tissue, the target area named PTV1 included the esophageal GTV up and down 1.5 cm, the peripheral extension 0.8-1.0 cm, and the lymphatic involvement area. | PTV: 50 Gy/ 25 fractions  booster dose to PTV1: 10-12 Gy/ 5-6 fractions | 3D-CRT | RT±CT | FP program (cisplatin + tegafur) |
|  | ENI | 45 | CTV: Upper thoracic regional lymph nodes include: supraclavicular lymphatic drainage area, paraesophageal, level 2, level 4, level 5, level 7. Regional lymph nodes in midthoracic esophageal cancer include paraesophageal, level 2, level 4, level 5, and level 7. Regional lymph nodes of lower thoracic esophageal cancer include paraesophageal, level 4, level 5, level 7, and left gastric and paracardial lymphatic drainage areas. | PTV: 50 Gy/ 25 fractions  booster dose to PTV1: 10-12 Gy/ 5-6 fractions | 3D-CRT | RT±CT | The same as IFI. |
| Ma,  2011 | IFI | 51 | Cytologically positive lymph nodes by ultrasound fine needle aspiration biopsy or malignant nodes were regarded as gross tumor volume (GTV). CTV only encompassed 3-cm proximal and distal margins and 0.5–0.8-cm radial margin on the basis of GTV for cervical and upper-thoracic esophageal cancer. Uninvolved regional lymph nodes were not encompassed in the CTV. At the same time, the volume of CTV was appropriately adjusted on the basis of the human anatomic structure so that the maximum dosage in the spinal cord did not exceed 45Gy. PTV encompassed 1-cm proximal and distal margins, 0.5-cm radial margin on the basis of CTV. | 59.4 Gy (41.4 Gy/ 23 fractions/ 1.8 Gy/ fraction) | 3D-CRT | CCRT+CT | The concurrent chemotherapy consisted of 100 mg/m2 of paclitaxel each cycle on days 1 and 8 plus 75 mg/m2 of cisplatin on day 1 with adequate prehydration, mannitol, and antiemetic coverage by intravenous infusion every 3 weeks. Each cycle of chemotherapy was repeated every 3 weeks. Patients received an additional 2 cycles of chemotherapy after the completion of radiation with a 1-month rest. |
|  | ENI | 51 | The first clinical target volumes (CTV1) encompassed the primary tumor, the middle-lower cervical, supraclavicular, and superior mediastinal nodes, and 3-cm proximal and distal margins; a 0.5–0.8-cm radial margin was added to the GTV. Uninvolved regional  lymph nodes were encompassed in the CTV. The first planning target volumes (PTV1) encompassed 1-cm proximal and distal margins, 0.5-cm radial margin on the basis of CTV1. The second CTVs (CTV2) only encompassed 3-cm proximal and distal margins; a 0.5-cm radial margin was added to the GTV. The second PTVs (PTV2) encompassed 1-cm proximal and distal margins, 0.5-cm radial margin on the basis of CTV2. | 59.4 Gy (41.4 Gy/ 23 fractions/ 1.8 Gy/ fraction + 18 Gy, 1.5Gy/ fraction, twice a day) | 3D-CRT | CCRT+CT | The same as IFI. |

**Abbreviations**: IFI, involved field irradiation; ENI, elective nodal irradiation; 2D-RT, two-dimensional radiation therapy; 3D-CRT, three-dimensional conformal radiotherapy; IMRT, intensity modulated radiotherapy; IGRT, image-guided radiation therapy; VMAT, volumetric-modulated arc therapy; RT, radiation therapy; CT, chemotherapy; CRT, chemoradiotherapy; CCRT, concurrent chemoradiotherapy.
