# Supplementary material for: The role of involved field irradiation versus elective nodal irradiation in definitive radiotherapy or chemoradiotherapy for esophageal cancer- a systematic review and meta-analysis
Source: Front Oncol. 2022 Nov 2;12:1034656. doi: 10.3389/fonc.2022.1034656 (PMC9666894; doi:10.3389/fonc.2022.1034656)
Supplement: Supplementary file 1 [file DataSheet_1.zip › supplementary materials/Supplementary Table/Supplementary Table. 3 Summary of Overall Survival and Profession Free Survival.docx]

**Supplementary Table. 3** Summary of Overall Survival and Profession Free Survival

| Study | Radiotherapy target size | N analyzed | Median Overall Survival  (mo, range) | 1year OS  rates | 2year OS  rates | 3year OS  rates | 5year OS  rates | Median Progression Free Survival  (mo, range) | 1year PFS rates | 2year PFS rates | 3year PFS rates | 5year PFS rates |
| --- | --- | --- | --- | --- | --- | --- | --- | --- | --- | --- | --- | --- |
| Zhu,  2020 | IFI | 272 | 21.8  (16.8-26.8） | 75.0 | - | 31.8 | 17.2 | - | 54.0 | - | 20.9 | 12.7 |
|  | ENI | 272 | 29.0  (22.3-35.7） | 79.0 | - | 43.7 | 24.9 | - | 65.3 | - | 31.7 | 18.4 |
| Xie,  2020 | IFI | 88 | 20.87 | 72.7 | 44.5 | 31.8 | 19.6 | - | - | - | - | - |
|  | ENI | 88 | 26.50 | 79.7 | 52.6 | 45.4 | 34.8 | - | - | - | - | - |
| Nakatani,  2020 | IFI | 78 | - | 100.0 | 97.6 | 97.4 | 90.5 | - | 93.9 | 83.1 | 80.8 | 77.6 |
|  | ENI | 117 | - | 97.7 | 87.0 | 78.0 | 72.5 | - | 92.9 | 79.6 | 70.6 | 57.9 |
| Lyu,  2020 | IFI | 98 | 34.9 | 82.5 | 62.9 | 48.7 | 30.7 | 21.4 | 64.4 | 41.3 | 30.8 | 27.4 |
|  | ENI | 94 | 32.5 | 84.6 | 63.7 | 45.1 | 29.8 | 20.3 | 58.9 | 41.5 | 34.2 | 26.9 |
| Q.F Li,  2019 | IFI | 314 | 21.5  (17.9-25.1) | 73.2 | 47.4 | 32.2 | 19.0 | - | 56.7 | 36.9 | 25.5 | 15.9 |
|  | ENI | 157 | 26.8  (17.9-35.7) | 77.1 | 52.8 | 42.0 | 26.1 | - | 61.8 | 38.6 | 30.6 | 19.1 |
| Wang,  2018 | IFI | 276 | 25.9 | 73.0 | - | 40.4 | 25.4 | - | - | - | - | - |
|  | ENI | 83 | 29.0 | 81.7 | - | 43.5 | 24.8 | - | - | - | - | - |
| Sun,  2018 | IFI | 49 | 36.0  (21.9-50.1) | 87.8 | 71.3 | 49.4 | 32.3 | 20.0  (7.87-39.2) | 70.5 | 50.0 | 43.1 | 32.6 |
|  | ENI | 77 | 38.0  (26.1-49.9) | 92.2 | 65.7 | 52.0 | 28.9 | 30.0  (17.4-44.6) | 70.5 | 58.5 | 48.2 | 29.7 |
| Yisikandaer,  2018 | IFI | 104 | 32.0 | 83.6 | 62.1 | 44.5 | 31.5 | 22.0 | 70.1 | 45.0 | 35.9 | - |
|  | ENI | 101 | 28.0. | 84.1 | 57.3 | 39.4 | 31.6 | 20.0 | 71.9 | 42.3 | 32.7 | - |
| Zhao,  2017 | IFI | 40 | - | - | - | - | - | - | - | - | - | - |
|  | ENI | 46 | - | - | - | - | - | - | - | - | - | - |
| Su,  2017 | IFI | 47 | - | 89.0 | 57.2 | 35.0 | 20.0 | - | 59.9 | 44.8 | 36.4 | 17.6 |
|  | ENI | 49 | - | 85.2 | 65.8 | 53.3 | 31.0 | - | 77.9 | 47.2 | 36.6 | 23.4 |
| Zh Jing,  2017 | IFI | 38 | 17.3  (3.17-142.2） | 68.5 | 36.6 | 26.4 | 23.7 | - | 40.5 | 28.0 | 24.6 | 21.1 |
|  | ENI | 51 | 20.13  (2.4-131.7） | 69.2 | 42.1 | 38.1 | 33.8 | - | 54.1 | 40.6 | 38.6 | 33.1 |
| Park,  2016 | IFI | 50 | - | - | - | 48.0 | - | - | 43.2 | 32.1 | 25.0 | 24.5 |
|  | ENI | 49 | - | - | - | 52.0 | - | - | 65.6 | 49.2 | 46.0 | 42.7 |
| D.J Li,  2016 | IFI | 43 | 21.2  (13.6-32.8) | 88.4 | 23.3 | 18.6 | - | - | - | - | - | - |
|  | ENI | 36 | 22.9  (15.7-30.3) | 74.4 | 36.1 | 22.2 | - | - | - | - | - | - |
| Bai,  2016 | IFI | 15 | - | - | - | - | - | - | - | - | - | - |
|  | ENI | 48 | - | - | - | - | - | - | - | - | - | - |
| Dong,  2015 | IFI | 119 | - | 76.5 | 46.7 | 29.5 | 16.5 | - | 53.5 | 38.6 | 28.5 | 18.5 |
|  | ENI | 126 | - | 76.1 | 51.8 | 43.4 | 25.5 | - | 64.6 | 44.8 | 37.5 | 31.0 |
| Yamashita,  2015 | IFI | 119 | 38.9  (14.9-62.8) | 70.8 | 58.7 | 51.6 | - | - | - | - | - | - |
|  | ENI | 120 | 21.3  (16.1-26.5) | 65.8 | 45.8 | 34.8 | - | - | - | - | - | - |
| W Jing,  2015 | IFI | 83 | 15.5 | 59.0 | 30.7 | 21.7 | 0 | 11.0 | 43.8 | 23.6 | 21.0 | 0 |
|  | ENI | 54 | 17.0 | 68.5 | 41.0 | 26.4 | 10.1 | 13.0 | 52.1 | 36.6 | 20.6 | 4.1 |
| Cao,  2015 | IFI | 110 | - | - | - | - | - | - | - | - | - | - |
|  | ENI | 48 | - | - | - | - | - | - | - | - | - | - |
| Liu,  2014 | IFI | 99 | - | - | - | 49.0 | - | - | - | - | - | - |
|  | ENI | 70 | - | - | - | 47.0 | - | - | - | - | - | - |
| Zang,  2013 | IFI | 35 | 21.74  (17.69-25.79) | 71.8 | 44.7 | 25.7 | - | - | - | - | - | - |
|  | ENI | 38 | 21.96  (17.91-26.01) | 66.1 | 60.0 | 45.4 | - | - | - | - | - | - |
| Shen,  2013 | IFI | 102 | - | 86.3 | - | 49.0 | 35.2 | - | - | - | - | - |
|  | ENI | 21 | - | 90.5 | - | 47.6 | 42.9 | - | - | - | - | - |
| M Li,  2012 | IFI | 49 | 28.0 | 66.9 | 55.4 | 36.2 | - | - | 63.8 | 43.7 | 43.7 | - |
|  | ENI | 45 | 24.0 | 68.6 | 48.4 | 35.5 | - | - | 64.8 | 45.7 | 40.6 | - |
| Ma,  2011 | IFI | 51 | 33.7  (27.8-39.6） | 100.0 | 87.5 | 32.0 | - | - | - | - | - | - |
|  | ENI | 51 | 32.7  (29.1-36.3） | 100.0 | 84.0 | 41.3 | - | - | - | - | - | - |

**Abbreviations**: IFI, involved field irradiation; ENI, elective nodal irradiation; OS, overall survival; PFS, profession free survival.
