# Supplementary material for: The role of involved field irradiation versus elective nodal irradiation in definitive radiotherapy or chemoradiotherapy for esophageal cancer- a systematic review and meta-analysis
Source: Front Oncol. 2022 Nov 2;12:1034656. doi: 10.3389/fonc.2022.1034656 (PMC9666894; doi:10.3389/fonc.2022.1034656)
Supplement: Supplementary file 1 [file DataSheet_1.zip › supplementary materials/Supplementary Table/Supplementary Table. 4 Summary of Local Contral Rates and Incidence of toxicity related to IFI or ENI.docx]

**Supplementary Table. 4** Summary of Local Contral Rates and Incidence of toxicity related to IFI or ENI

| Study | Radiotherapy target size | N analyzed | 1year  LCR | 2year  LCR | 3year  LCR | AE (%) | AP (%) | ≥2 AE(%) | ≥ 2 AP(%) | ≥ 3 AE(%) | ≥ 3 AP(%) | LE (%) | LP (%) | ≥ 2 LE(%) | ≥ 2 LP(%) | ≥ 3 LE(%) | ≥ 3 LP(%) |
| --- | --- | --- | --- | --- | --- | --- | --- | --- | --- | --- | --- | --- | --- | --- | --- | --- | --- |
| Zhu,  2020 | IFI | 272 | - | - | - | - | - | 31.3 | 11.8 | 4.8 | 4.8 | - | - | - | - | - | - |
|  | ENI | 272 | - | - | - | - | - | 33.8 | 13.6 | 5.9 | 2.2 | - | - | - | - | - | - |
| Xie,  2020 | IFI | 88 | - | - | - | - | - | - | - | - | - | - | - | - | - | - | - |
|  | ENI | 88 | - | - | - | - | - | - | - | - | - | - | - | - | - | - | - |
| Nakatani,  2020 | IFI | 78 | - | - | - | - | - | - | - | - | - | - | - | - | - | - | - |
|  | ENI | 117 | - | - | - | - | - | - | - | - | - | - | - | - | - | - | - |
| Lyu,  2020 | IFI | 98 | - | - | - | - | - | 37.4 | 8.7 | - | - | - | - | - | - | - | - |
|  | ENI | 94 | - | - | - | - | - | 19.2 | 18.8 | - | - | - | - | - | - | - | - |
| Q.F Li,  2019 | IFI | 314 | - | - | - | 59.6 | 35.0 | 29.3 | 14.0 | 4.45 | 2.87 | - | - | - | - | - | - |
|  | ENI | 157 | - | - | - | 81.5 | 26.1 | 33.8 | 11.5 | 4.46 | 2.55 | - | - | - | - | - | - |
| Wang,  2018 | IFI | 276 | - | - | - | - | - | - | - | - | - | - | - | - | - | - | - |
|  | ENI | 83 | - | - | - | - | - | - | - | - | - | - | - | - | - | - | - |
| Sun,  2018 | IFI | 49 | - | - | - | - | - | - | - | - | - | - | - | - | - | - | - |
|  | ENI | 77 | - | - | - | - | - | - | - | - | - | - | - | - | - | - | - |
| Yisikandaer,  2018 | IFI | 104 | - | - | - | - | - | 19.2 | 8.7 | 4.8 | 3.8 | - | - | - | - | - | - |
|  | ENI | 101 | - | - | - | - | - | 34.7 | 18.8 | 15.8 | 8.9 | - | - | - | - | - | - |
| Zhao,  2017 | IFI | 40 | - | - | - | - | - | - | - | - | - | - | - | - | - | - | - |
|  | ENI | 46 | - | - | - | - | - | - | - | - | - | - | - | - | - | - | - |
| Su,  2017 | IFI | 47 | 65.4 | 52.4 | 46.5 | 59.6 | 14.9 | 2.1 | 0 | 2.1 | 0 | - | - | - | - | - | - |
|  | ENI | 49 | 80.6 | 73.4 | 57.4 | 79.6 | 12.2 | 2.0 | 0 | 2.0 | 0 | - | - | - | - | - | - |
| Zh Jing,  2017 | IFI | 38 | - | - | - | 79.0 | - | - | - | 13.2 | - | - | - | - | - | - | - |
|  | ENI | 51 | - | - | - | 96.1 | - | - | - | 65.8 | - | - | - | - | - | - | - |
| Park,  2016 | IFI | 50 | - | - | - | - | - | - | - | - | - | - | - | - | - | 2.0 | 6.0 |
|  | ENI | 49 | - | - | - | - | - | - | - | - | - | - | - | - | - | 4.1 | 12.2 |
| D.J Li,  2016 | IFI | 43 | - | - | - | - | - | - | - | - | - | - | - | - | - | - | - |
|  | ENI | 36 | - | - | - | - | - | - | - | - | - | - | - | - | - | - | - |
| Bai,  2016 | IFI | 15 | - | - | - | - | - | - | - | - | - | - | - | - | - | - | - |
|  | ENI | 48 | - | - | - | - | - | - | - | - | - | - | - | - | - | - | - |
| Dong,  2015 | IFI | 119 | 60.4 | - | 37.9 | - | - | - | - | - | - | - | - | - | - | - | - |
|  | ENI | 126 | 70.1 | - | 51.6 | - | - | - | - | - | - | - | - | - | - | - | - |
| Yamashita,  2015 | IFI | 119 | - | - | - | - | - | - | - | 1.7 | 2.5 | - | - | - | - | - | - |
|  | ENI | 120 | - | - | - | - | - | - | - | 1.7 | 5.8 | - | - | - | - | - | - |
| W Jing,  2015 | IFI | 83 | - | - | - | - | - | 16.9 | - | 6.0 | 12.0 | - | - | - | - | - | - |
|  | ENI | 54 | - | - | - | - | - | 38.9 | - | 18.5 | 7.4 | - | - | - | - | - | - |
| Cao,  2015 | IFI | 110 | - | - | - | - | - | - | - | - | - | - | - | - | - | - | - |
|  | ENI | 48 | - | - | - | - | - | - | - | - | - | - | - | - | - | - | - |
| Liu,  2014 | IFI | 99 | - | - | - | - | - | - | - | 6.1 | 2.0 | - | - | - | - | 2.0 | - |
|  | ENI | 70 | - | - | - | - | - | - | - | 5.7 | 4.3 | - | - | - | - | 2.9 | - |
| Zang,  2013 | IFI | 35 | 93.0 | 71.6 | 71.5 | 57.1 | 45.7 | 48.6 | 37.1 | 11.4 | 2.9 | 37.1 | 48.6 | 22.9 | 42.9 | 14.3 | 25.7 |
|  | ENI | 38 | 87.0 | 80.0 | 80.0 | 76.3 | 65.8 | 68.4 | 50.0 | 38.5 | 13.2 | 38.5 | 31.6 | 23.7 | 18.4 | 14.3 | 10.5 |
| Shen,  2013 | IFI | 102 | - | - | - | - | - | 34.3 | 10.8 | - | - | - | - | - | - | - | - |
|  | ENI | 21 | - | - | - | - | - | 38.1 | 38.1 | - | - | - | - | - | - | - | - |
| M Li,  2012 | IFI | 49 | 72.4 | 59.4 | 54.5 | 77.6 | 30.6 | 32.7 | 6.1 | 6.1 | 2.0 | - | - | - | - | - | - |
|  | ENI | 45 | 69.5 | 58.4 | 46.0 | 86.7 | 35.6 | 42.2 | 11.1 | 15.6 | 0 | - | - | - | - | - | - |
| Ma,  2011 | IFI | 51 | 90.0 | 80.1 | 80.1 | 62.7 | 54.9 | 51.0 | 39.2 | 13.7 | 9.8 | 21.6 | 9.8 | 19.6 | 5.9 | 9.8 | 2.0 |
|  | ENI | 51 | 92.8 | 92.8 | 85.7 | 72.5 | 47.1 | 60.8 | 33.3 | 21.6 | 11.8 | 21.6 | 23.5 | 17.6 | 15.7 | 9.8 | 5.9 |

**Abbreviations**: IFI, involved field irradiation; ENI, elective nodal irradiation; LCR, local contral rates; AE, acute esophagitis; AP, acute pneumonia; LE, late esophagitis; LP, late pneumonia.
