# Supplementary material for: The role of involved field irradiation versus elective nodal irradiation in definitive radiotherapy or chemoradiotherapy for esophageal cancer- a systematic review and meta-analysis
Source: Front Oncol. 2022 Nov 2;12:1034656. doi: 10.3389/fonc.2022.1034656 (PMC9666894; doi:10.3389/fonc.2022.1034656)
Supplement: Supplementary file 1 [file DataSheet_1.zip › supplementary materials/Supplementary Table/Supplementary Table. 5 Cochrane Risk of Bias.docx]

**Supplementary Table. 5** Cochrane Risk of Bias

| **Study** | **Selection Bias** | | **Performance Bias** | | **Detection Bias** | **Attrition Bias** | **Reporting Bias** | **Other Bias** |
| --- | --- | --- | --- | --- | --- | --- | --- | --- |
|  | **Random** | **Allocation** | **Blinding of** | **Blinding of** | **Blinding of** | **Incomplete** | **Selective** |  |
|  | **sequence** | **concealment** | **participants** | **healthcare** | **outcome** | **outcome data or** | **reporting** |  |
|  | **generations** |  |  | **providers** | **assessment** | **loss to follow-up** |  |  |
| Xie,2020 | + | ? | - | - | + | + | + | + |
| Lyu,2020 | ? | ? | ? | ? | + | + | + | + |
| Yisikandaer,2018 | + | + | ? | ? | + | + | + | + |
| Zang,2013 | + | ? | ? | ? | + | + | + | + |
| M Li,2012 | ? | ? | ? | ? | + | + | + | + |
| Ma,2011 | ? | ? | ? | ? | + | + | + | + |

*+ = low risk, - = high risk, ? =unclear*
