# Supplementary material for: The role of involved field irradiation versus elective nodal irradiation in definitive radiotherapy or chemoradiotherapy for esophageal cancer- a systematic review and meta-analysis
Source: Front Oncol. 2022 Nov 2;12:1034656. doi: 10.3389/fonc.2022.1034656 (PMC9666894; doi:10.3389/fonc.2022.1034656)
Supplement: Supplementary file 1 [file DataSheet_1.zip › supplementary materials/Supplementary Table/Supplementary Table. 6 Newcastle-Ottawa Risk of Bias and AHRQ Standard.docx]

**Supplementary Table. 6** Newcastle-Ottawa Risk of Bias and AHRQ Standard

| Study | Selection Score | Comparability Score | Outcome Score | AHRQ Standard |
| --- | --- | --- | --- | --- |
| Zhu,2020 | **** | ** | *** | Good |
| Nakatani,2020 | **** | ** | *** | Good |
| Q.F Li,2019 | **** | ** | *** | Good |
| Wang,2018 | **** | * | *** | Good |
| Sun,2018 | **** | ** | *** | Good |
| Zhao,2017 | **** | * | *** | Good |
| Su,2017 | **** | ** | *** | Good |
| Zh Jing,2017 | **** | ** | *** | Good |
| Park,2016 | **** | ** | ** | Good |
| D.J Li,2016 | **** | * | ** | Good |
| Bai,2016 | **** | * | ** | Good |
| Dong,2015 | **** | ** | ** | Good |
| Yamashita,2015 | **** | ** | *** | Good |
| W Jing,2015 | **** | ** | *** | Good |
| Cao,2015 | **** | * | *** | Good |
| Liu,2014 | **** | * | ** | Good |
| Shen,2013 | **** | * | *** | Good |
