# Supplementary material for: The role of involved field irradiation versus elective nodal irradiation in definitive radiotherapy or chemoradiotherapy for esophageal cancer- a systematic review and meta-analysis
Source: Front Oncol. 2022 Nov 2;12:1034656. doi: 10.3389/fonc.2022.1034656 (PMC9666894; doi:10.3389/fonc.2022.1034656)
Supplement: Supplementary file 1 [file DataSheet_1.zip › supplementary materials/Supplementary Table/Supplementary Table. 7 Grading of Recommendations, Assessment, Development, and Evaluation (GRADE) of meta-analyzed outcomes.docx]

**Supplementary Table. 7** Grading of Recommendations, Assessment, Development, and Evaluation (GRADE) of meta-analyzed outcomes

| Certainty assessment | | | | | | | Summary of findings | |
| --- | --- | --- | --- | --- | --- | --- | --- | --- |
| N of  participants | Risk of  bias | Inconsistency | Indirectness | Imprecision | Publication  bias | Overall  certainty of  evidence | Pooled effect size  (95% CI) | Anticipated effects |
| Overall Survival at 1-Year | | | | | | | | |
| 3545  (18 studies) | not  serious | not  serious | not  serious | not  serious | none | ⊕⊕⊕⊕  HIGH | RR 0.97, 95% CI 0.94- 1.01,  P = 0.14, I^2^= 10% | There is no significant difference in 1-year mortality for patients undergoing IFI compared to ENI |
|  | RCT | | | | | | | |
| 842  (6 studies) | not  serious^1^ | not  serious^3^ | not  serious^4^ | not  serious | none | ⊕⊕⊕⊕  HIGH | RR 0.98, 95% CI 0.92–1.04,  P = 0.50, I^2^= 0% | There is no significant difference in 1-year mortality for patients in RCT studies undergoing IFI compared to ENI |
|  | non-RCT | | | | | | | |
| 2703  (12 studies) | not  serious^1^ | not  serious^3^ | not  serious^4^ | not  serious | none | ⊕⊕⊕⊕  HIGH | RR 0.97, 95% CI 0.93–1.01,  P = 0.20, I^2^= 33% | There is no significant difference in 1-year mortality for patients in non-RCT studies undergoing IFI compared to ENI |
|  | 3D-CRT | | | | | | | |
| 631  (5 studies) | not  serious^1^ | not  serious^3^ | not  serious^4^ | not  serious | none | ⊕⊕⊕⊕  HIGH | RR 1.03, 95% CI 0.94–1.12,  P = 0.57, I^2^= 0% | There is no significant difference in 1-year mortality for patients who received 3D-CRT undergoing IFI compared to ENI |
|  | IMRT | | | | | | | |
| 1220  (3 studies) | not  serious^1^ | not  serious^3^ | not  serious^4^ | not  serious | none | ⊕⊕⊕⊕  HIGH | RR 0.96, 95% CI 0.90–1.02,  P = 0.17, I^2^= 0% | There is no significant difference in 1-year mortality for patients who received IMRT undergoing IFI compared to ENI |
|  | 3D+IMRT mixed | | | | | | | |
| 886  (6 studies) | not  serious^1^ | not  serious^3^ | not  serious^4^ | not  serious | none | ⊕⊕⊕⊕  HIGH | RR=0.96, 95% CI 0.89–1.03, P = 0.22, I^2^= 27% | There is no significant difference in 1-year mortality for patients who received 3D+ IMRT mixed undergoing IFI compared to ENI |
|  | ESCC | | | | | | | |
| 2332  (13 studies) | not  serious^1^ | not  serious^3^ | not  serious^4^ | not  serious | none | ⊕⊕⊕⊕  HIGH | RR 0.97, 95% CI 0.93–1.00,  P= 0.08, I^2^= 42% | There is no significant difference in 1-year mortality for ESCC patients undergoing IFI compared to ENI |
|  | ESCC mixed | | | | | | | |
| 1213  (5 studies) | not  serious^1^ | not  serious^3^ | not  serious^4^ | not  serious | none | ⊕⊕⊕⊕  HIGH | RR 0.99, 95% CI 0.93–1.06,  P = 0.82, I^2^= 0% | There is no significant difference in 1-year mortality for ESCC mixed patients undergoing IFI compared to ENI |
|  | CCRT | | | | | | | |
| 391  (3 studies) | not  serious^1^ | not  serious^3^ | not  serious^4^ | not  serious | none | ⊕⊕⊕⊕  HIGH | RR 0.94, 95% CI 0.85–1.05,  P = 0.27, I^2^= 0% | There is no significant difference in 1-year mortality for patients who received CCRT undergoing IFI compared to ENI |
|  | CCRT+CT | | | | | | | |
| 811  (5 studies) | not  serious^1^ | not  serious^3^ | not  serious^4^ | not  serious | none | ⊕⊕⊕⊕  HIGH | RR 1.02, 95% CI 0.95–1.09,  P = 0.66, I^2^= 0% | There is no significant difference in 1-year mortality for patients who received CCRT+ CT undergoing IFI compared to ENI |
|  | CRT mixed | | | | | | | |
| 2343  (10 studies) | not  serious^1^ | not  serious^3^ | not  serious^4^ | not  serious | none | ⊕⊕⊕⊕  HIGH | RR 0.97, 95% CI 0.92–1.01,  P = 0.11, I^2^= 49% | There is no significant difference in 1-year mortality for patients who received CRT mixed undergoing IFI compared to ENI |
| Overall Survival at 2-Year | | | | | | | | |
| 2519  (15 studies) | not  serious^1^ | not  serious^3^ | not  serious^4^ | not  serious | none | ⊕⊕⊕⊕  HIGH | RR 0.99, 95% CI 0.92–1.05,  P = 0.70, I^2^= 41% | There is no significant difference in 2-year mortality for patients undergoing IFI compared to ENI |
|  | RCT | | | | | | | |
| 842  (6 studies) | not  serious^1^ | not  serious^3^ | not  serious^4^ | not  serious | none | ⊕⊕⊕⊕  HIGH | RR 0.99, 95% CI 0.89–1.10,  P = 0.84, I^2^= 0% | There is no significant difference in 2-year mortality for patients in RCT studies undergoing IFI compared to ENI |
|  | non-RCT | | | | | | | |
| 1677  (9 studies) | not  serious^1^ | serious^2^ | not  serious^4^ | not  serious | none | ⊕⊕⊕O  MODERATE | RR 0.99, 95% CI 0.86–1.13,  P = 0.84, I^2^= 59% | There is no significant difference in 2-year mortality for patients in non-RCT studies undergoing IFI compared to ENI |
|  | 3D-CRT | | | | | | | |
| 508  (4 studies) | not  serious^1^ | not  serious^3^ | not  serious^4^ | not  serious | none | ⊕⊕⊕⊕  HIGH | RR 1.11, 95% CI 0.96–1.27,  P = 0.16, I^2^= 37% | There is no significant difference in 2-year mortality for patients who received 3D-CRT undergoing IFI compared to ENI |
|  | IMRT | | | | | | | |
| 672  (2 studies) | not  serious^1^ | not  serious^3^ | not  serious^4^ | not  serious | none | ⊕⊕⊕⊕  HIGH | RR 0.96, 95% CI 0.83–1.11,  P = 0.62, I^2^= 40% | There is no significant difference in 2-year mortality for patients who received IMRT undergoing IFI compared to ENI |
|  | 3D+IMRT mixed | | | | | | | |
| 886  (6 studies) | not  serious^1^ | not  serious^3^ | not  serious^4^ | not  serious | none | ⊕⊕⊕⊕  HIGH | RR 0.89, 95% CI 0.75–1.05,  P = 0.17, I^2^= 0% | There is no significant difference in 2-year mortality for patients who received 3D+ IMRT mixed undergoing IFI compared to ENI |
|  | ESCC | | | | | | | |
| 1850  (11 studies) | not  serious^1^ | serious^2^ | not  serious^4^ | not  serious | none | ⊕⊕⊕O  MODERATE | RR=0.99, 95% CI 0.90–1.10,  P = 0.88, I^2^= 51% | There is no significant difference in 2-year mortality for ESCC patients undergoing IFI compared to ENI |
|  | ESCC mixed | | | | | | | |
| 669  (4 studies) | not  serious^1^ | not  serious^3^ | not  serious^4^ | not  serious | none | ⊕⊕⊕⊕  HIGH | RR 1.03, 95% CI 0.88–1.19,  P = 0.74, I^2^= 42% | There is no significant difference in 2-year mortality for ESCC mixed patients undergoing IFI compared to ENI |
|  | CCRT | | | | | | | |
| 391  (3 studies) | not  serious^1^ | not  serious^3^ | not  serious^4^ | somewhat  serious^6^ | none | ⊕⊕⊕O  MODERATE | RR 0.94, 95% CI 0.78–1.14,  P = 0.54, I^2^= 0% | There is no significant difference in 2-year mortality for patients who received CCRT undergoing IFI compared to ENI |
|  | CCRT+CT | | | | | | | |
| 811  (5 studies) | not  serious^1^ | not  serious^3^ | not  serious^4^ | not  serious | none | ⊕⊕⊕⊕  HIGH | RR 1.07, 95% CI 0.96–1.19,  P = 0.22, I^2^= 21% | There is no significant difference in 2-year mortality for patients who received CCRT+ CT undergoing IFI compared to ENI |
|  | CRT mixed | | | | | | | |
| 1317  (7 studies) | not  serious^1^ | serious^2^ | not  serious^4^ | not  serious | none | ⊕⊕⊕O  MODERATE | RR 0.93, 95% CI 0.78–1.13,  P = 0.47, I^2^= 72% | There is no significant difference in 2-year mortality for patients who received CRT mixed undergoing IFI compared to ENI |
| Overall Survival at 3-Year | | | | | | | | |
| 2603  (17 studies) | not  serious^1^ | not  serious^3^ | not  serious^4^ | not  serious | reporting bias^7^ | ⊕⊕⊕O  MODERATE | RR 0.93, 95% CI 0.85–1.02,  P = 0.15, I^2^= 31% | There is no significant difference in 3-year mortality for patients undergoing IFI compared to ENI |
|  | RCT | | | | | | | |
| 842  (6 studies) | not  serious^1^ | not  serious^3^ | not  serious^4^ | not  serious | none | ⊕⊕⊕⊕  HIGH | RR 0.92, 95% CI 0.78–1.08,  P = 0.31, I^2^= 31% | There is no significant difference in 3-year mortality for patients in RCT studies undergoing IFI compared to ENI |
|  | non-RCT | | | | | | | |
| 2971  (14 studies) | not  serious^1^ | serious^2^ | not  serious^4^ | not  serious | none | ⊕⊕⊕O  MODERATE | RR 0.91, 95% CI 0.76–1.09,  P = 0.30, I^2^= 78% | There is no significant difference in 3-year mortality for patients in non-RCT studies undergoing IFI compared to ENI |
|  | 3D-CRT | | | | | | | |
| 730  (6 studies) | not  serious^1^ | not  serious^3^ | not  serious^4^ | not  serious | none | ⊕⊕⊕⊕  HIGH | RR 1.05, 95% CI 0.88–1.26,  P = 0.58, I^2^= 48% | There is no significant difference in 3-year mortality for patients who received 3D-CRT undergoing IFI compared to ENI |
|  | IMRT | | | | | | | |
| 1220  (3 studies) | not  serious^1^ | serious^2^ | not  serious^4^ | not  serious | none | ⊕⊕⊕O  MODERATE | RR 0.83, 95% CI 0.66–1.05,  P = 0.13, I^2^= 60% | There is no significant difference in 3-year mortality for patients who received IMRT undergoing IFI compared to ENI |
|  | 3D+IMRT mixed | | | | | | | |
| 886  (6 studies) | not  serious^1^ | not  serious^3^ | not  serious^4^ | not  serious | none | ⊕⊕⊕⊕  HIGH | RR 0.85, 95% CI 0.71–1.02,  P = 0.07, I^2^= 0% | There is no significant difference in 3-year mortality for patients who received 3D+ IMRT mixed undergoing IFI compared to ENI |
|  | ESCC | | | | | | | |
| 2600  (15 studies) | not  serious^1^ | serious^2^ | not  serious^4^ | not  serious | none | ⊕⊕⊕O  MODERATE | RR 0.94, 95% CI 0.81–1.09, P = 0.44, I^2^= 63% | There is no significant difference in 3-year mortality for ESCC patients undergoing IFI compared to ENI |
|  | ESCC mixed | | | | | | | |
| 1213  (5 studies) | not  serious^1^ | serious^2^ | not  serious^4^ | not  serious | none | ⊕⊕⊕O  MODERATE | RR 0.81, 95% CI 0.58–1.14,  P = 0.24, I^2^= 78% | There is no significant difference in 3-year mortality for ESCC mixed patients undergoing IFI compared to ENI |
|  | CCRT | | | | | | | |
| 391  (3 studies) | not  serious^1^ | not  serious^3^ | not  serious^4^ | somewhat  serious^6^ | none | ⊕⊕⊕O  MODERATE | RR 0.79, 95% CI 0.62–1.00,  P = 0.05, I^2^= 0% | There is no significant difference in 3-year mortality for patients who received CCRT undergoing IFI compared to ENI |
|  | CCRT+CT | | | | | | | |
| 910  (6 studies) | not  serious^1^ | not  serious^3^ | not  serious^4^ | not  serious | none | ⊕⊕⊕⊕  HIGH | RR 1.08, 95% CI 0.93–1.25,  P = 0.32, I^2^= 48% | There is no significant difference in 3-year mortality for patients who received CCRT+ CT undergoing IFI compared to ENI |
|  | CRT mixed | | | | | | | |
| 2512  (11 studies) | not  serious^1^ | serious^2^ | not  serious^4^ | not  serious | none | ⊕⊕⊕O  MODERATE | RR 0.88, 95% CI 0.70–1.10,  P = 0.25, I^2^= 83% | There is no significant difference in 3-year mortality for patients who received CRT mixed undergoing IFI compared to ENI |
| Overall Survival at 5-Year | | | | | | | | |
| 2763  (12 studies) | not  serious^1^ | not  serious^3^ | not  serious^4^ | not  serious | none | ⊕⊕⊕⊕  HIGH | RR 0.78, 95% CI 0.68–0.90,  P = 0.0004, I^2^= 14% | IFI decreases mortality by 22% more than ENI at 5-year |
|  | IMRT | | | | | | | |
| 1220  (3 studies) | not  serious^1^ | not  serious^3^ | not  serious^4^ | not  serious | none | ⊕⊕⊕⊕  HIGH | RR 0.77, 95% CI 0.63–0.95,  P = 0.01, I^2^= 6% | IFI decreases mortality by 23% more than ENI at 5-year for patients receiving IMRT |
|  | 3D+IMRT mixed | | | | | | | |
| 807  (5 studies) | not  serious^1^ | not  serious^3^ | not  serious^4^ | not  serious | none | ⊕⊕⊕⊕  HIGH | RR 0.84, 95% CI 0.64–1.10,  P = 0.21, I^2^= 34% | There is no significant difference in 5-year mortality for patients who received 3D+ IMRT mixed undergoing IFI compared to ENI |
|  | ESCC | | | | | | | |
| 1789  (8 studies) | not  serious^1^ | not  serious^3^ | not  serious^4^ | not  serious | none | ⊕⊕⊕⊕  HIGH | RR 0.84, 95% CI 0.71–0.99,  P = 0.04, I^2^= 30% | IFI decreases mortality by 16% more than ENI at 5-year in ESCC patients |
|  | ESCC mixed | | | | | | | |
| 974  (4 studies) | not  serious^1^ | not  serious^3^ | not  serious^4^ | not  serious | none | ⊕⊕⊕⊕  HIGH | RR 0.68, 95% CI 0.53–0.86,  P = 0.002, I^2^= 0% | IFI decreases mortality by 32% more than ENI at 5-year in ESCC mixed patients |
| ≥ grade 2 acute esophagitis | | | | | | | | |
| 1845  (9 studies) | not  serious^1^ | not  serious^3^ | not  serious^4^ | not  serious | none | ⊕⊕⊕⊕  HIGH | RR 0.79, 95% CI 0.68–0.91,  P = 0.001, I^2^= 13% | IFI decreases the incidence ≥ grade 2 AE by 21% more than ENI |
|  | RCT | | | | | | | |
| 474  (4 studies) | not  serious^1^ | not  serious^3^ | not  serious^4^ | not  serious | none | ⊕⊕⊕⊕  HIGH | RR 0.70, 95% CI 0.56–0.87,  P = 0.001, I^2^= 0% | IFI decreases the incidence ≥ grade 2 AE by 30% more than ENI in RCT studies |
|  | non-RCT | | | | | | | |
| 1371  (5 studies) | not  serious^1^ | not  serious^3^ | not  serious^4^ | not  serious | none | ⊕⊕⊕⊕  HIGH | RR 0.84, 95% CI 0.71–0.99,  P = 0.04, I^2^= 29% | IFI decreases the incidence ≥ grade 2 AE by 16% more than ENI in non-RCT studies |
|  | 3D-CRT | | | | | | | |
| 392  (4 studies) | not  serious^1^ | not  serious^3^ | not  serious^4^ | somewhat  serious^6^ | none | ⊕⊕⊕O  MODERATE | RR 0.80, 95% CI 0.64–1.00,  P = 0.05, I^2^= 0% | IFI decreases the incidence ≥ grade 2 AE by 20% more than ENI for patients who received 3D-CRT |
|  | IMRT | | | | | | | |
| 1220  (3 studies) | not  serious^1^ | not  serious^3^ | not  serious^4^ | not  serious | none | ⊕⊕⊕⊕  HIGH | RR 0.83, 95% CI 0.70–0.99,  P = 0.04, I^2^= 50% | IFI decreases the incidence ≥ grade 2 AE by 17% more than ENI for patients who received IMRT |
| ≥ grade 2 acute pneumonia | | | | | | | | |
| 1777  (8 studies) | not  serious^1^ | not  serious^3^ | not  serious^4^ | not  serious | none | ⊕⊕⊕⊕  HIGH | RR 0.83, 95% CI 0.66–1.04,  P = 0.10, I^2^= 33% | There is no significant difference in ≥ grade 2 AP for patients undergoing IFI compared to ENI |
|  | RCT | | | | | | | |
| 666  (5 studies) | not  serious^1^ | not  serious^3^ | not  serious^4^ | not  serious | none | ⊕⊕⊕⊕  HIGH | RR 0.69, 95% CI 0.51–0.93,  P = 0.02, I^2^= 36% | IFI decreases the incidence ≥ grade 2 AP by 31% more than ENI for patients in RCT studies |
|  | non-RCT | | | | | | | |
| 1234  (4 studies) | not  serious^1^ | serious^2^ | not  serious^4^ | not  serious | none | ⊕⊕⊕O  MODERATE | RR 0.71, 95% CI 0.35–1.45,  P = 0.35, I^2^= 79% | There is no significant difference in ≥ grade 2 AP for patients in non-RCT studies undergoing IFI compared to ENI |
|  | 3D-CRT | | | | | | | |
| 392  (4 studies) | not  serious^1^ | serious^2^ | not  serious^4^ | somewhat  serious^6^ | none | ⊕⊕OO  LOW | RR 0.65, 95% CI 0.35–1.21,  P = 0.17, I^2^= 67% | There is no significant difference in ≥ grade 2 AP for patients who received 3D-CRT undergoing IFI compared to ENI |
|  | IMRT | | | | | | | |
| 1220  (3 studies) | not  serious^1^ | serious^2^ | not  serious^4^ | not  serious | none | ⊕⊕⊕O  MODERATE | RR 0.84, 95% CI 0.52–1.35,  P = 0.47, I^2^= 55% | There is no significant difference in ≥ grade 2 AP for patients who received IMRT undergoing IFI compared to ENI |
| ≥ grade 3 acute esophagitis | | | | | | | | |
| 2219  (11 studies) | not  serious^1^ | not  serious^3^ | not  serious^4^ | not  serious | none | ⊕⊕⊕⊕  HIGH | RR 0.51, 95% CI 0.38–0.69,  P < 0.00001, I^2^= 14% | IFI decreases the incidence ≥ grade 3 AE by 49% more than ENI |
|  | RCT | | | | | | | |
| 474  (4 studies) | not  serious^1^ | not  serious^3^ | not  serious^4^ | not  serious | none | ⊕⊕⊕⊕  HIGH | RR 0.39, 95% CI 0.24–0.64,  P = 0.0002, I^2^= 0% | IFI decreases the incidence ≥ grade 3 AE by 61% more than ENI for patients in RCT studies |
|  | non-RCT | | | | | | | |
| 1745  (7 studies) | not  serious^1^ | not  serious^3^ | not  serious^4^ | not  serious | none | ⊕⊕⊕⊕  HIGH | RR 0.60, 95% CI 0.41–0.88,  P = 0.008, I^2^= 25% | IFI decreases the incidence ≥ grade 3 AE by 40% more than ENI for patients in non-RCT studies |
|  | 3D-CRT | | | | | | | |
| 508  (4 studies) | not  serious^1^ | not  serious^3^ | not  serious^4^ | not  serious | none | ⊕⊕⊕⊕  HIGH | RR 0.46, 95% CI 0.27–0.80,  P = 0.006, I^2^= 0% | IFI decreases the incidence ≥ grade 3 AE by 54% more than ENI for patients who received 3D-CRT |
|  | IMRT | | | | | | | |
| 1220  (3 studies) | not  serious^1^ | not  serious^3^ | not  serious^4^ | not  serious | none | ⊕⊕⊕⊕  HIGH | RR 0.66, 95% CI 0.41–1.05,  P = 0.08, I^2^= 45% | There is no significant difference in ≥ grade 3 AE for patients who received IMRT undergoing IFI compared to ENI |
|  | 3D+IMRT mixed | | | | | | | |
| 322  (3 studies) | not  serious^1^ | not  serious^3^ | not  serious^4^ | somewhat  serious^6^ | none | ⊕⊕⊕O  MODERATE | RR 0.31, 95% CI 0.16–0.59,  P = 0.0003, I^2^= 0% | IFI decreases the incidence ≥ grade 3 AE by 69% more than ENI for patients who received 3D+ IMRT mixed |
|  | ESCC | | | | | | | |
| 1251  (7 studies) | not  serious^1^ | not  serious^3^ | not  serious^4^ | not  serious | none | ⊕⊕⊕⊕  HIGH | RR 0.50, 95% CI 0.34–0.72,  P = 0.0002, I^2^= 15% | IFI decreases the incidence ≥ grade 3 AE by 50% more than ENI in ESCC patients |
|  | ESCC mixed | | | | | | | |
| 968  (4 studies) | not  serious^1^ | not  serious^3^ | not  serious^4^ | not  serious | none | ⊕⊕⊕⊕  HIGH | RR 0.54, 95% CI 0.33–0.89,  P = 0.02, I^2^= 32% | IFI decreases the incidence ≥ grade 3 AE by 46% more than ENI in ESCC mixed patients |
| ≥ grade 3 acute pneumonia | | | | | | | | |
| 2130  (10 studies) | not  serious^1^ | not  serious^3^ | not  serious^4^ | not  serious | none | ⊕⊕⊕⊕  HIGH | RR 0.95, 95% CI 0.63–1.41,  P = 0.78, I^2^= 26% | There is no significant difference in ≥ grade 3 AP for patients undergoing IFI compared to ENI |
|  | RCT | | | | | | | |
| 474  (4 studies) | not  serious^1^ | not  serious^3^ | not  serious^4^ | not  serious | none | ⊕⊕⊕⊕  HIGH | RR 0.56, 95% CI 0.28–1.12,  P = 0.10, I^2^= 0% | There is no significant difference in ≥ grade 3 AP for patients in RCT studies undergoing IFI compared to ENI |
|  | non-RCT | | | | | | | |
| 1112  (5 studies) | not  serious^1^ | not  serious^3^ | not  serious^4^ | not  serious | none | ⊕⊕⊕⊕  HIGH | RR 0.90, 95% CI 0.49–1.66,  P = 0.73, I^2^= 0% | There is no significant difference in ≥ grade 3 AP for patients in non-RCT studies undergoing IFI compared to ENI |
|  | 3D-CRT | | | | | | | |
| 508  (4 studies) | not  serious^1^ | not  serious^3^ | not  serious^4^ | not  serious | none | ⊕⊕⊕⊕  HIGH | RR 0.57, 95% CI 0.27–1.20,  P = 0.14, I^2^= 0% | There is no significant difference in ≥ grade 3 AP for patients who received 3D-CRT undergoing IFI compared to ENI |
|  | IMRT | | | | | | | |
| 1220  (3 studies) | not  serious^1^ | serious^2^ | not  serious^4^ | somewhat  serious^6^ | none | ⊕⊕OO  LOW | RR=1.06, 95% CI 0.41–2.71, P = 0.90, I^2^= 56% | There is no significant difference in ≥ grade 3 AP for patients who received IMRT undergoing IFI compared to ENI |
|  | ESCC | | | | | | | |
| 1251  (7 studies) | not  serious^1^ | not  serious^3^ | not  serious^4^ | not  serious | none | ⊕⊕⊕⊕  HIGH | RR 0.79, 95% CI 0.48–1.29,  P = 0.35, I^2^= 0% | There is no significant difference in ≥ grade 3 AP for ESCC patients undergoing IFI compared to ENI |
|  | ESCC mixed | | | | | | | |
| 879  (3 studies) | not  serious^1^ | serious^2^ | not  serious^4^ | very  serious^5,6^ | none | ⊕OOO  VERY LOW | RR 1.04, 95% CI 0.21–5.01,  P = 0.96, I^2^= 73% | There is no significant difference in ≥ grade 3 AP for ESCC mixed patients undergoing IFI compared to ENI |
| ≥ grade 3 late esophagitis | | | | | | | | |
| 682  (5 studies) | not  serious^1^ | not  serious^3^ | not  serious^4^ | not  serious | none | ⊕⊕⊕⊕  HIGH | RR 0.92, 95% CI 0.47–1.80,  P= 0.81, I^2^= 0% | There is no significant difference in ≥ grade 3 LE for patients undergoing IFI compared to ENI |
| ≥ grade 3 late pneumonia | | | | | | | | |
| 682  (5 studies) | not  serious^1^ | not  serious^3^ | not  serious^4^ | not  serious | none | ⊕⊕⊕⊕  HIGH | RR 0.78, 95% CI 0.42–1.42,  P = 0.41, I^2^= 39% | There is no significant difference in ≥ grade 3 LP for patients undergoing IFI compared to ENI |
| Profession Free Survival at 1-Year | | | | | | | | |
| 2349  (12 studies) | not  serious^1^ | not  serious^3^ | not  serious^4^ | not  serious | none | ⊕⊕⊕⊕  HIGH | RR 0.90, 95% CI 0.85–0.95,  P = 0.0004, I^2^= 45% | IFI decreases PFS rate by 10% more than ENI at 1-year |
| Profession Free Survival at 2-Year | | | | | | | | |
| 1949  (11 studies) | not  serious^1^ | not  serious^3^ | not  serious^4^ | not  serious | none | ⊕⊕⊕⊕  HIGH | RR 0.92, 95% CI 0.84–1.01,  P = 0.09, I^2^= 0% | There is no significant difference in 2-year PFS for patients undergoing IFI compared to ENI |
| Profession Free Survival at 3-Year | | | | | | | | |
| 2493  (12 studies) | not  serious^1^ | not  serious^3^ | not  serious^4^ | not  serious | reporting bias^7^ | ⊕⊕⊕O  MODERATE | RR 0.86, 95% CI 0.78–0.96,  P = 0.007, I^2^= 49% | IFI decreases PFS rate by 14% more than ENI at 3-year |
| Profession Free Survival at 5-Year | | | | | | | | |
| 2587  (11 studies) | not  serious^1^ | not  serious^3^ | not  serious^4^ | not  serious | none | ⊕⊕⊕⊕  HIGH | RR 0.81, 95% CI 0.70–0.93,  P = 0.003, I^2^= 5% | IFI decreases PFS rate by 19% more than ENI at 5-year |
| Local Control at 1-Year | | | | | | | | |
| 610  (5 studies) | not  serious^1^ | not  serious^3^ | not  serious^4^ | not  serious | none | ⊕⊕⊕⊕  HIGH | RR 0.94, 95% CI 0.85–1.02,  P = 0.15, I^2^= 39% | There is no significant difference in 1-year local control for patients undergoing IFI compared to ENI |
| Local Control at 2-Year | | | | | | | | |
| 365  (4 studies) | not  serious^1^ | not  serious^3^ | not  serious^4^ | somewhat  serious^6^ | none | ⊕⊕⊕O  MODERATE | RR 0.87, 95% CI 0.77–0.99,  P = 0.04, I^2^= 0% | IFI decreases LCR by 13% more than ENI at 2-year |
| Local Control at 3-Year | | | | | | | | |
| 610  (5 studies) | not  serious^1^ | not  serious^3^ | not  serious^4^ | not  serious | none | ⊕⊕⊕⊕  HIGH | RR 0.87, 95% CI 0.76–1.00,  P = 0.04, I^2^= 7% | IFI decreases LCR by 13% more than ENI at 3-year |

1. While the majority of studies were non-RCTs, all included studies demonstrated a low risk of selection bias, reporting bias and confirmation bias.

2. Quality of evidence was downgraded because high heterogeneity (I2>50%) was present in these meta-analyzed outcomes.

3. Low heterogeneity, I2≤50% with similar point estimates and overlapping confidence intervals.

4. The majority of included studies directly compare involved field irradiation (IFI) to elective nodal irradiation (ENI) in relevant patients and report common outcomes of interest.

5. Downgraded one point because of large confidence intervals that overlaps no effect, as well as effects favoring both involved field irradiation (IFI) and elective nodal irradiation (ENI), suggesting that more studies with larger sample sizes are needed.

6. Downgraded one point because the total number of events was less than 300 or the total number of sample size was less than 400.

7. Downgraded one point because of publication bias.

**Abbreviations:** IFI, involved field irradiation; ENI, elective nodal irradiation; RR, risk ratio; M-H, Mantel-Haenszel; CI, confidence interval; 3D-CRT, three-dimensional conformal radiotherapy; IMRT, intensity modulated radiotherapy; 3D+IMRT mixed, both 3D-CRT and IMRT; ESCC, esophageal squamous cell carcinoma; ESCC mixed, both ESCC and non-ESCC; CCRT, concurrent chemoradiotherapy; CCRT+CT, concurrent chemoradiotherapy + chemotherapy; CRT, radiotherapy with or without chemotherapy; AE, acute esophagitis; AP, acute pneumonia; LE, late esophagitis; LP, late pneumonia.
