# Supplementary material for: The role of involved field irradiation versus elective nodal irradiation in definitive radiotherapy or chemoradiotherapy for esophageal cancer- a systematic review and meta-analysis
Source: Front Oncol. 2022 Nov 2;12:1034656. doi: 10.3389/fonc.2022.1034656 (PMC9666894; doi:10.3389/fonc.2022.1034656)
Supplement: Supplementary file 1 [file DataSheet_1.zip › supplementary materials/titles and abbreviations of supplementary material.docx]

**Supplementary Material**

**Supplementary Figure**

**Supplementary Figure. 1** Subgroup analysis of 1-year overall survival separated by (A) RCT and (B) non-RCT

**Abbreviations:** IFI, involved field irradiation; ENI, elective nodal irradiation; M-H, Mantel-Haenszel; CI, confidence interval.

**Supplementary Figure. 2** Subgroup analysis of 1-year overall survival separated by (A) 3D-CRT, (B) IMRT, and (C) 3D+IMRT mixed

**Abbreviations:** IFI, involved field irradiation; ENI, elective nodal irradiation; M-H, Mantel-Haenszel; CI, confidence interval; 3D-CRT, three-dimensional conformal radiotherapy; IMRT, intensity modulated radiotherapy; 3D+IMRT mixed, both 3D-CRT and IMRT.

**Supplementary Figure. 3** Subgroup analysis of 1-year overall survival separated by (A) ESCC and (B) ESCC mixed

**Abbreviations:** IFI, involved field irradiation; ENI, elective nodal irradiation; M-H, Mantel-Haenszel; CI, confidence interval; ESCC, esophageal squamous cell carcinoma; ESCC mixed, both ESCC and non-ESCC.

**Supplementary Figure. 4** Subgroup analysis of 1-year overall survival separated by (A) CCRT, (B) CCRT+CT, and (C) CRT mixed

**Abbreviations:** IFI, involved field irradiation; ENI, elective nodal irradiation; M-H, Mantel-Haenszel; CI, confidence interval; CCRT, concurrent chemoradiotherapy; CCRT+CT, concurrent chemoradiotherapy + chemotherapy; CRT, radiotherapy with or without chemotherapy.

**Supplementary Figure. 5** Subgroup analysis of 2-year overall survival separated by (A) RCT and (B) non-RCT

**Abbreviations:** IFI, involved field irradiation; ENI, elective nodal irradiation; M-H, Mantel-Haenszel; CI, confidence interval.

**Supplementary Figure. 6** Subgroup analysis of 2-year overall survival separated by (A) 3D-CRT, (B) IMRT, and (C) 3D+IMRT mixed

**Abbreviations:** IFI, involved field irradiation; ENI, elective nodal irradiation; M-H, Mantel-Haenszel; CI, confidence interval; 3D-CRT, three-dimensional conformal radiotherapy; IMRT, intensity modulated radiotherapy; 3D+IMRT mixed, both 3D-CRT and IMRT.

**Supplementary Figure. 7** Subgroup analysis of 2-year overall survival separated by (A) ESCC and (B) ESCC mixed

**Abbreviations:** IFI, involved field irradiation; ENI, elective nodal irradiation; M-H, Mantel-Haenszel; CI, confidence interval; ESCC, esophageal squamous cell carcinoma; ESCC mixed, both ESCC and non-ESCC.

**Supplementary Figure. 8** Subgroup analysis of 2-year overall survival separated by (A) CCRT, (B) CCRT+CT, and (C) CRT mixed

**Abbreviations:** IFI, involved field irradiation; ENI, elective nodal irradiation; M-H, Mantel-Haenszel; CI, confidence interval; CCRT, concurrent chemoradiotherapy; CCRT+CT, concurrent chemoradiotherapy + chemotherapy; CRT, radiotherapy with or without chemotherapy.

**Supplementary Figure. 9** Subgroup analysis of 3-year overall survival separated by (A) RCT and (B) non-RCT

**Abbreviations:** IFI, involved field irradiation; ENI, elective nodal irradiation; M-H, Mantel-Haenszel; CI, confidence interval.

**Supplementary Figure. 10** Subgroup analysis of 3-year overall survival separated by (A) 3D-CRT, (B) IMRT, and (C) 3D+IMRT mixed

**Abbreviations:** IFI, involved field irradiation; ENI, elective nodal irradiation; M-H, Mantel-Haenszel; CI, confidence interval; 3D-CRT, three-dimensional conformal radiotherapy; IMRT, intensity modulated radiotherapy; 3D+IMRT mixed, both 3D-CRT and IMRT.

**Supplementary Figure. 11** Subgroup analysis of 3-year overall survival separated by (A) ESCC and (B) ESCC mixed

**Abbreviations:** IFI, involved field irradiation; ENI, elective nodal irradiation; M-H, Mantel-Haenszel; CI, confidence interval; ESCC, esophageal squamous cell carcinoma; ESCC mixed, both ESCC and non-ESCC.

**Supplementary Figure. 12** Subgroup analysis of 3-year overall survival separated by (A) CCRT, (B) CCRT+CT, and (C) CRT mixed

**Abbreviations:** IFI, involved field irradiation; ENI, elective nodal irradiation; M-H, Mantel-Haenszel; CI, confidence interval; CCRT, concurrent chemoradiotherapy; CCRT+CT, concurrent chemoradiotherapy + chemotherapy; CRT, radiotherapy with or without chemotherapy.

**Supplementary Figure. 13** Subgroup analysis of 5-year overall survival separated by (A) IMRT and (B) 3D+IMRT mixed

**Abbreviations:** IFI, involved field irradiation; ENI, elective nodal irradiation; M-H, Mantel-Haenszel; CI, confidence interval; 3D-CRT, three-dimensional conformal radiotherapy; IMRT, intensity modulated radiotherapy; 3D+IMRT mixed, both 3D-CRT and IMRT.

**Supplementary Figure. 14** Subgroup analysis of 5-year overall survival separated by (A) ESCC and (B) ESCC mixed

**Abbreviations:** IFI, involved field irradiation; ENI, elective nodal irradiation; M-H, Mantel-Haenszel; CI, confidence interval; ESCC, esophageal squamous cell carcinoma; ESCC mixed, both ESCC and non-ESCC.

**Supplementary Figure. 15** Subgroup analysis of ≥ grade 2 AE separated by (A) RCT and (B) non-RCT

**Abbreviations:** IFI, involved field irradiation; ENI, elective nodal irradiation; M-H, Mantel-Haenszel; CI, confidence interval; AE, acute esophagitis.

**Supplementary Figure. 16** Subgroup analysis of ≥ grade 2 AE separated by (A) 3D-CRT and (B) IMRT

**Abbreviations:** IFI, involved field irradiation; ENI, elective nodal irradiation; M-H, Mantel-Haenszel; CI, confidence interval; AE, acute esophagitis; 3D-CRT, three-dimensional conformal radiotherapy; IMRT, intensity modulated radiotherapy.

**Supplementary Figure. 17** Subgroup analysis of ≥ grade 2 AP separated by (A) RCT and (B) non-RCT

**Abbreviations:** IFI, involved field irradiation; ENI, elective nodal irradiation; M-H, Mantel-Haenszel; CI, confidence interval; AP, acute pneumonia.

**Supplementary Figure. 18** Subgroup analysis of ≥ grade 2 AP separated by (A) 3D-CRT and (B) IMRT

**Abbreviations:** IFI, involved field irradiation; ENI, elective nodal irradiation; M-H, Mantel-Haenszel; CI, AP, acute pneumonia; confidence interval; 3D-CRT, three-dimensional conformal radiotherapy; IMRT, intensity modulated radiotherapy.

**Supplementary Figure. 19** Subgroup analysis of ≥ grade 3 AE separated by (A) RCT and (B) non-RCT

**Abbreviations:** IFI, involved field irradiation; ENI, elective nodal irradiation; M-H, Mantel-Haenszel; CI, confidence interval; AE, acute esophagitis.

**Supplementary Figure. 20** Subgroup analysis of ≥ grade 3 AE separated by (A) 3D-CRT, (B) IMRT, and (C) 3D+IMRT mixed

**Abbreviations:** IFI, involved field irradiation; ENI, elective nodal irradiation; M-H, Mantel-Haenszel; CI, confidence interval; AE, acute esophagitis; 3D-CRT, three-dimensional conformal radiotherapy; IMRT, intensity modulated radiotherapy; 3D+IMRT mixed, both 3D-CRT and IMRT.

**Supplementary Figure. 21** Subgroup analysis of ≥ grade 3 AE separated by (A) ESCC and (B) ESCC mixed

**Abbreviations:** IFI, involved field irradiation; ENI, elective nodal irradiation; M-H, Mantel-Haenszel; CI, confidence interval; AE, acute esophagitis; ESCC, esophageal squamous cell carcinoma; ESCC mixed, both ESCC and non-ESCC.

**Supplementary Figure. 22** Subgroup analysis of ≥ grade 3 AP separated by (A) RCT and (B) non-RCT

**Abbreviations:** IFI, involved field irradiation; ENI, elective nodal irradiation; M-H, Mantel-Haenszel; CI, confidence interval; AP, acute pneumonia.

**Supplementary Figure. 23** Subgroup analysis of ≥ grade 3 AP separated by (A) 3D-CRT, and (B) IMRT

**Abbreviations:** IFI, involved field irradiation; ENI, elective nodal irradiation; M-H, Mantel-Haenszel; CI, confidence interval; AP, acute pneumonia; 3D-CRT, three-dimensional conformal radiotherapy; IMRT, intensity modulated radiotherapy.

**Supplementary Figure. 24** Subgroup analysis of ≥ grade 3 AP separated by (A) ESCC and (B) ESCC mixed

**Abbreviations:** IFI, involved field irradiation; ENI, elective nodal irradiation; M-H, Mantel-Haenszel; CI, confidence interval; AP, acute pneumonia; ESCC, esophageal squamous cell carcinoma; ESCC mixed, both ESCC and non-ESCC.

**Supplementary Figure. 25** Funnel Plot of 1-year overall survival of (A) overall studies and (B) non-RCT group

**Supplementary Figure. 26** Funnel Plot of 1-year overall survival of (A) ESCC group and (B) CRT mixed group

**Supplementary Figure. 27** Funnel Plot of 2-year overall survival of (A) overall studies and (B) ESCC group

**Supplementary Figure. 28** Funnel Plot of 3-year overall survival of (A) overall studies and (B) non-RCT group

**Supplementary Figure. 29** Funnel Plot of 3-year overall survival of (A) ESCC group and (B) CRT mixed group

**Supplementary Figure. 30** Funnel Plot of 5-year overall survival of (A) overall studies and (B) non-RCT group

**Supplementary Figure. 31** Funnel Plot of (A) ≥ grade 2 acute esophagitis (AE), (B) ≥ grade 3 acute esophagitis (AE), and (C) ≥ grade 3 acute pneumonia (AP)

**Supplementary Figure. 32** Funnel Plot of (A) 1-year profession free survival and (B) 2-year profession free survival

**Supplementary Figure. 33** Funnel Plot of (A) 3-year profession free survival and (B) 5-year profession free survival

**Supplementary Table**

**Supplementary Table. 1** Search Strategy

**Supplementary Table. 2** Summary of Treatment

**Abbreviations**: IFI, involved field irradiation; ENI, elective nodal irradiation; 2D-RT, two-dimensional radiation therapy; 3D-CRT, three-dimensional conformal radiotherapy; IMRT, intensity modulated radiotherapy; IGRT, image-guided radiation therapy; VMAT, volumetric-modulated arc therapy; RT, radiation therapy; CT, chemotherapy; CRT, chemoradiotherapy; CCRT, concurrent chemoradiotherapy.

**Supplementary Table. 3** Summary of Overall Survival and Profession Free Survival

**Abbreviations:** IFI, involved field irradiation; ENI, elective nodal irradiation; OS, overall survival; PFS, profession free survival.

**Supplementary Table. 4** Summary of Local Contral Rates and Incidence of toxicity related to IFI or ENI

**Abbreviations:** IFI, involved field irradiation; ENI, elective nodal irradiation; LCR, local control rates; AE, acute esophagitis; AP, acute pneumonia; LE, late esophagitis; LP, late pneumonia.

**Supplementary Table. 5** Cochrane Risk of Bias

**Supplementary Table. 6** Newcastle-Ottawa Risk of Bias and AHRQ Standard

**Supplementary Table. 7** Grading of Recommendations, Assessment, Development, and Evaluation (GRADE) of meta-analyzed outcomes

**Abbreviations**: IFI, involved field irradiation; ENI, elective nodal irradiation; RR, risk ratio; M-H, Mantel-Haenszel; CI, confidence interval; 3D-CRT, three-dimensional conformal radiotherapy; IMRT, intensity modulated radiotherapy; 3D+IMRT mixed, both 3D-CRT and IMRT; ESCC, esophageal squamous cell carcinoma; ESCC mixed, both ESCC and non-ESCC; CCRT, concurrent chemoradiotherapy; CCRT+CT, concurrent chemoradiotherapy + chemotherapy; CRT, radiotherapy with or without chemotherapy; AE, acute esophagitis; AP, acute pneumonia; LE, late esophagitis; LP, late pneumonia.
