# Supplementary figures and images for: The role of involved field irradiation versus elective nodal irradiation in definitive radiotherapy or chemoradiotherapy for esophageal cancer- a systematic review and meta-analysis
Source: Front Oncol. 2022 Nov 2;12:1034656. doi: 10.3389/fonc.2022.1034656 (PMC9666894; doi:10.3389/fonc.2022.1034656)

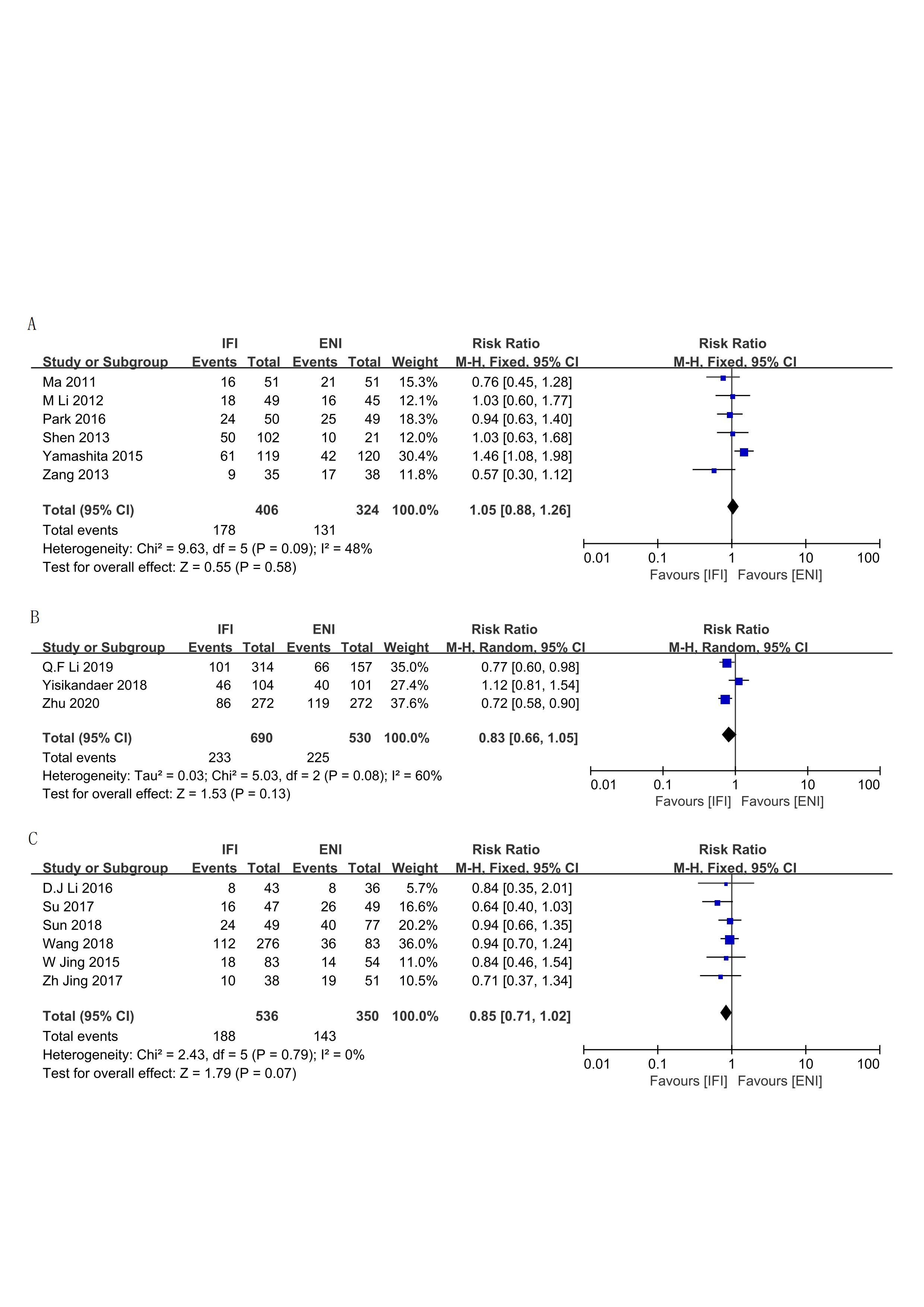

Supplement: Supplementary file 1 [file DataSheet_1.zip › supplementary materials/Supplementary Figure/Supplementary Figure. 10_00.jpg]

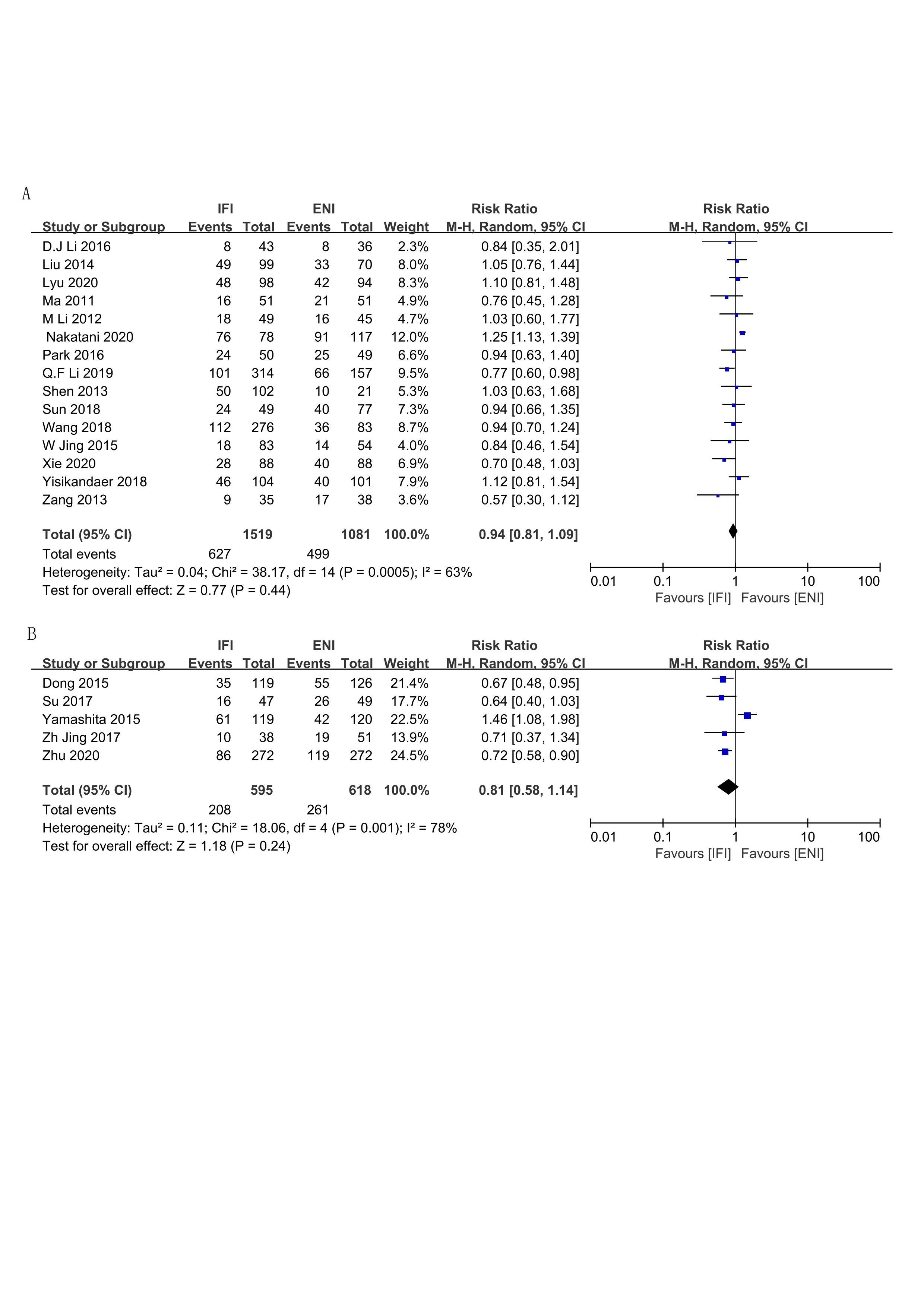

Supplement: Supplementary file 1 [file DataSheet_1.zip › supplementary materials/Supplementary Figure/Supplementary Figure. 11_00.jpg]

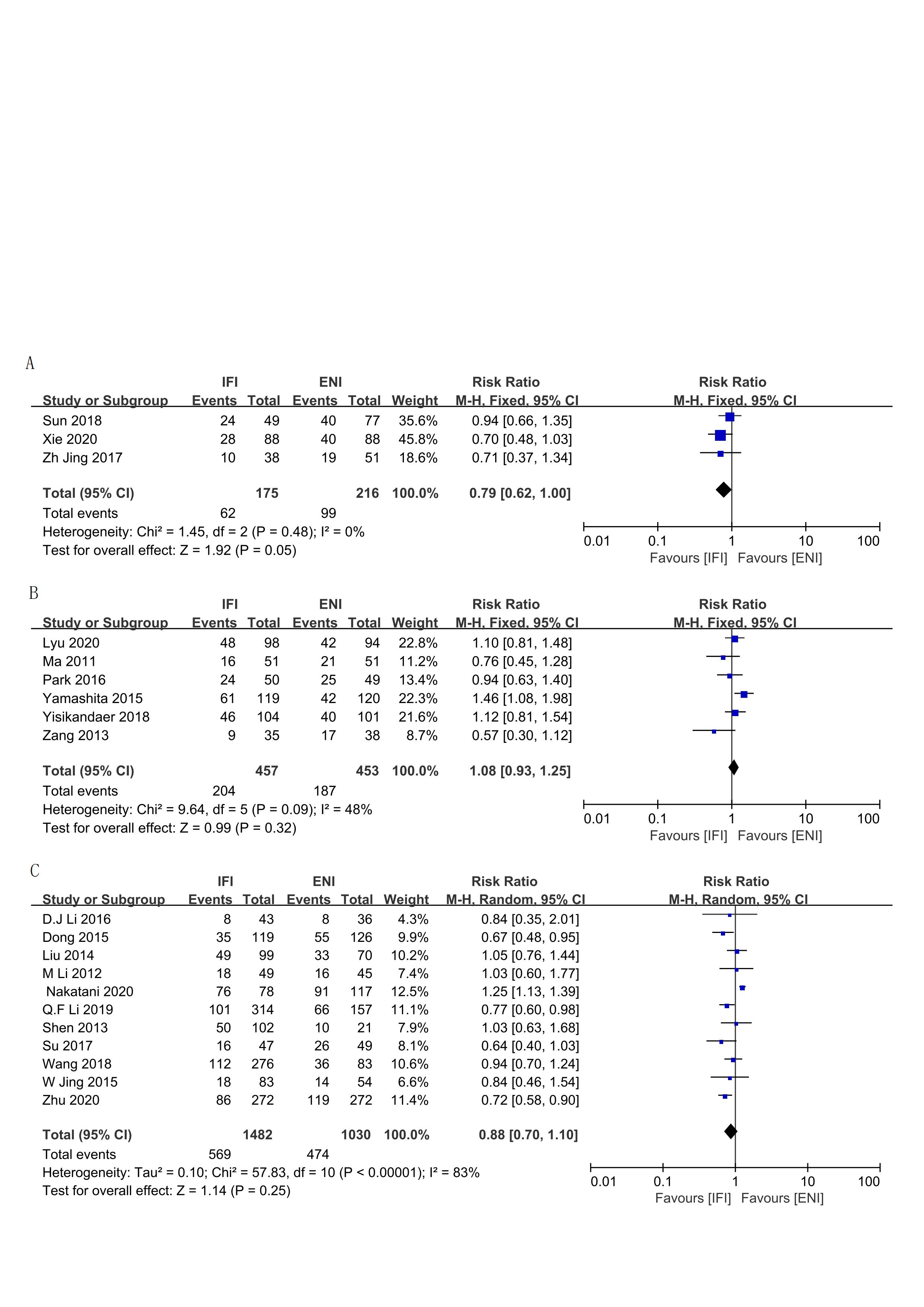

Supplement: Supplementary file 1 [file DataSheet_1.zip › supplementary materials/Supplementary Figure/Supplementary Figure. 12_00.jpg]

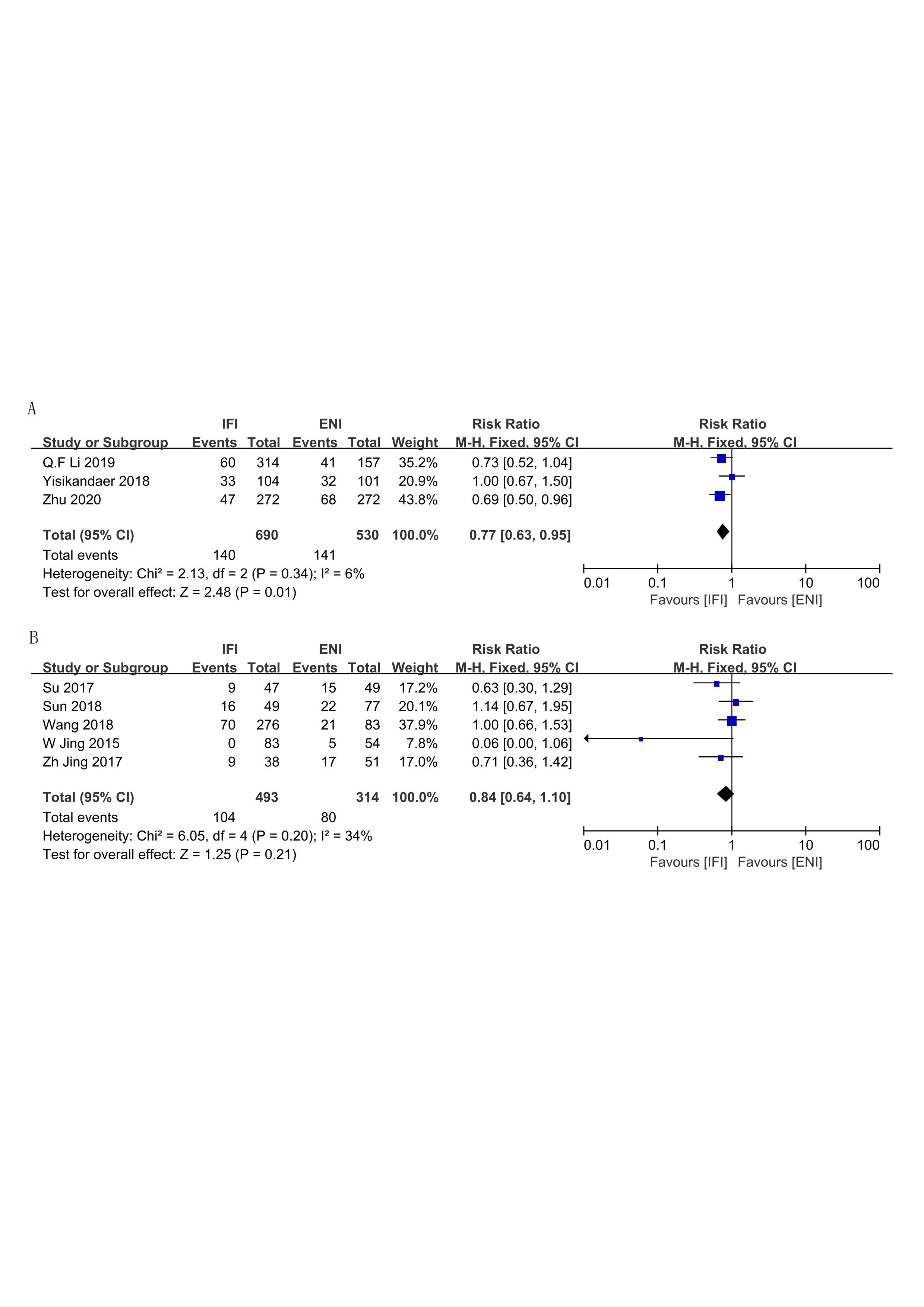

Supplement: Supplementary file 1 [file DataSheet_1.zip › supplementary materials/Supplementary Figure/Supplementary Figure. 13_00.jpg]

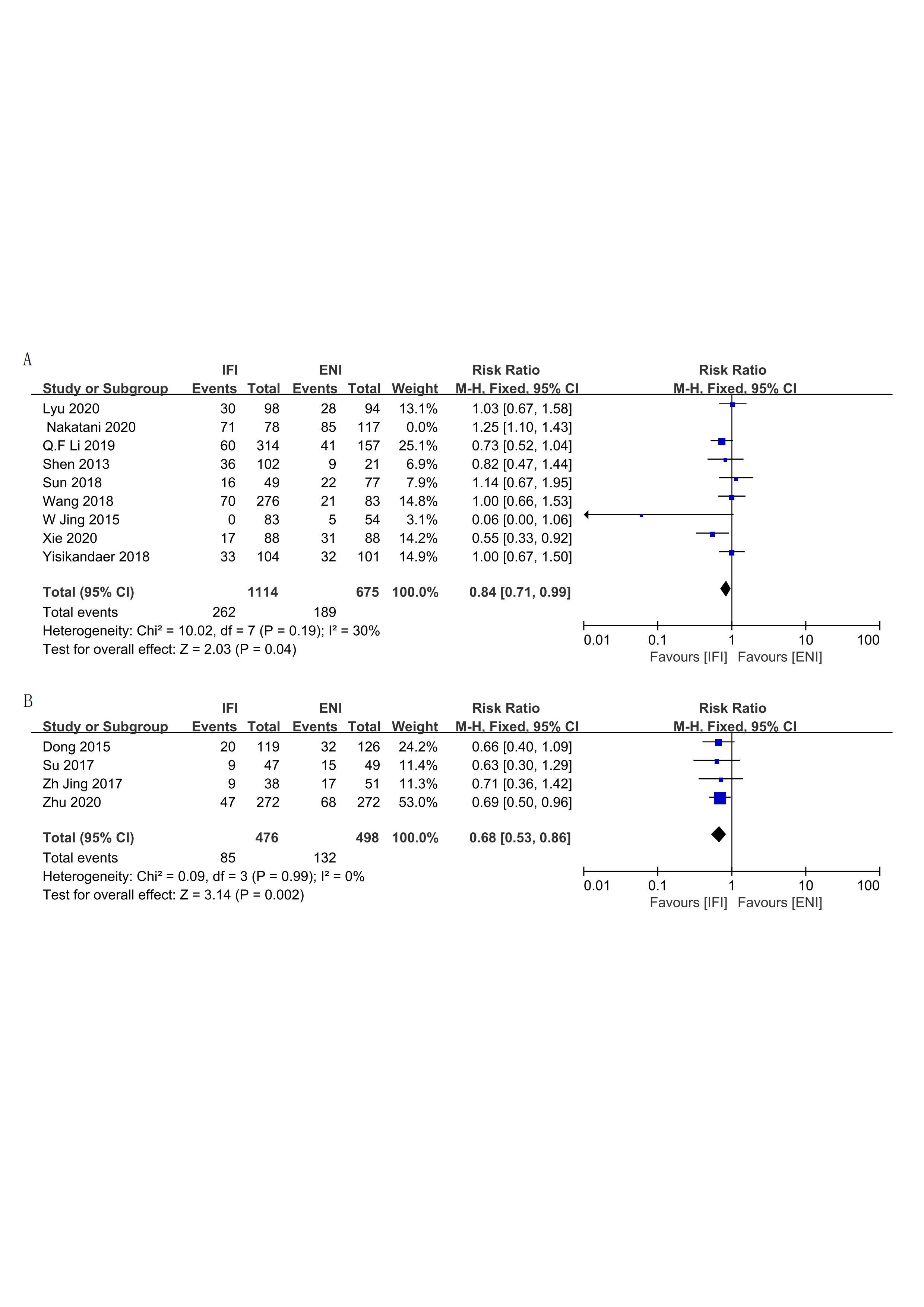

Supplement: Supplementary file 1 [file DataSheet_1.zip › supplementary materials/Supplementary Figure/Supplementary Figure. 14_00.jpg]

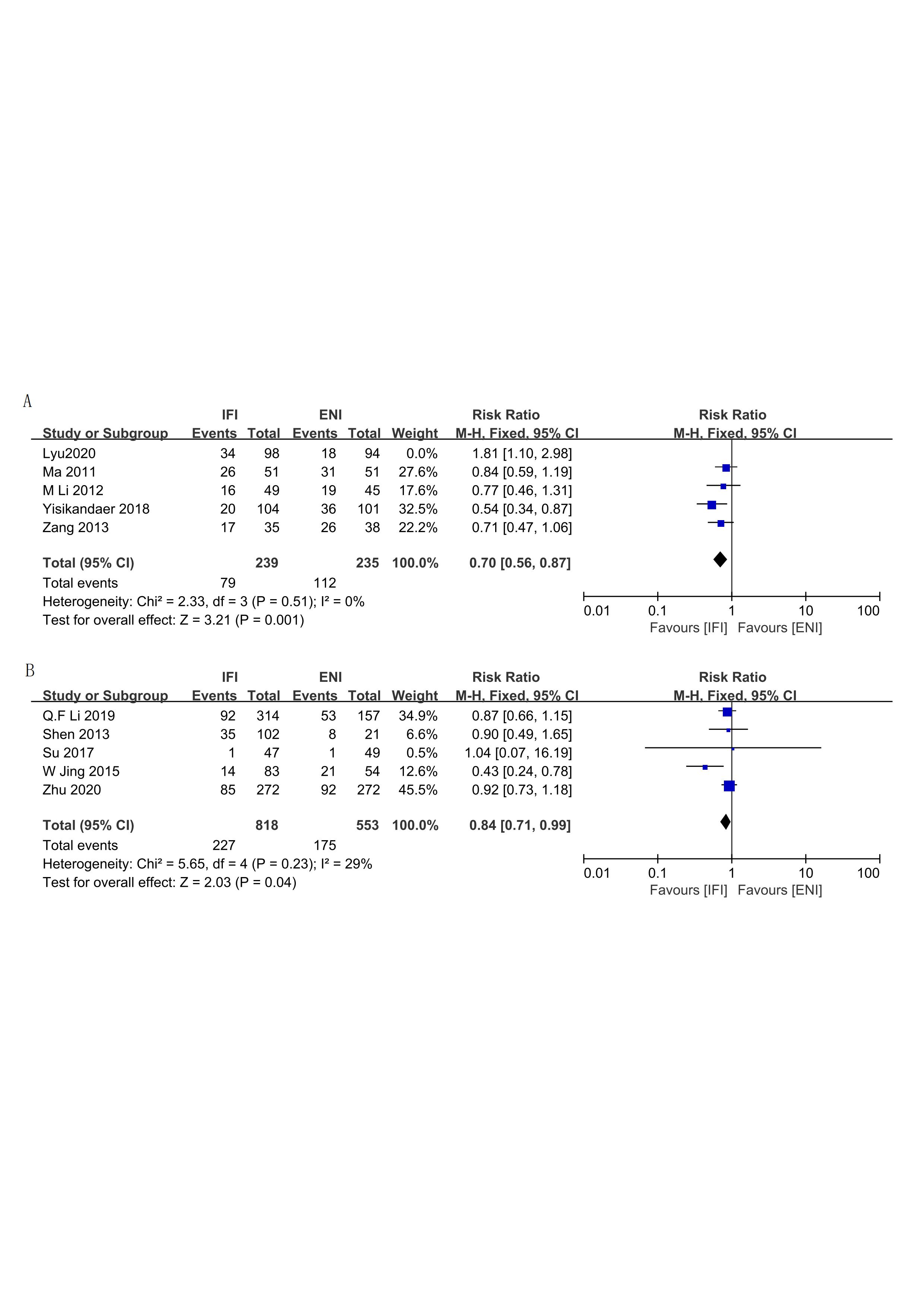

Supplement: Supplementary file 1 [file DataSheet_1.zip › supplementary materials/Supplementary Figure/Supplementary Figure. 15_00.jpg]

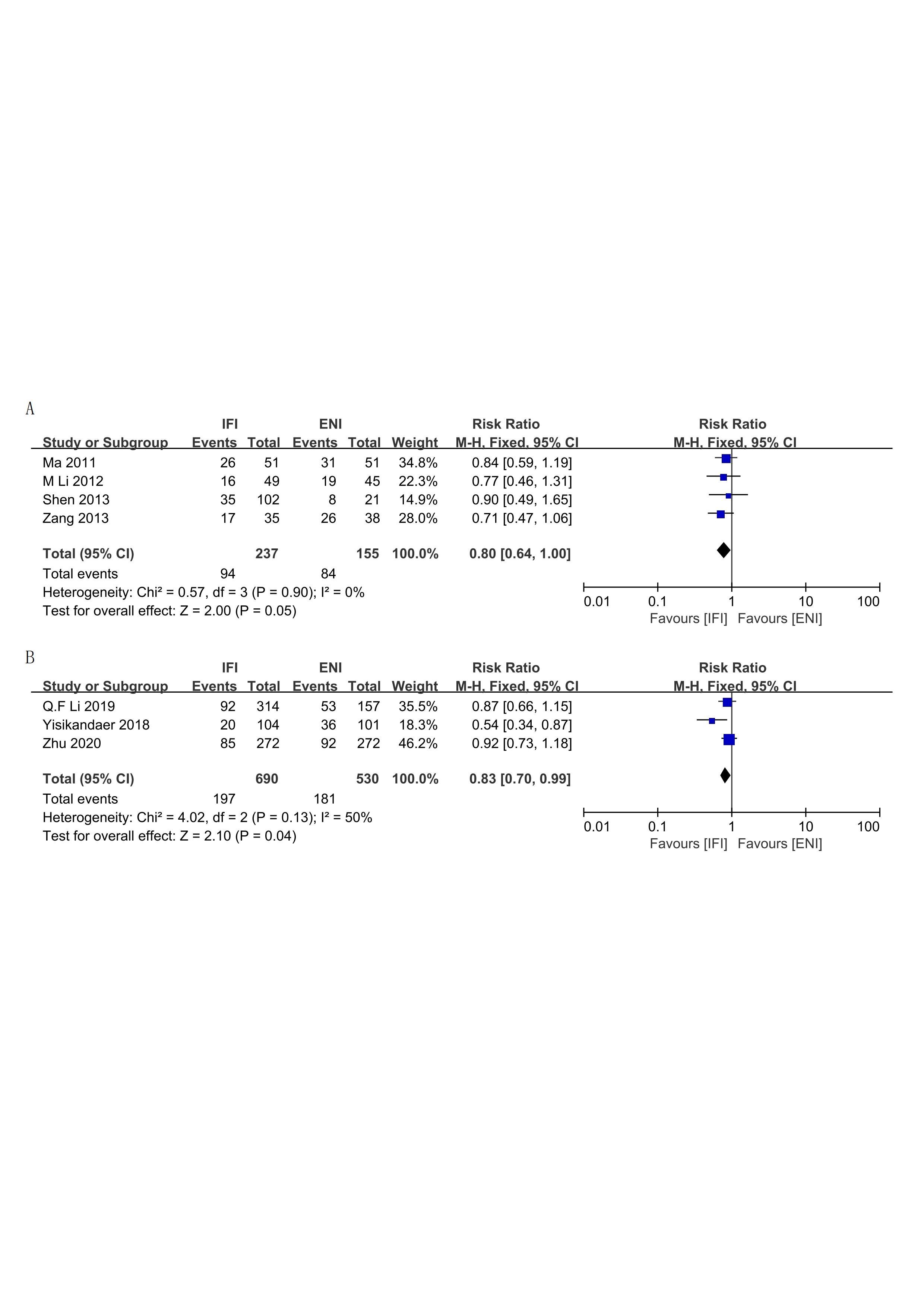

Supplement: Supplementary file 1 [file DataSheet_1.zip › supplementary materials/Supplementary Figure/Supplementary Figure. 16_00.jpg]

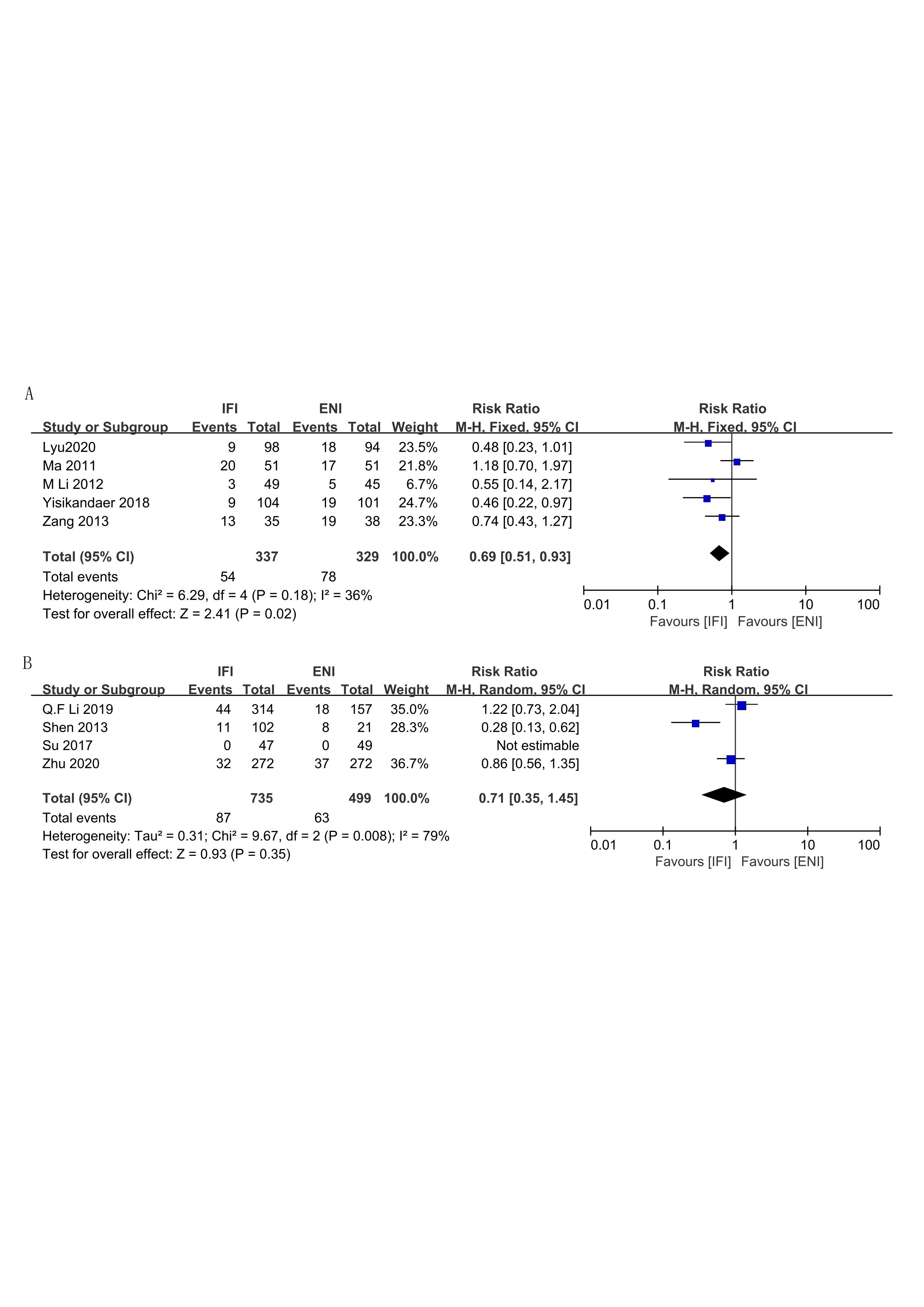

Supplement: Supplementary file 1 [file DataSheet_1.zip › supplementary materials/Supplementary Figure/Supplementary Figure. 17_00.jpg]

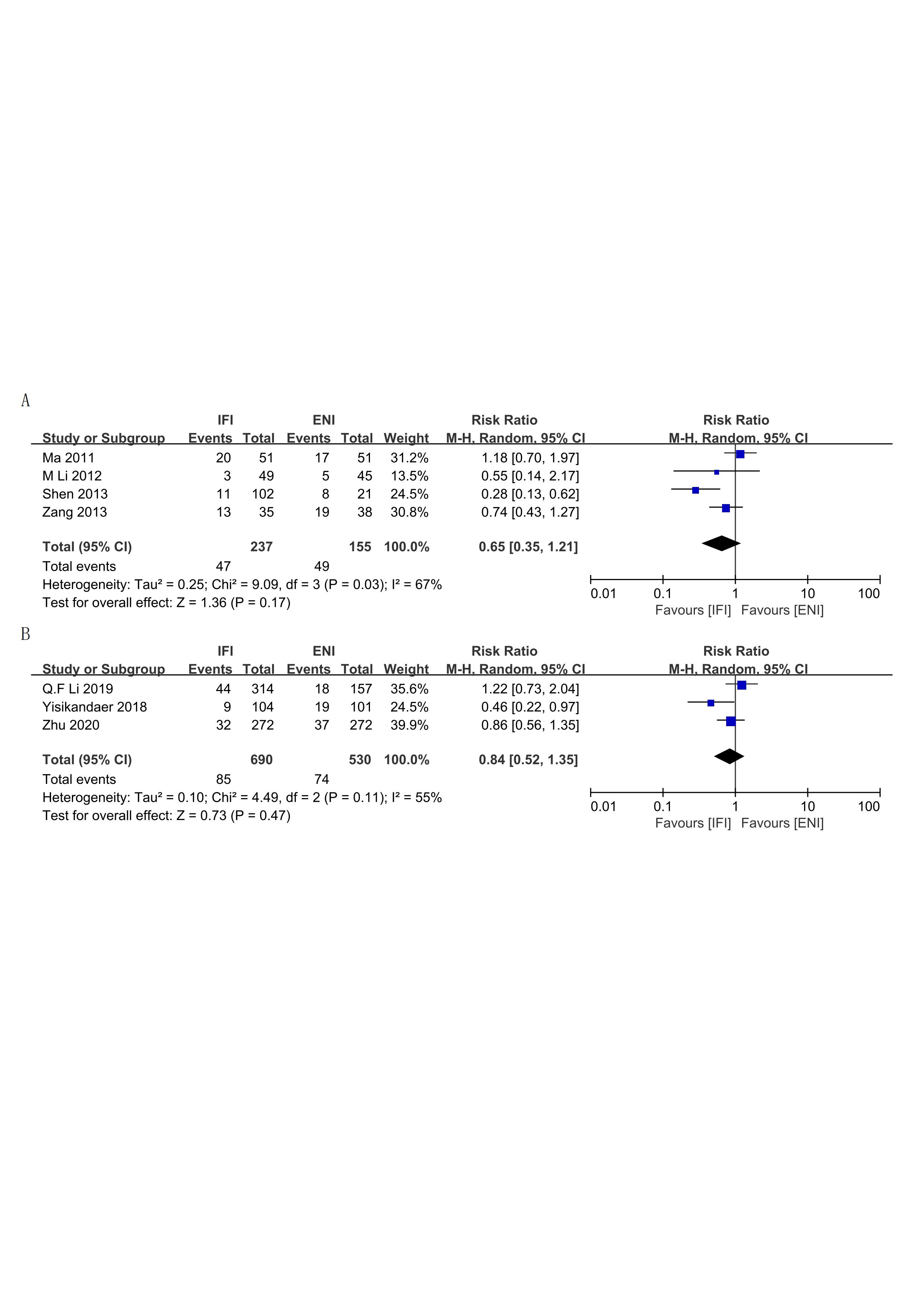

Supplement: Supplementary file 1 [file DataSheet_1.zip › supplementary materials/Supplementary Figure/Supplementary Figure. 18_00.jpg]

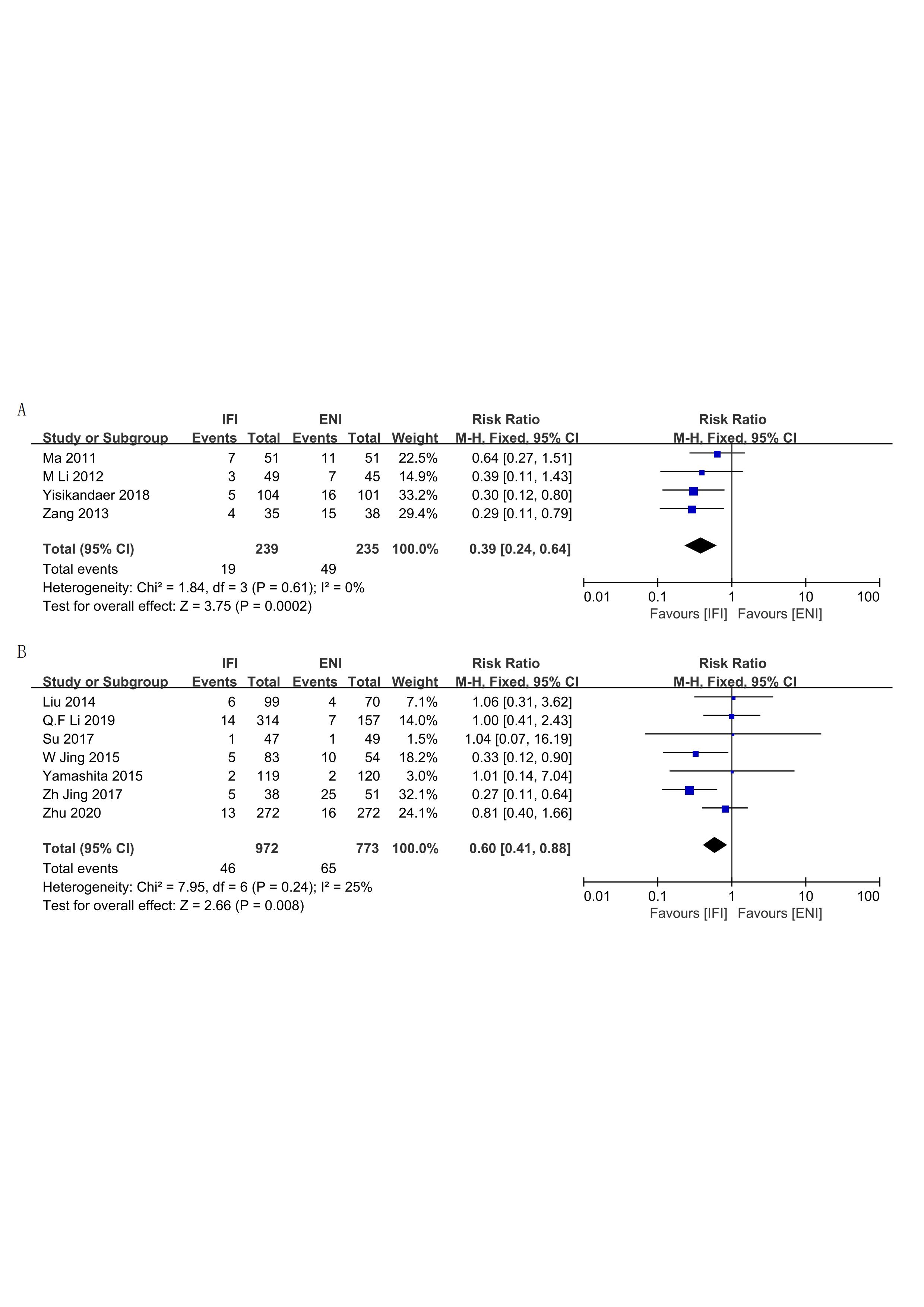

Supplement: Supplementary file 1 [file DataSheet_1.zip › supplementary materials/Supplementary Figure/Supplementary Figure. 19_00.jpg]

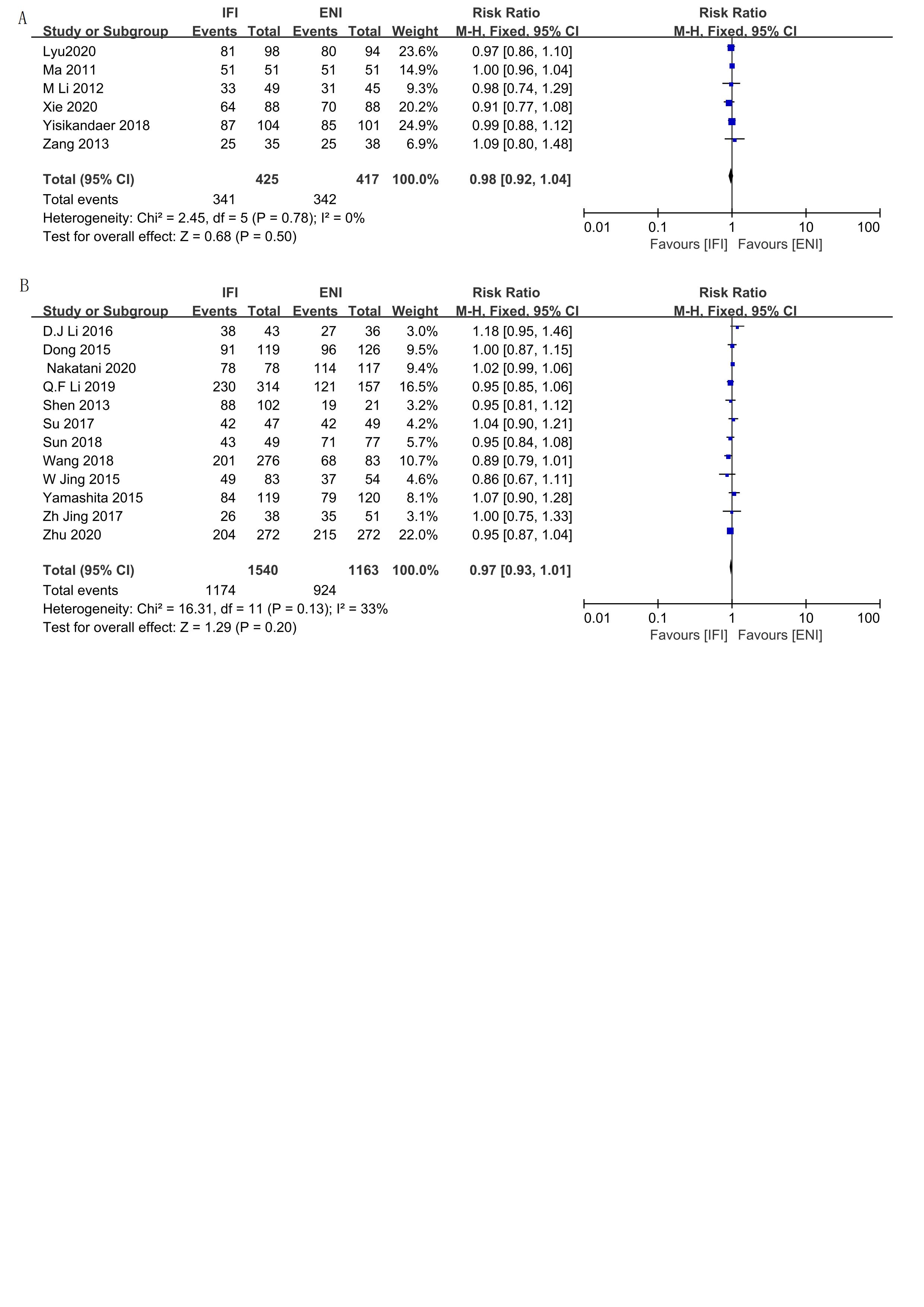

Supplement: Supplementary file 1 [file DataSheet_1.zip › supplementary materials/Supplementary Figure/Supplementary Figure. 1_00.jpg]

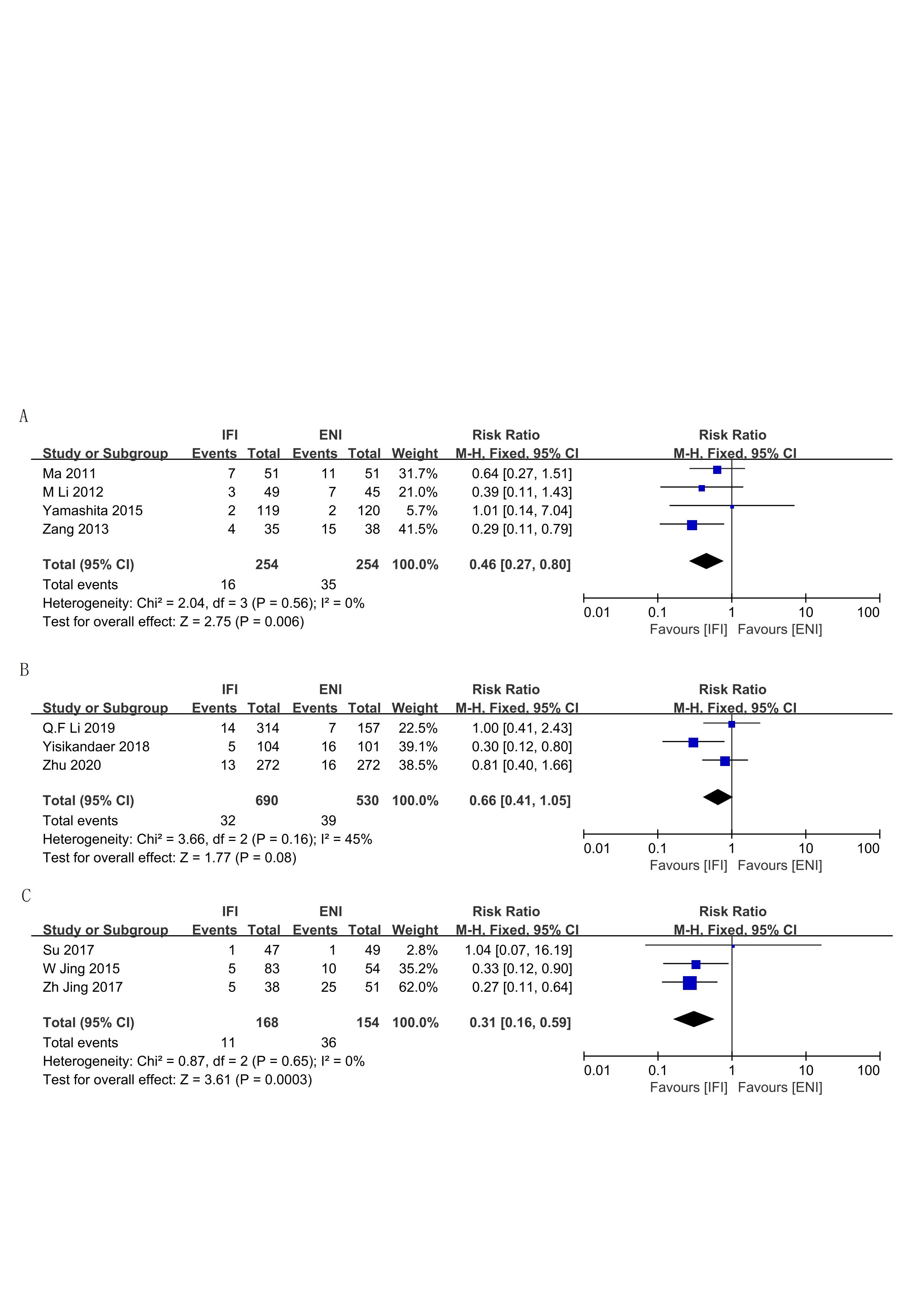

Supplement: Supplementary file 1 [file DataSheet_1.zip › supplementary materials/Supplementary Figure/Supplementary Figure. 20_00.jpg]

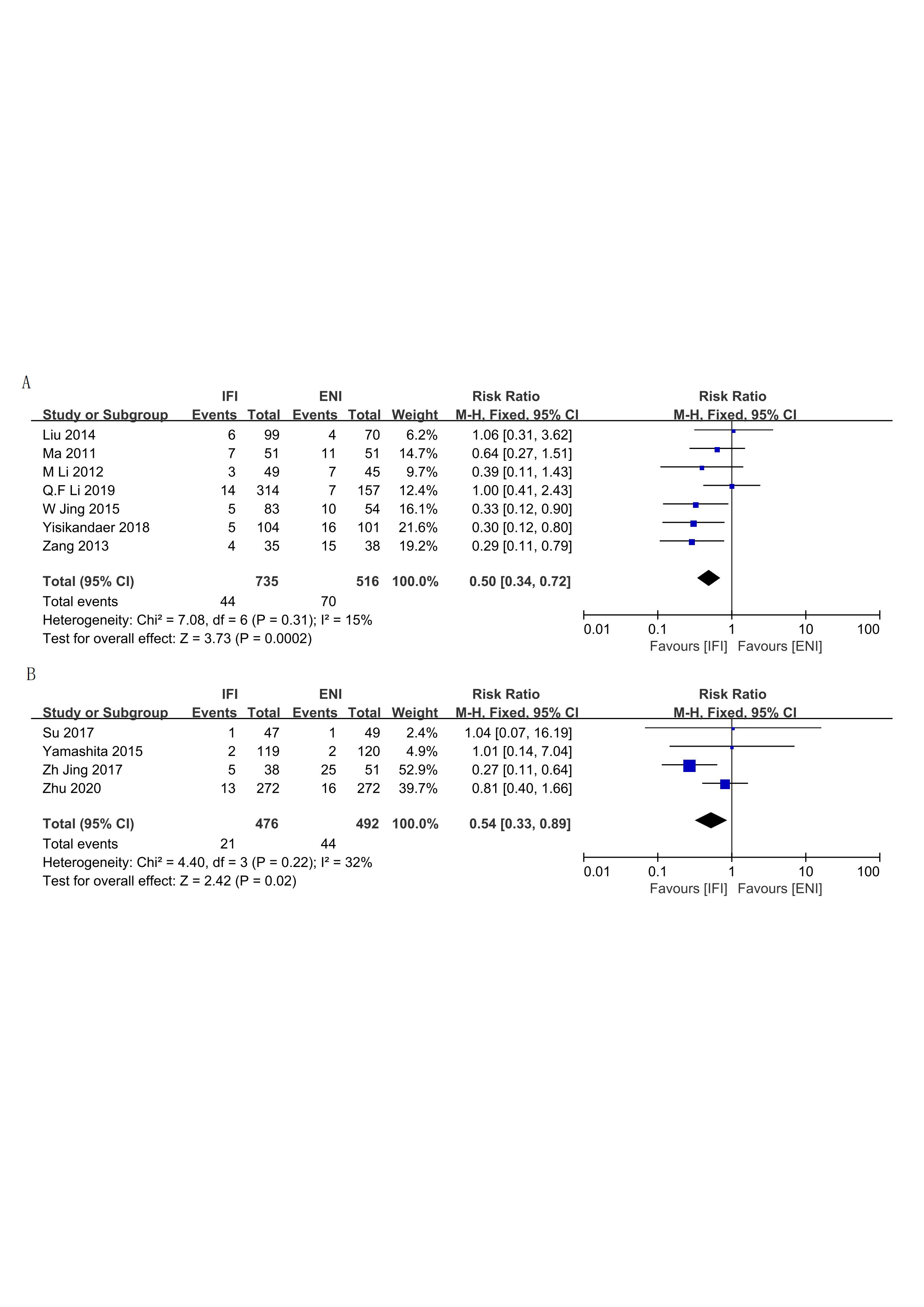

Supplement: Supplementary file 1 [file DataSheet_1.zip › supplementary materials/Supplementary Figure/Supplementary Figure. 21_00.jpg]

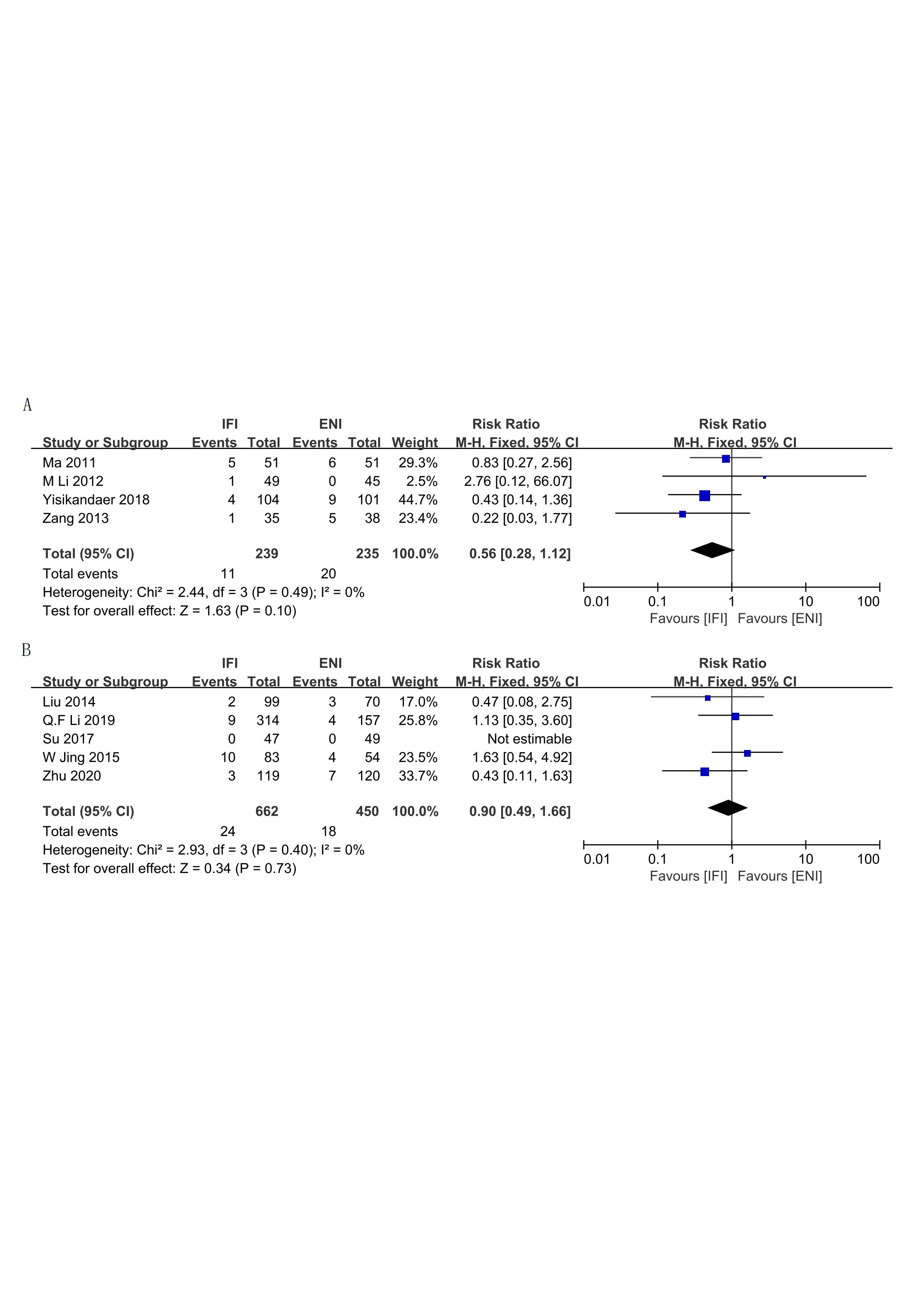

Supplement: Supplementary file 1 [file DataSheet_1.zip › supplementary materials/Supplementary Figure/Supplementary Figure. 22_00.jpg]

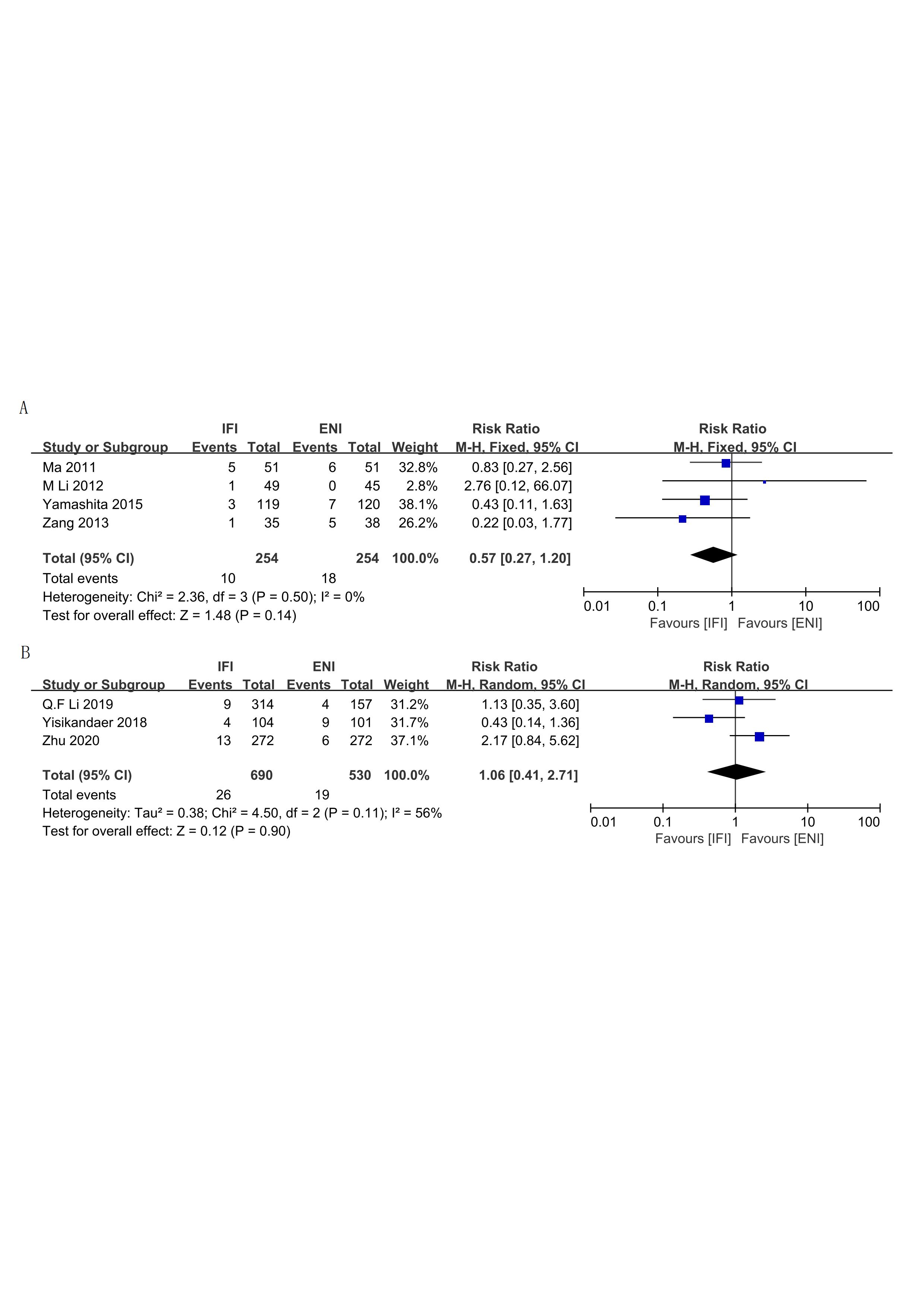

Supplement: Supplementary file 1 [file DataSheet_1.zip › supplementary materials/Supplementary Figure/Supplementary Figure. 23_00.jpg]

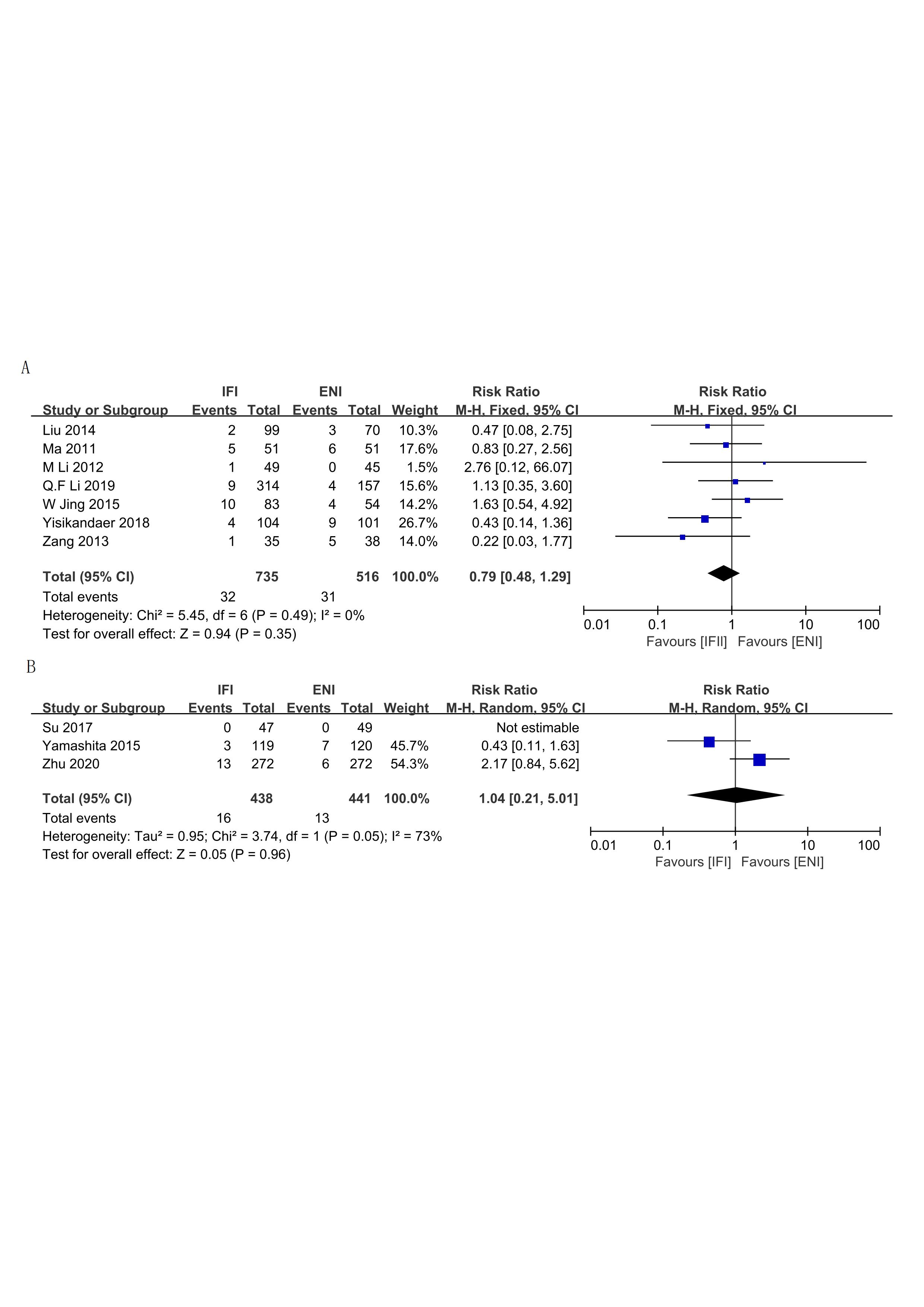

Supplement: Supplementary file 1 [file DataSheet_1.zip › supplementary materials/Supplementary Figure/Supplementary Figure. 24_00.jpg]

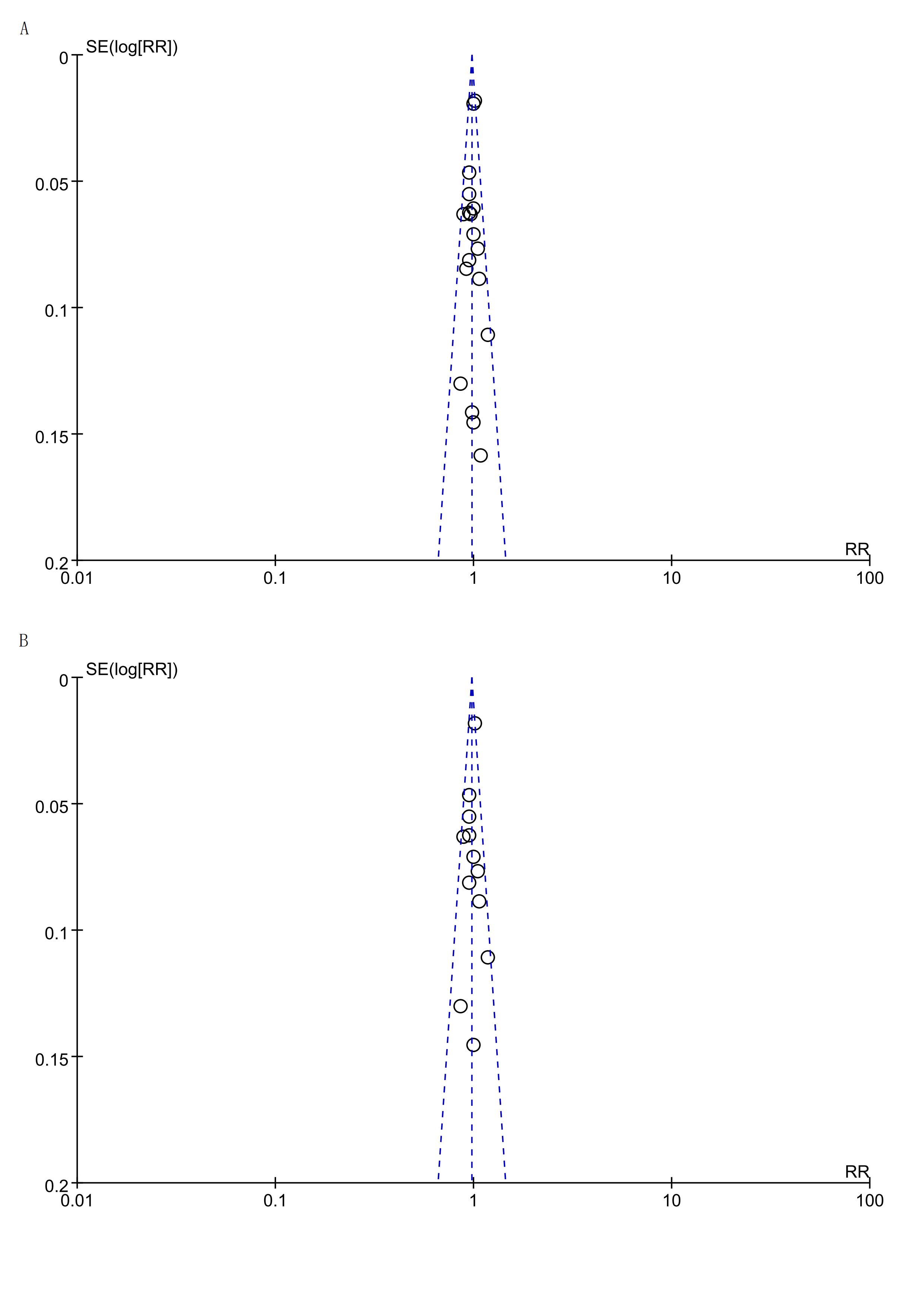

Supplement: Supplementary file 1 [file DataSheet_1.zip › supplementary materials/Supplementary Figure/Supplementary Figure. 25_00.jpg]

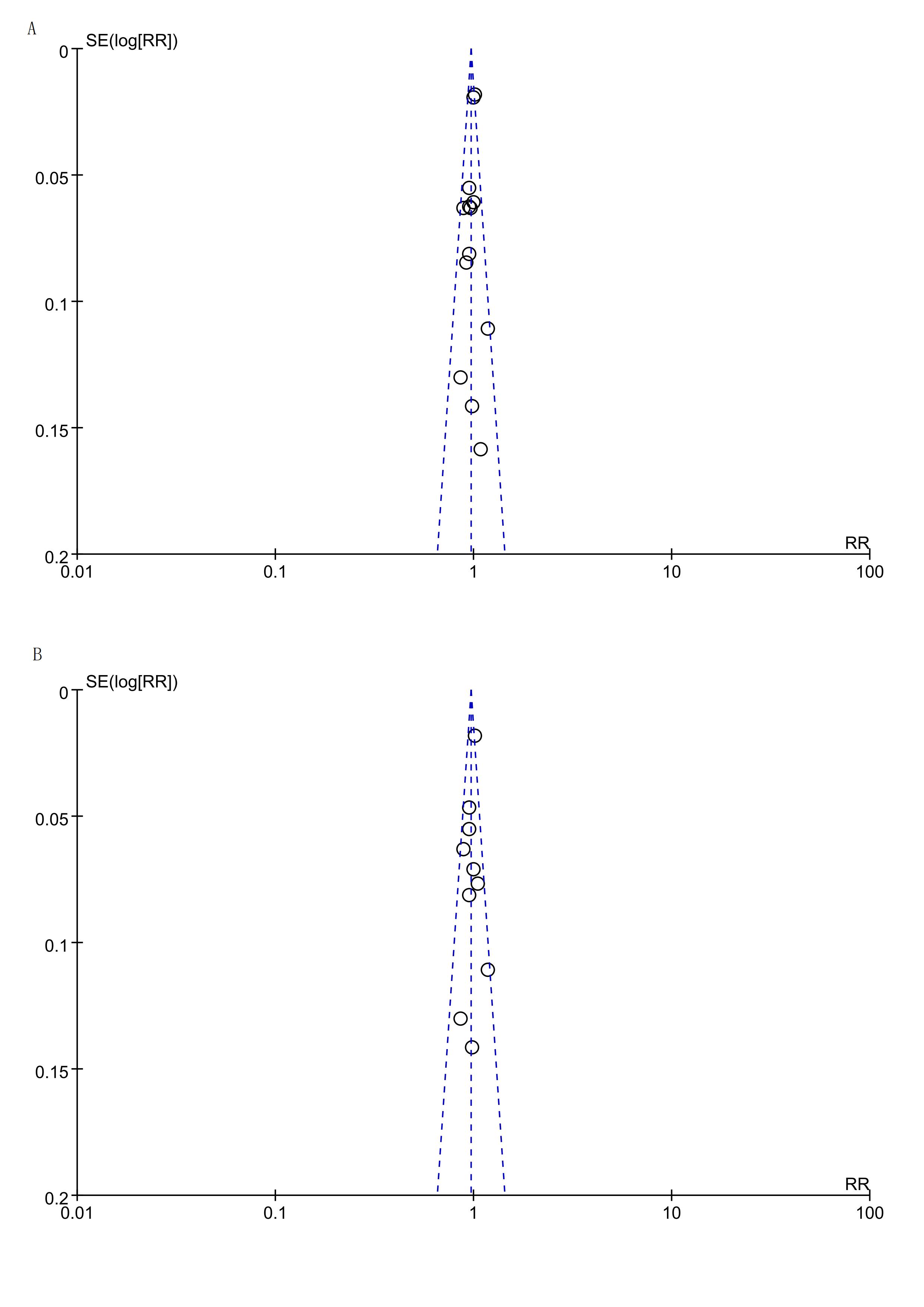

Supplement: Supplementary file 1 [file DataSheet_1.zip › supplementary materials/Supplementary Figure/Supplementary Figure. 26_00.jpg]

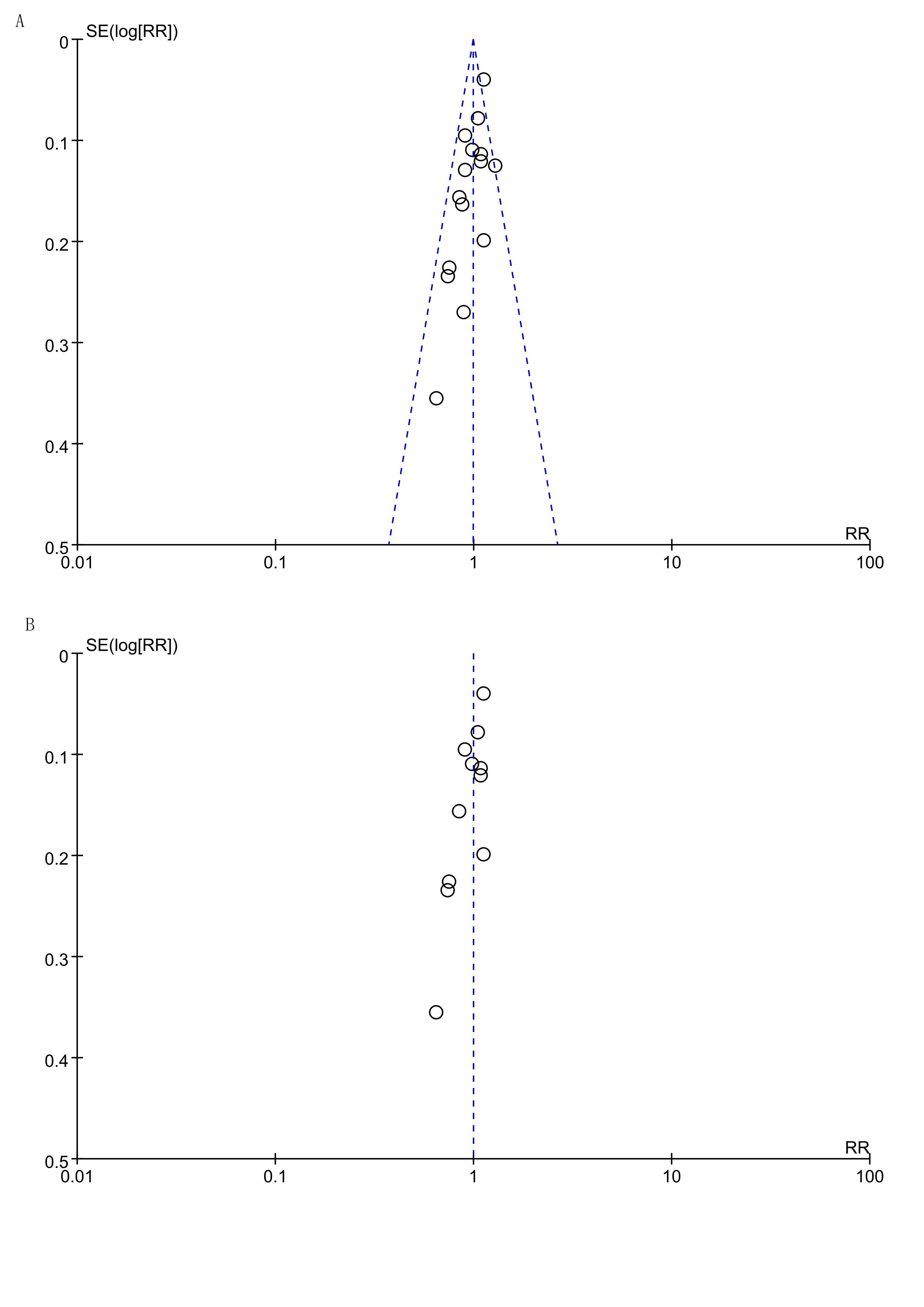

Supplement: Supplementary file 1 [file DataSheet_1.zip › supplementary materials/Supplementary Figure/Supplementary Figure. 27_00.jpg]

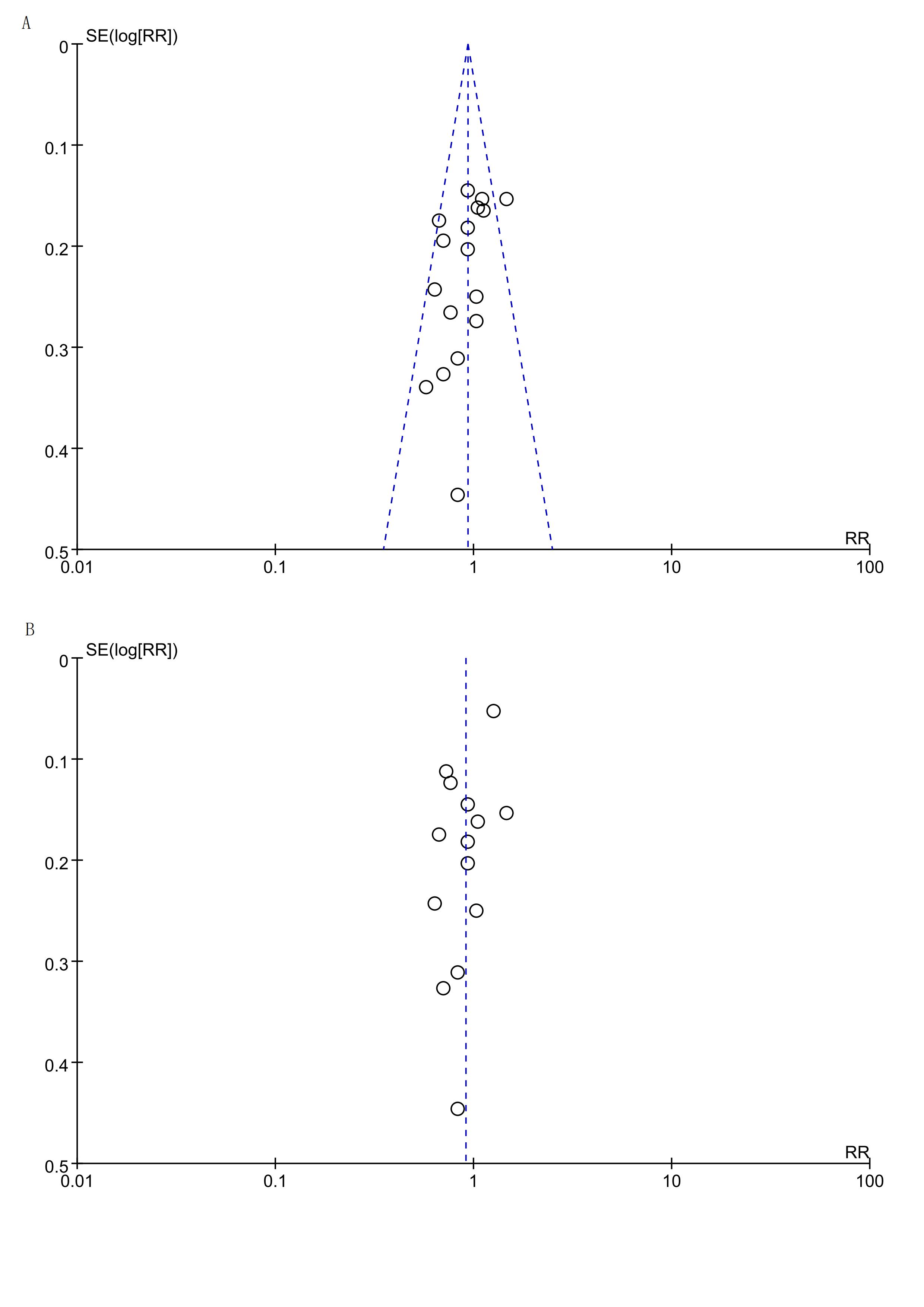

Supplement: Supplementary file 1 [file DataSheet_1.zip › supplementary materials/Supplementary Figure/Supplementary Figure. 28_00.jpg]

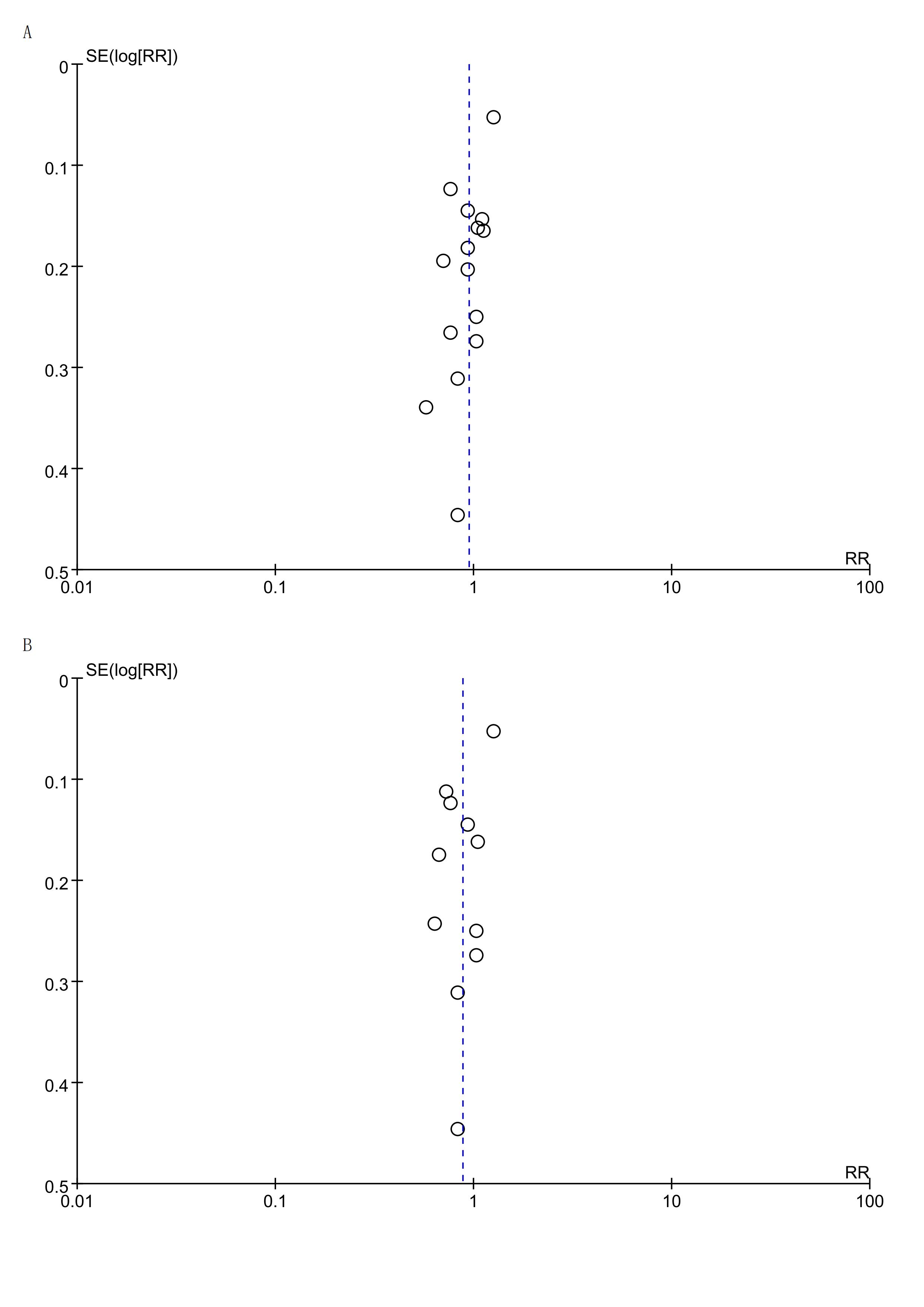

Supplement: Supplementary file 1 [file DataSheet_1.zip › supplementary materials/Supplementary Figure/Supplementary Figure. 29_00.jpg]

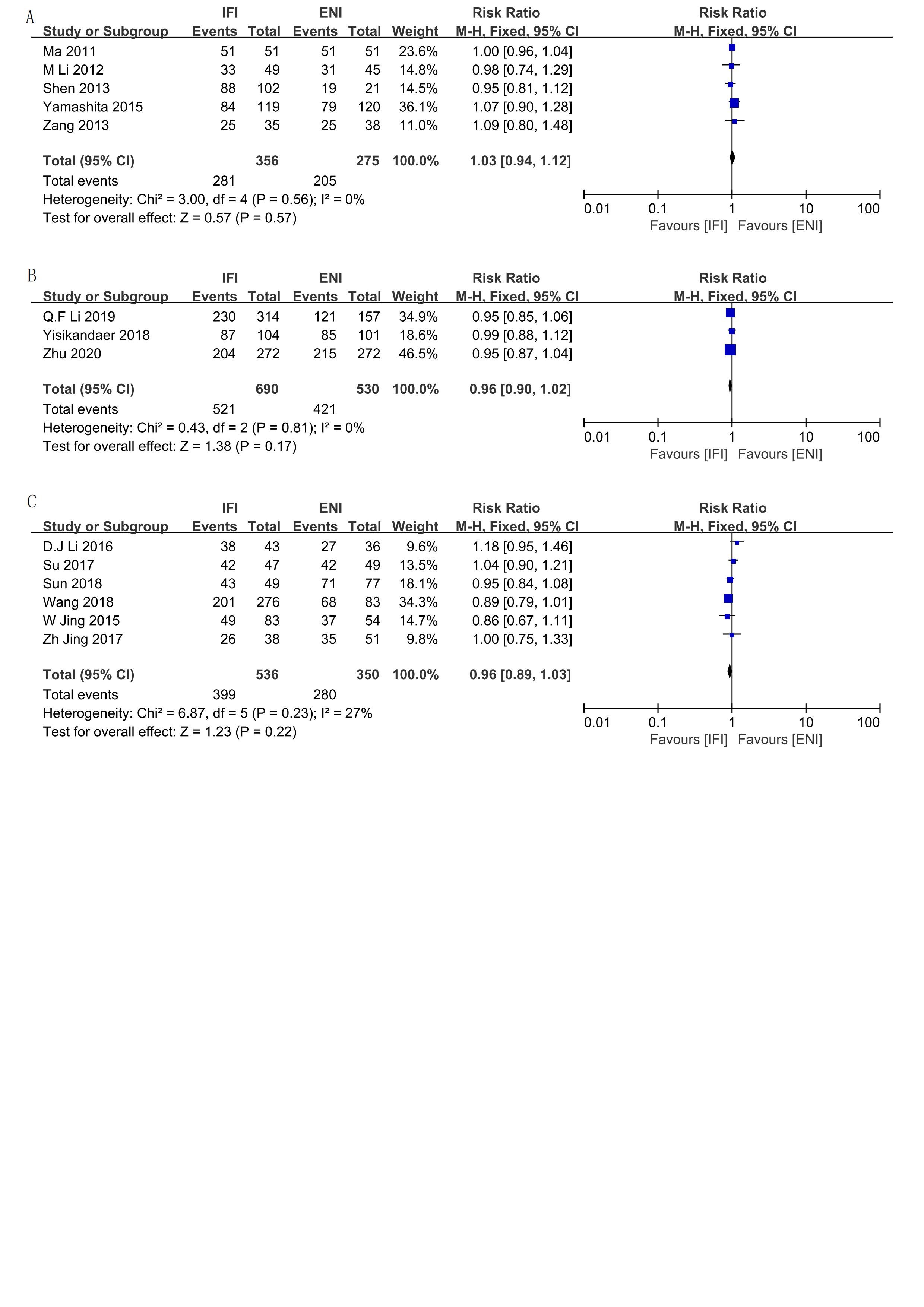

Supplement: Supplementary file 1 [file DataSheet_1.zip › supplementary materials/Supplementary Figure/Supplementary Figure. 2_00.jpg]

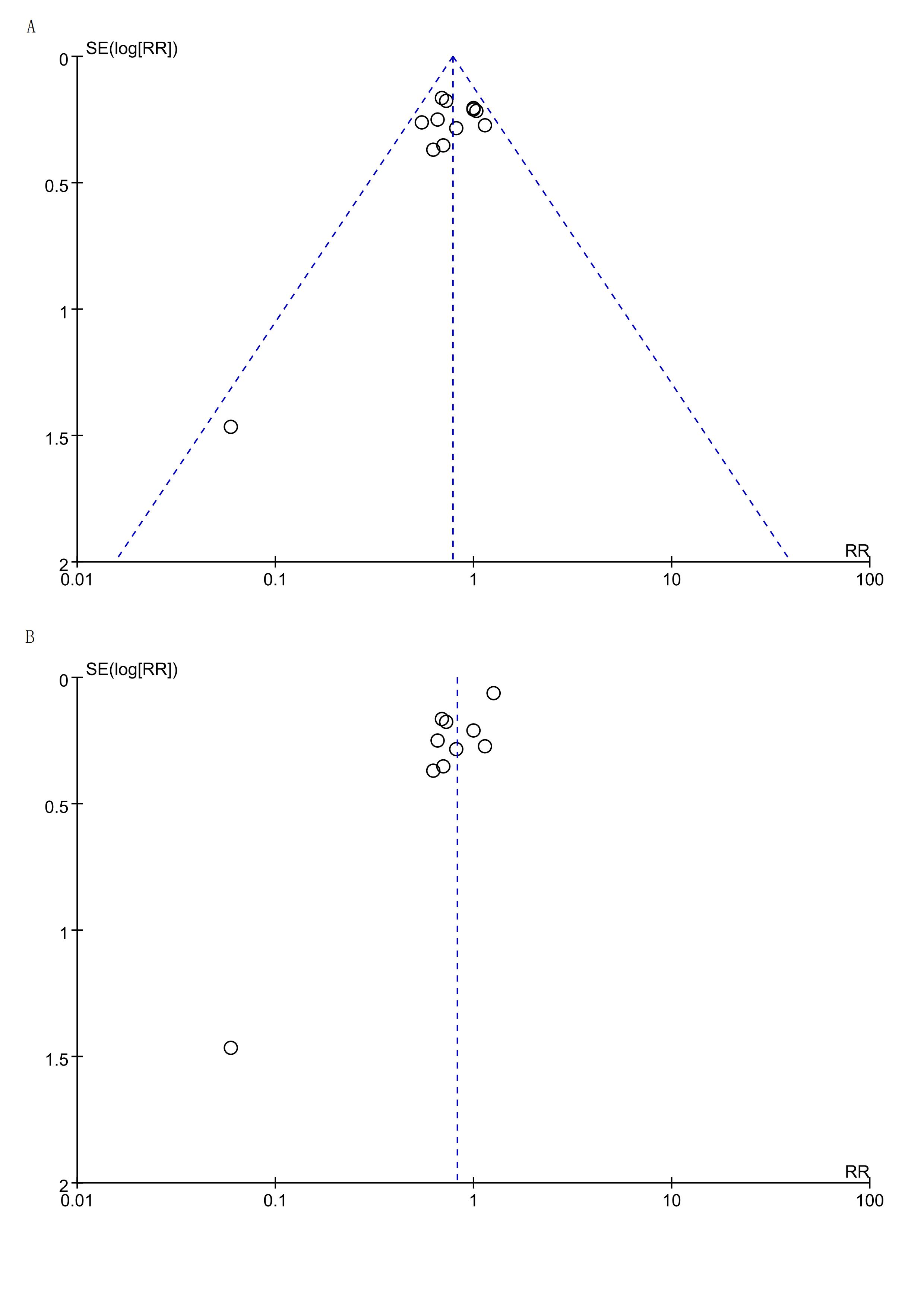

Supplement: Supplementary file 1 [file DataSheet_1.zip › supplementary materials/Supplementary Figure/Supplementary Figure. 30_00.jpg]

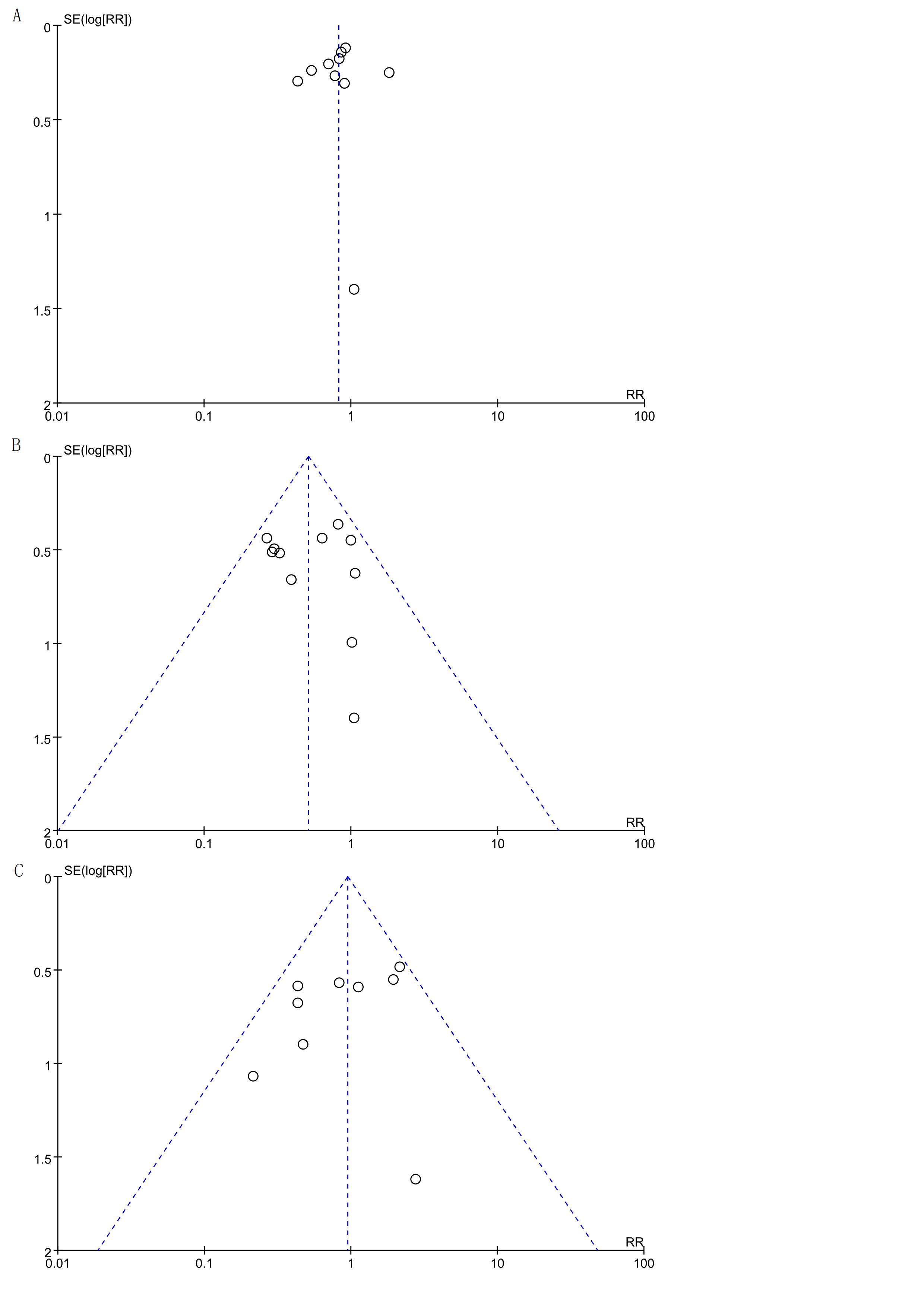

Supplement: Supplementary file 1 [file DataSheet_1.zip › supplementary materials/Supplementary Figure/Supplementary Figure. 31_00.jpg]

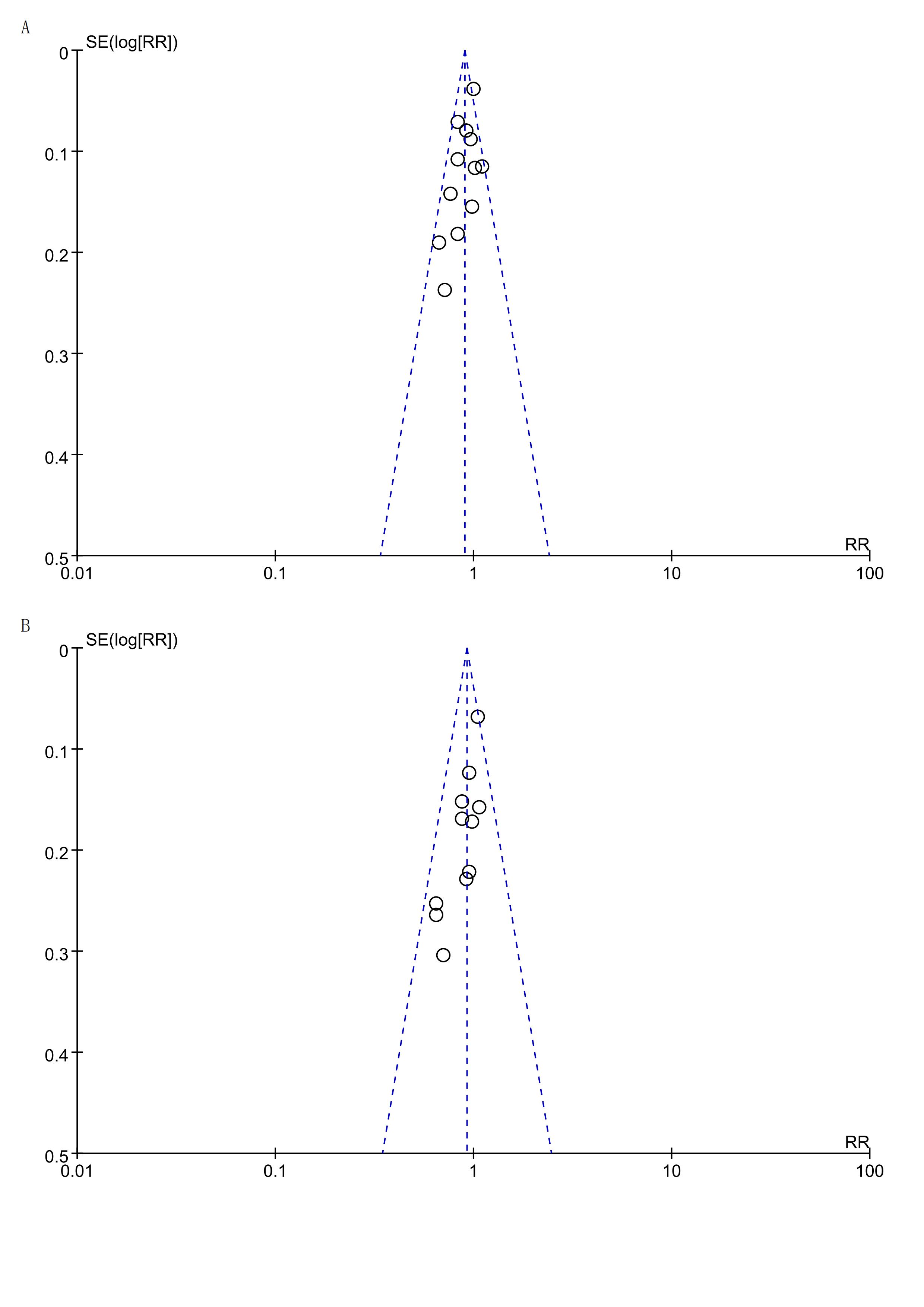

Supplement: Supplementary file 1 [file DataSheet_1.zip › supplementary materials/Supplementary Figure/Supplementary Figure. 32_00.jpg]

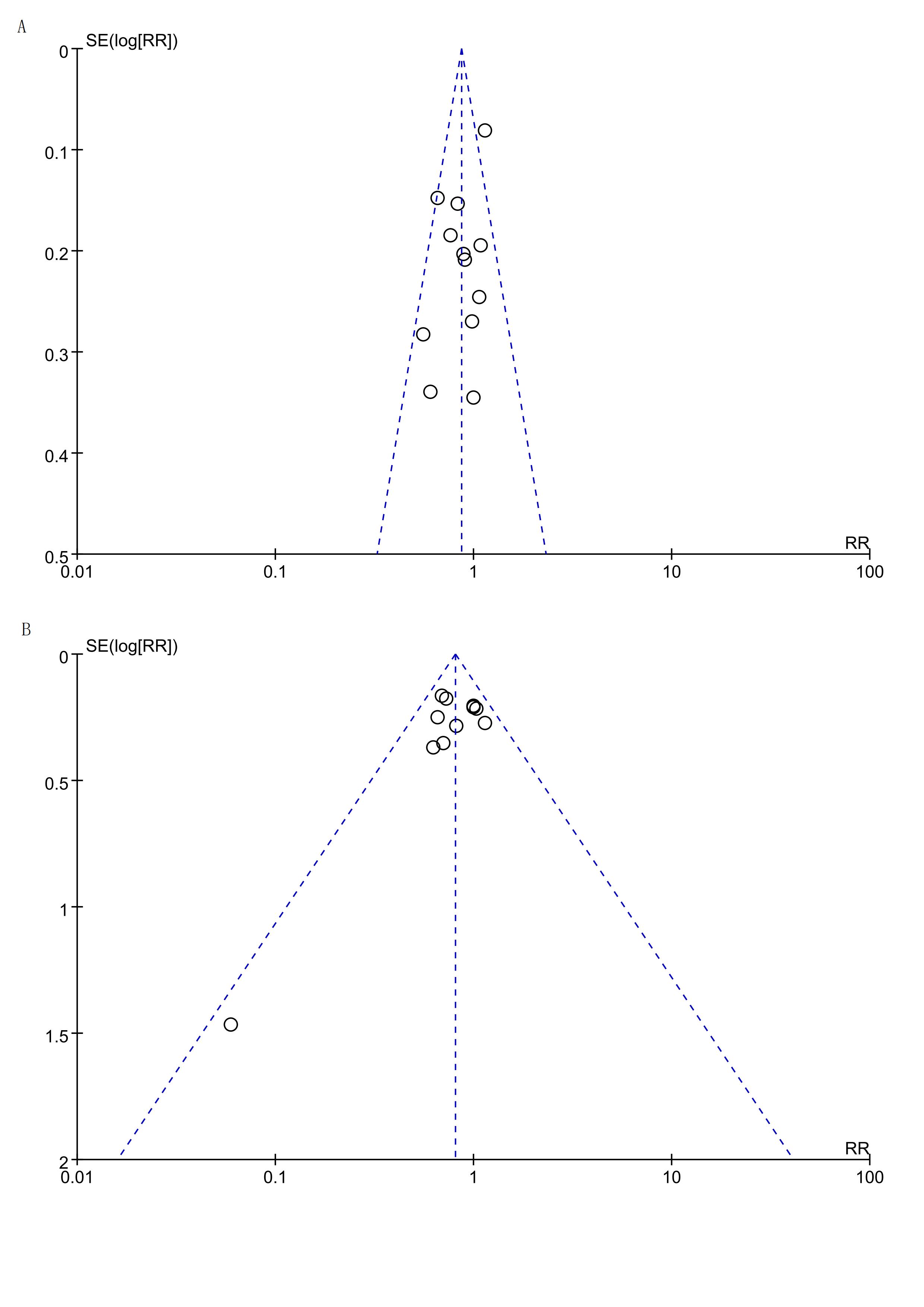

Supplement: Supplementary file 1 [file DataSheet_1.zip › supplementary materials/Supplementary Figure/Supplementary Figure. 33_00.jpg]

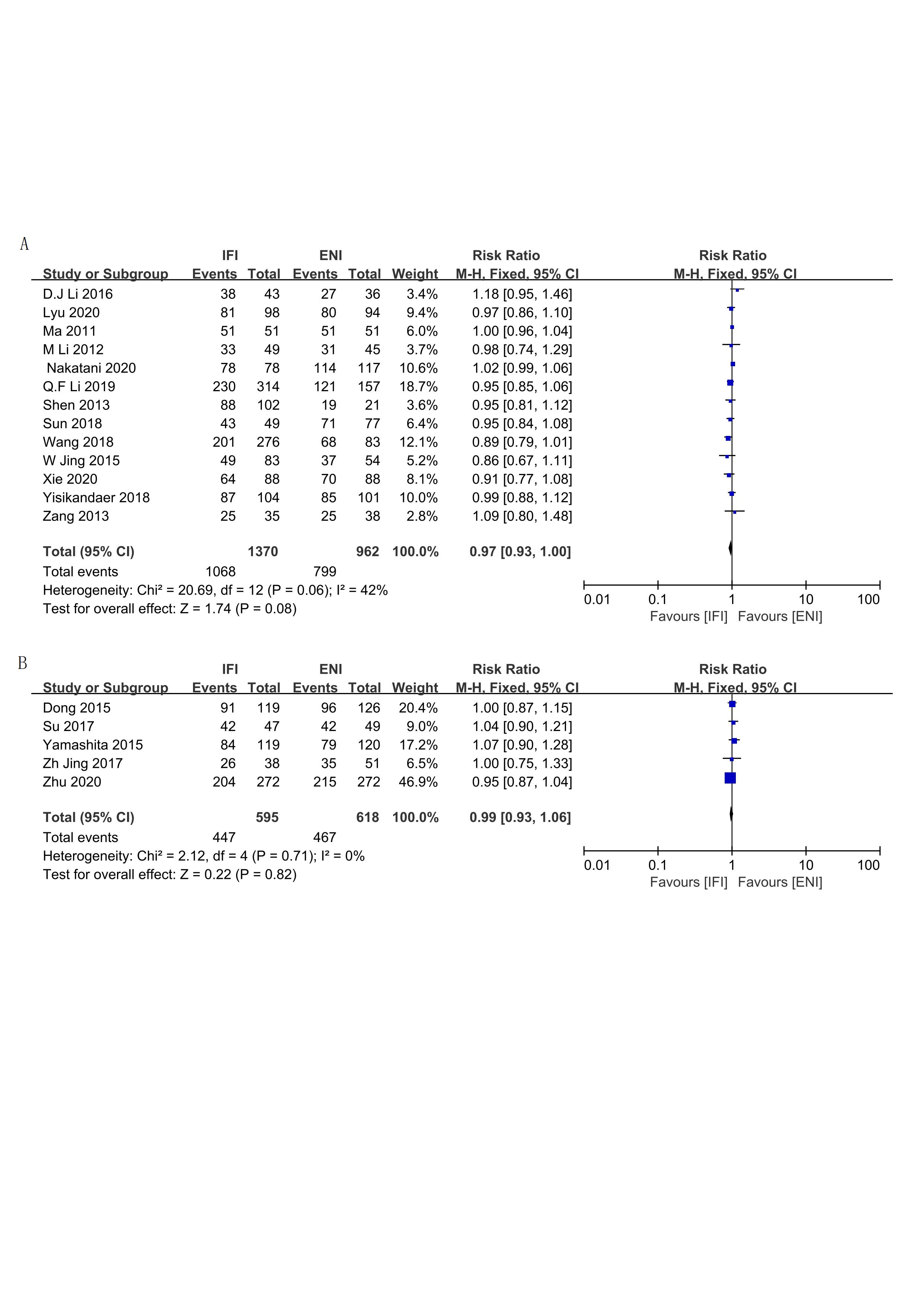

Supplement: Supplementary file 1 [file DataSheet_1.zip › supplementary materials/Supplementary Figure/Supplementary Figure. 3_00.jpg]

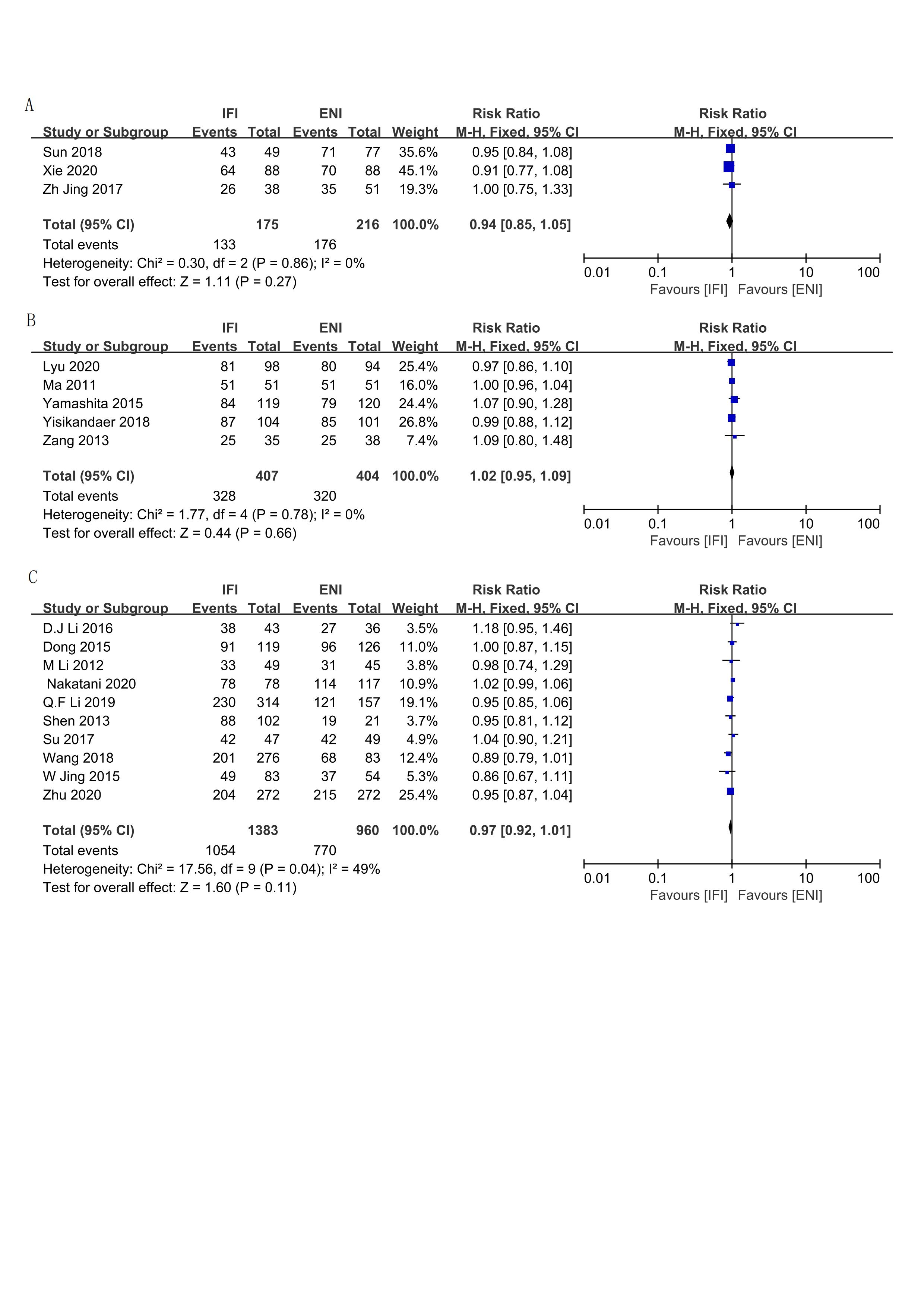

Supplement: Supplementary file 1 [file DataSheet_1.zip › supplementary materials/Supplementary Figure/Supplementary Figure. 4_00.jpg]

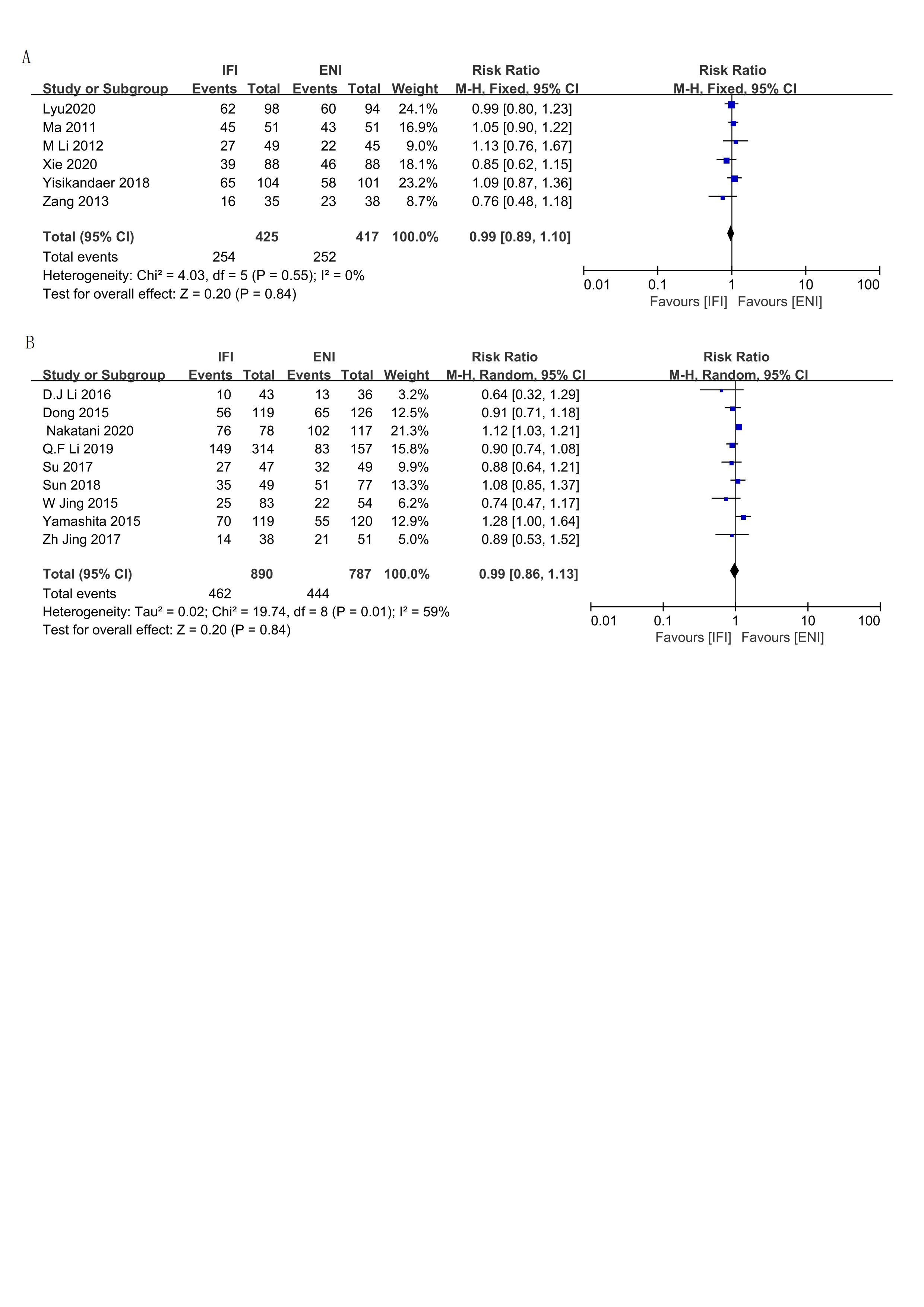

Supplement: Supplementary file 1 [file DataSheet_1.zip › supplementary materials/Supplementary Figure/Supplementary Figure. 5_00.jpg]

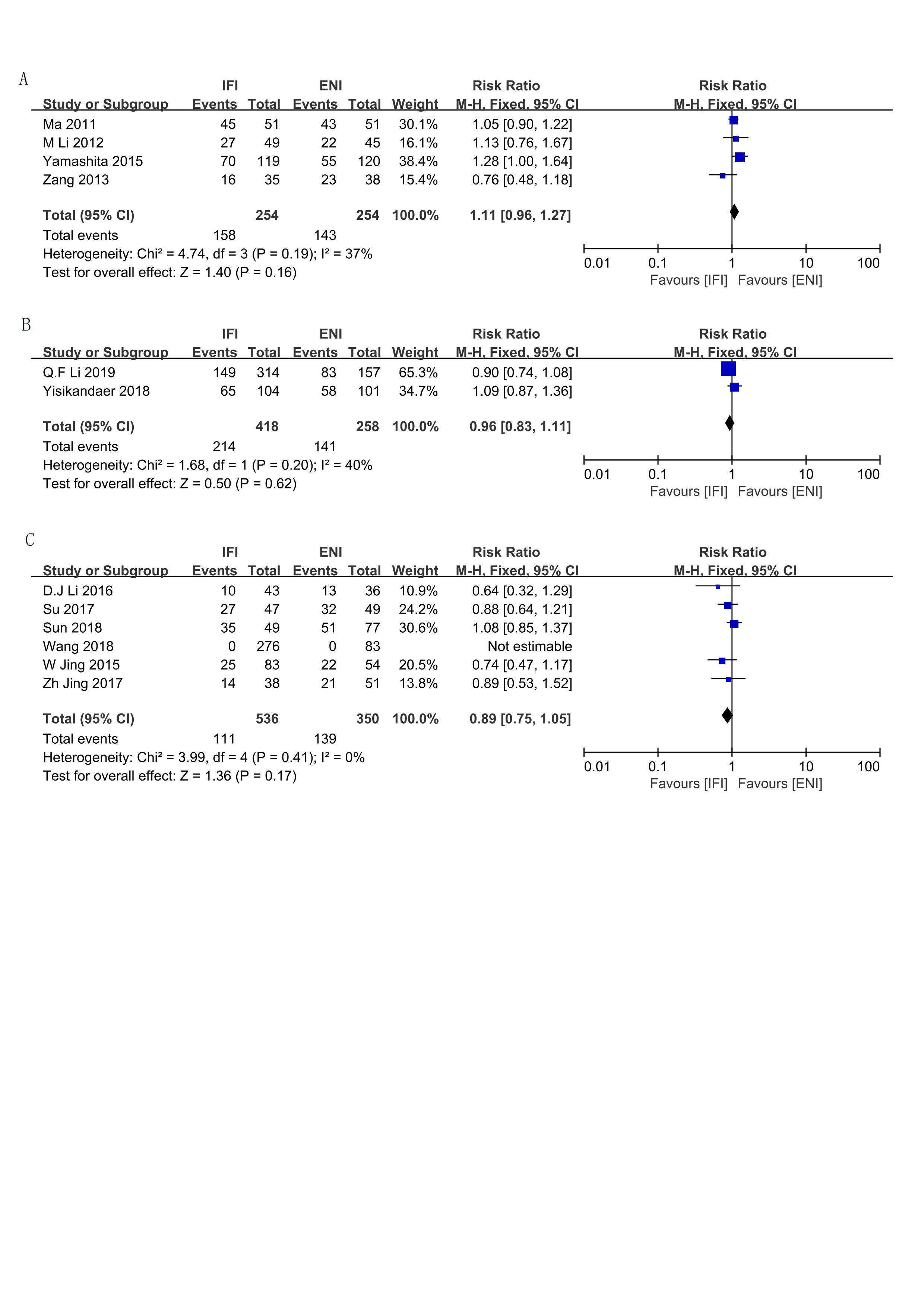

Supplement: Supplementary file 1 [file DataSheet_1.zip › supplementary materials/Supplementary Figure/Supplementary Figure. 6_00.jpg]

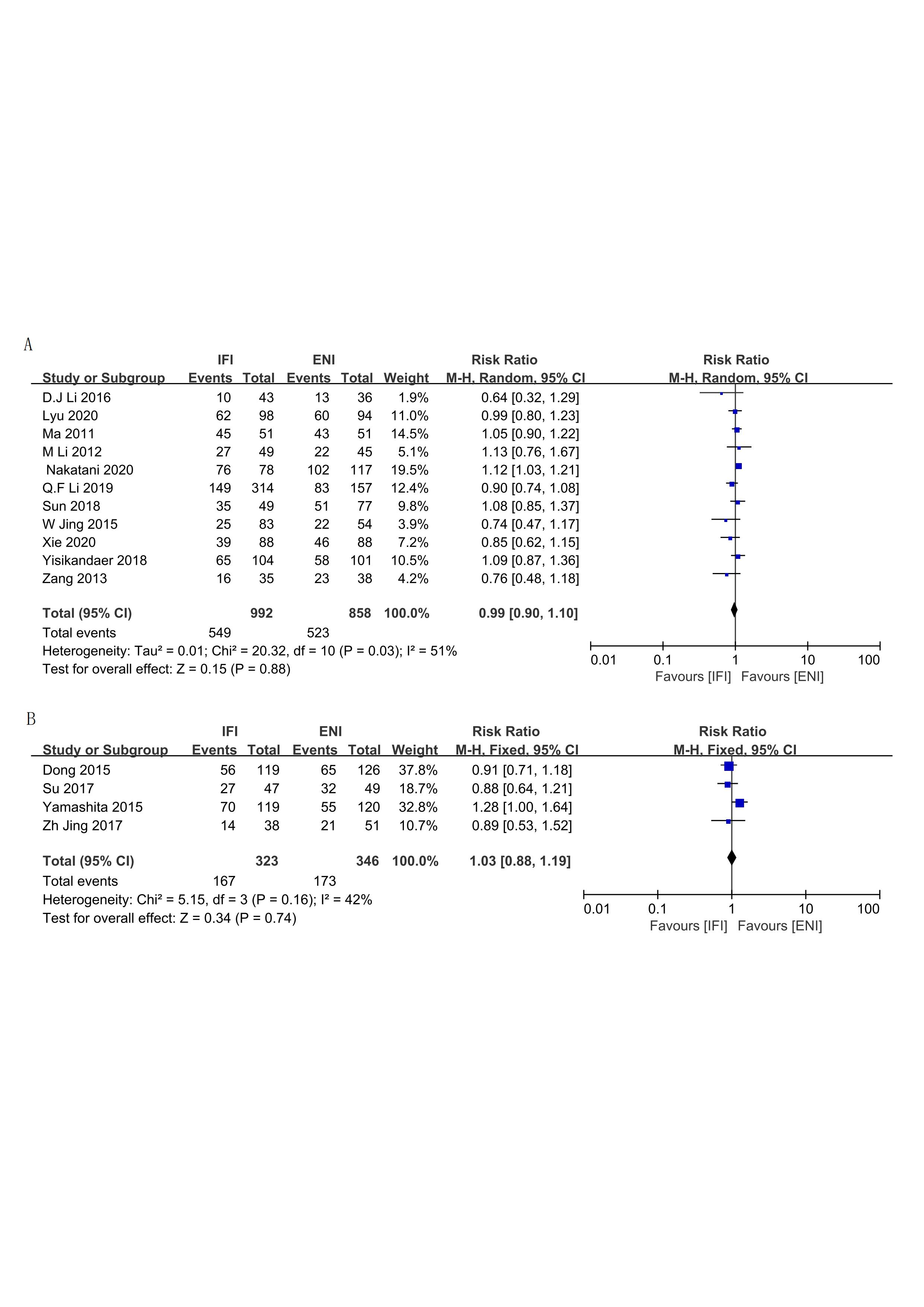

Supplement: Supplementary file 1 [file DataSheet_1.zip › supplementary materials/Supplementary Figure/Supplementary Figure. 7_00.jpg]

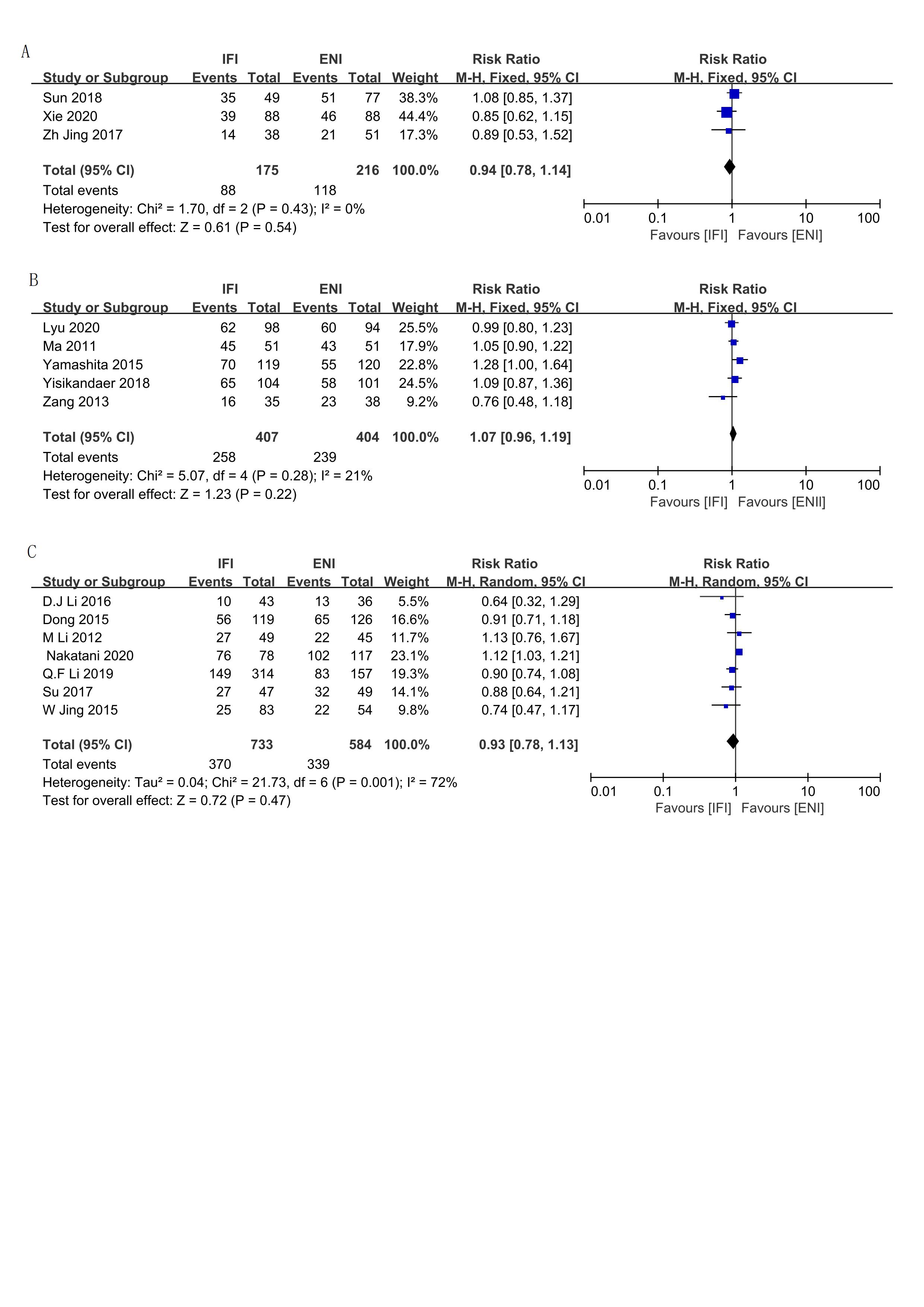

Supplement: Supplementary file 1 [file DataSheet_1.zip › supplementary materials/Supplementary Figure/Supplementary Figure. 8_00.jpg]

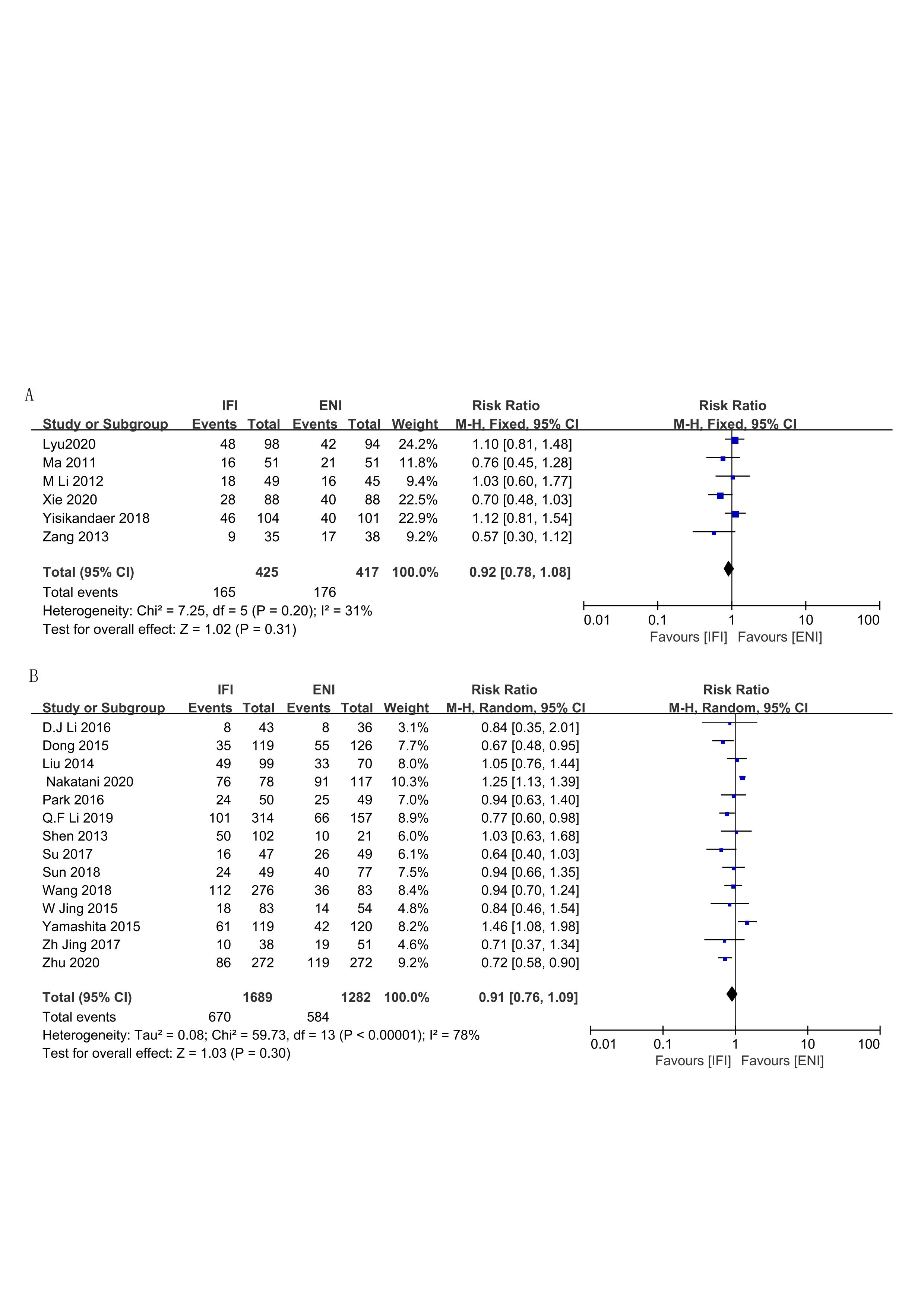

Supplement: Supplementary file 1 [file DataSheet_1.zip › supplementary materials/Supplementary Figure/Supplementary Figure. 9_00.jpg]
